# Fractional killing arises from cell-to-cell variability in overcoming a caspase activity threshold

**DOI:** 10.15252/msb.20145584

**Published:** 2015-05-07

**Authors:** Jérémie Roux, Marc Hafner, Samuel Bandara, Joshua J Sims, Hannah Hudson, Diana Chai, Peter K Sorger

**Affiliations:** 1 Department of Systems Biology, Harvard Medical SchoolBoston, MA, USA; 2 Merrimack PharmaceuticalsCambridge, MA, USA

**Keywords:** anti-cancer therapeutic antibodies, apoptosis, DR4, DR5 receptors, programmed cell death, TRAIL

## Abstract

When cells are exposed to death ligands such as TRAIL, a fraction undergoes apoptosis and a fraction survives; if surviving cells are re-exposed to TRAIL, fractional killing is once again observed. Therapeutic antibodies directed against TRAIL receptors also cause fractional killing, even at saturating concentrations, limiting their effectiveness. Fractional killing arises from cell-to-cell fluctuations in protein levels (extrinsic noise), but how this results in a clean bifurcation between life and death remains unclear. In this paper, we identify a threshold in the rate and timing of initiator caspase activation that distinguishes cells that live from those that die; by mapping this threshold, we can predict fractional killing of cells exposed to natural and synthetic agonists alone or in combination with sensitizing drugs such as bortezomib. A phenomenological model of the threshold also quantifies the contributions of two resistance genes (c-FLIP and Bcl-2), providing new insight into the control of cell fate by opposing pro-death and pro-survival proteins and suggesting new criteria for evaluating the efficacy of therapeutic TRAIL receptor agonists.

## Introduction

Activation of death receptors (DR4 and DR5) by tumor necrosis factor-related apoptosis inducing ligand (TRAIL) and by antibody agonists of DR4/5 triggers apoptosis by activating a cascade of initiator (caspases-8 and 10; hereafter C8) and effector caspases (caspases-3 and 7; hereafter C3). The selectivity of TRAIL for tumor cells over normal cells and the ability of death receptors to rapidly induce apoptosis underlie the interest in DR4/5 agonists as anti-cancer drugs. Multiple recombinant and humanized anti-DR4/5 therapeutic antibodies have been developed and tested, and they are generally well tolerated. However, these drugs have been disappointing in clinical trials, largely due to insufficient potency. Many once-promising drug development programs have been terminated prior to pivotal Phase III trials (Holland, 2014). The challenge in using DR4/5 agonists as drugs is that the molecular basis of sensitivity and resistance to TRAIL is poorly understood (Falschlehner *et al*, 2007; Gonzalvez & Ashkenazi, 2010), particularly with respect to incomplete (fractional) cell killing and low maximum drug effect (*E*_max_).

Many mechanisms of TRAIL resistance have been described (Dimberg *et al*, 2013), but their relative significance in controlling ligand and drug response in different cell types has not yet been established. Determining whether there is potential to resurrect TRAIL agonists as a therapeutic class will require a quantitative means to evaluate the net effect of changes in agonist type and oligomeric state, receptor expression levels, activity of resistance genes, and induction of pro-survival pathways (Spencer & Sorger, 2011). In this paper, we take a first step in this direction by characterizing the activities of caspases in single cells exposed to natural and synthetic TRAIL receptor agonists, including several molecules developed as therapeutic agents, in the presence and absence of overexpressed resistance genes. We develop a simple, semi-mechanistic model of the resulting data and show that it describes a life/death threshold operating at the level of initiator caspase activation.

Extrinsic apoptosis is initiated by assembly of death-inducing signaling complexes (DISCs) on the cytoplasmic tails of death receptors. DISCs are composed of oligomerized DR4/5, the adaptor protein FADD, initiator procaspases-8 and 10 (hitherto abbreviated pro-C8), caspase competitors c-FLIP long and short (cellular FLICE inhibitory protein or *cFLAR*), and several other molecules (Pennarun *et al*, 2010). DISC formation promotes the activation of pro-C8 (Kischkel *et al*, 1995; Martin *et al*, 1998) via dimerization and self-cleavage (Medema *et al*, 1997; Muzio *et al*, 1998; Chang *et al*, 2003). This process is antagonized by c-FLIP, which competes with pro-C8 for binding to FADD (Scaffidi *et al*, 1999). Once activated at the DISC, C8 cleaves effector procaspases-3 and 7, generating enzymes that process ICAD, digest the cellular proteome and genome, and kill the cells. However, caspases-3 and 7 are held in check in most cells by the inhibitor of apoptosis protein XIAP, which targets caspases for ubiquitin-mediated proteolysis (Deveraux *et al*, 1997). In epithelial and many other types of cells, death is triggered by mitochondrial outer membrane permeabilization (MOMP; Deng *et al*, 2002; Sun *et al*, 2002; Barnhart *et al*, 2003) and the consequent translocation of cytochrome c and SMAC into the cytosol (Goldstein *et al*, 2000), where SMAC binds to and inactivates XIAP, thereby relieving XIAP-mediated caspase-3/7 inhibition (Luo *et al*, 1998; Li *et al*, 2002; Riedl & Salvesen, 2007). MOMP is regulated in extrinsic apoptosis by C8-mediated cleavage of Bid, which generates a truncated tBid protein that translocates to mitochondria and activates the pore-forming Bcl-2 family members Bax and Bak (Eskes *et al*, 2000). MOMP occurs when levels of active Bax/Bak exceed sequestration by the anti-apoptotic proteins Bcl-2 and Bcl-XL. Following MOMP, sudden activation of effector caspases causes rapid cell death (Rehm *et al*, 2006; Albeck *et al*, 2008a).

Many studies have identified and characterized factors involved in sensitivity and resistance to TRAIL (Wagner *et al*, 2007; Chen *et al*, 2012; Passante *et al*, 2013). These studies have demonstrated a role for c-FLIP and Bcl-2 overexpression, DR4/5 modification and decoy receptors (which bind ligand but do not form DISCs), and other proteins as resistance factors in multiple cell types (Dimberg *et al*, 2013). In parallel, single-cell studies have shown that even cell lines classified as ‘sensitive’ exhibit fractional cell killing, whereby only a subset of cells undergoes apoptosis at saturating doses of TRAIL. Fractional killing is a stable property of homogenous cell populations: Recovering and replating cells that survive an initial challenge with TRAIL yields a new population of cells that, following several days of outgrowth, exhibit equivalent fractional cell death in response to TRAIL (Flusberg *et al*, 2013). We have shown that fractional effects arising from cell-to-cell variability also play a significant role in the dose–response properties of many other anti-cancer drugs (Fallahi-Sichani *et al*, 2013).

Following exposure of cells to TRAIL in the presence of cycloheximide, the time at which MOMP takes place is highly variable from one cell to the next: It can occur as early as 40 min or as late as 8–12 h after ligand exposure (Rehm *et al*, 2002; Albeck *et al*, 2008a; Hellwig *et al*, 2008). Lineage studies show that the time of death is highly correlated in newly born sister cells but that cells de-correlate rapidly and within 1–2 cell divisions are no more similar to each other than randomly selected members of the population. The stability of fractional killing through repeated rounds of death and recovery, and the phenomenon of ‘transient heritability’ in the time of cell death are inconsistent with a stable genetic difference between living and dying cells. Instead, cell-to-cell variability appears to arise from natural fluctuations in the concentrations and activities of pro- and anti-apoptotic proteins (Bhola & Simon, 2009; Rehm *et al*, 2009; Spencer *et al*, 2009) and the ability of these fluctuations to influence cellular biochemistry at a phenotypic level.

The similarity between fractional killing and variability in the time of cell death following TRAIL exposure suggests a fundamental connection between the two phenomena, but precisely what this is remains unknown. A limitation in single-cell studies of TRAIL described to date is that almost all have been performed in the presence of cycloheximide (Rehm *et al*, 2002; Albeck *et al*, 2008a; Spencer *et al*, 2009), which causes all TRAIL-treated cells to die, albeit at different times. The use of cycloheximide greatly simplifies the construction and validation of detailed, computational models of apoptosis because it minimizes the impact of protein synthesis, but it also precludes the identification of differences between cells that live and those that die. In this paper, we use live-cell imaging of cells exposed to TRAIL or therapeutic antibodies in the absence of cycloheximide to investigate the molecular basis of fractional killing. We find that a simple three-parameter model involving the rate and duration of C8 activation by the DISC is sufficient to discriminate between surviving and dying cells across a range of DR4/5 agonist types and concentrations, and that cell-to-cell variation in these parameters usually spans the fate boundary. Our model also quantifies how c-FLIP and Bcl-2 overexpressions promote TRAIL resistance and how the proteasome inhibitor bortezomib promotes TRAIL sensitivity. The model appears to be predictive in about half of cancer cell lines examined, with the other lines exhibiting forms of resistance external to our analysis. Our findings provide a new perspective on the relatively poor potency of once-promising but now discontinued therapeutic antibodies such as apomab and mapatumumab and suggest how new therapeutic molecules might be evaluated to ensure greater efficacy.

## Results

### A quantitative model of C8 activation dynamics in surviving and dying cells

We measured C8 activity prior to MOMP in single cells exposed to TRAIL and other DR4/5 agonists by imaging ICRP (Albeck *et al*, 2008a), a fluorescent reporter protein in which CFP and YFP are linked by a peptide derived from the natural initiator caspase substrate Bid; proteolysis of this linker changes the FRET ratio over time, FR(*t*). We typically imaged cells for a total of 22 h but focused on the first 10 h after TRAIL addition because most C8 activation or cell death events occurred within this time frame.

**Figure 1:**
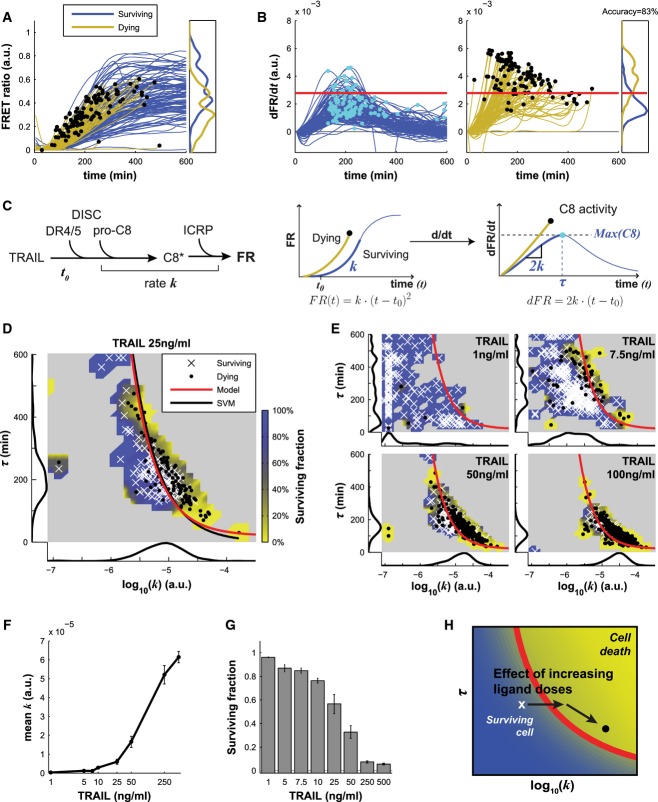
DISC dynamics predict cell fate after TRAIL treatments

We used cell morphology to score apoptosis. The scoring was validated using an IMS-RP reporter protein whose translocation from mitochondria to the cytosol functions as a live-cell measure of MOMP (Albeck *et al*, 2008a). Cell morphology and IMS-RP translocation were scored automatically using custom-developed image-processing routines, and the agreement between the two measures was 80 ± 2%. We therefore used a conservative threshold for scoring cell killing in the experiments described below (for details on the image-processing methodology, see Supplementary Materials and Methods; the custom image-processing routines, the complete set of single-cell trajectories, and example movies are also available for download at http://lincs.hms.harvard.edu/roux-molsystbiol-2015). When we adjusted the concentration of TRAIL so as to kill ∼50% of cells (this corresponded to 25 ng/ml or ∼0.4 nM), we observed that FR(*t*) trajectories varied from one cell to the next but that the trajectories were not obviously different in cells that ultimately died (Fig1A, yellow; black dots denote cell death) from those that survived (Fig1A, blue) except that survivors were still present at the end of the experiment. We have previously shown that cell-to-cell variability in FR(*t*) trajectories arises in clonal cell populations from fluctuation in the levels and activities of apoptotic regulators, including C8 (Spencer *et al*, 2009; Gaudet *et al*, 2012).

FRET ratio trajectories for ICRP-expressing and surviving HeLa cells (blue) or cells that died (yellow) following treatment with 25 ng/ml of TRAIL (ICRP cleavage reports on initiator caspase (C8) activity). Black dots denote cell death, at which point trajectories were truncated. All cells exhibited some C8 activation, and the right panel shows the distribution of maximal FRET ratio levels for ˜300 dying and living cells.

Derivative of the FRET ratio for surviving cells (blue, left panel) and cells committing to apoptosis (yellow, middle panel) for the same experiment as in (A). Cyan and black dots show the maximal value of the derivative (note that black dots also denote the time of cell death). The right panel shows the distribution of maximal values of the derivative for both populations. The red line indicates the optimal value θ_*T*25_ = 2.61 × 10^−3^ that separates the two populations with 83% accuracy.

Phenomenological model of caspase-8 activation and ICRP probe cleavage. FR stands for FRET ratio.

Caspase-8 activity landscape defined by the rate of C8 activation (*k*) and the duration of C8 activation phase (τ) for the same experiment as in (A). Surviving cells are shown as white crosses; dead cells as black dots. The red line is the fate boundary calculated with EQ3 θ_*T*_ = 2.63 × 10^−3^ (see text); the black line is a linear support vector machine (SVM) classifier. The background color is based on the fate of cells (blue for surviving and yellow for dying). Marginal distributions are plotted along each axis.

Same landscapes as in (D) for different doses of TRAIL.

Geometric mean of *k* for different doses of TRAIL. Data are represented as mean ± SEM.

Fraction of surviving cells for different doses of TRAIL. Data are represented as mean ± SEM.

Graphical representation of the effect of ligand dose on the caspase-8 activity landscape: For increasing low doses (1–10 ng/ml), cells are pushed to the right toward the fate boundary. At higher doses (greater than 25 ng/ml), increasing doses push cells toward the lower right corner along the fate boundary.

Source data are available online for this figure.

When we computed the time derivative of FR(*t*) (i.e., dFR/d*t*), we could immediately distinguish between dying and surviving cells: In cells that died, dFR/d*t* trajectories rose until the onset of apoptosis (Fig1B, yellow), whereas in surviving cells, dFR/d*t* trajectories remained lower on average and fell back to pre-treatment levels by 4–8 h (Fig1B, blue). Western blotting demonstrated that the fall in ICRP cleavage rates was not simply a consequence of exhaustion of unprocessed reporter protein (Supplementary Fig S1A). By recording the maximum value of dFR/d*t* for each cell, we could visually identify a cutoff that separated dying from surviving cells (the red line in Fig1B). By minimizing the live/dead classification error, we computed the cutoff to be dFR*/*d*t *= θ_*T*25_ = 2.61 ± 0.16 × 10^−3^ (a.u.) with an accuracy of 83 ± 3% (see Supplementary Materials and Methods; the subscript ‘T25’ denotes the value of the cutoff computed in parental cells exposed to 25 ng/ml TRAIL). The biological significance of this observation is that the derivative of FR(*t*) represents the activity of C8 under the assumption that cleaved ICRP reporter is relatively stable, as previously shown (Albeck *et al*, 2008a). Thus, the threshold defined by θ_*T*25_ effectively represents the minimum instantaneous C8 activity necessary for cells to die.

In this analysis, we were careful to include only C8 trajectories in pre-MOMP cells, thereby avoiding complications arising from cleavage of ICRP by effector caspases-3/7 (see Materials and Methods for details). Analysis of cells overexpressing Bcl-2 or Bcl-XL, which blocks MOMP and consequent C3 activation, confirmed that our calculation of θ was not influenced by effector caspase activity (Supplementary Fig S1B and see below). Moreover, the accuracy of θ as a classifier was as good as what might be expected from first principles: Random fluctuations in the activities and levels of proteins functioning downstream of the DISC (e.g., regulators of MOMP or effector caspase) have previously been shown to contribute ∼20% to variability in the timing of cell death (Gaudet *et al*, 2012).

To characterize factors controlling C8 dynamics, we developed a simplified two-step model of the underlying biochemical reactions in which binding of ligand to DR4/5 activates C8 at the DISC and C8 then cleaves ICRP. Under these assumptions, FR(*t*) depends quadratically on time: FR (*t*) = *k* ·(*t *− *t*_0_)^2^ (EQ1), and C8 activity is described by 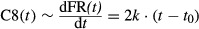 (EQ2), where *k* represents the rate of C8 activation at the DISC and *t*_0_ the lag between addition of ligand (at *t *= 0) and first detectable ICRP cleavage. This model captures the period immediately after ligand exposure in which C8 activity is rising and is valid only until dFR/d*t* reaches its maximum at time *t = *τ (i.e., Max(C8); see Fig1C); after this, the trajectory is strongly influenced by biochemical processes not considered in the model. These are likely to include protein degradation, negative feedback on DISC activity, or, in the case of cells that have undergone MOMP, the activity of effector caspase. To estimate values for *k* and *t*_0_, we fitted EQ1 to the ICRP trajectory of 200–300 cells exposed to 25 ng/ml of TRAIL (Fig1C) and obtained a good fit (*r*^2^ > 0.9) for 92 ± 1% of the trajectories. Parallel analysis of dFR*/*d*t* trajectories from cells overexpressing Bcl-2 or Bcl-XL (Supplementary Fig S1C) confirmed that our estimates for *k* were not influenced by effector caspase activity.

In a two-dimensional landscape of τ and *k* computed from single-cell trajectories, θ corresponds to a line that separates cells by fate, with surviving cells falling to the left of the fate boundary (low *k* and/or short τ, in blue) and dead cells to the right of the boundary (higher *k* and/or longer τ, in yellow; Fig1D). This arises because Max(C8) occurs when *t* = τ and by EQ2, Max(C8) = 2*k* (τ* − t*_0_). As described above, cells die when Max(C8) > θ; thus, the dividing line between dying and surviving cells is τ *= *θ*/*2*k* ; *t*_0_ (EQ3) with θ_*T*25_ = 2.61 × 10^−3^ and *t*_0_ = 20 min (the average value of the lag time across all fitted trajectories). This dividing line (i.e., cell fate boundary) is shown with a red line in Fig1B and plotted in the landscape of *k* and τ in Fig1D. The accuracy of the boundary (72 ± 4% at 25 ng/ml of TRAIL) was not significantly different from that of a purely data-driven classifier constructed using a support vector machine (72 ± 2%; Fig1D; Supplementary Fig S1D). The existence of a cell fate boundary in our data highlights the fundamental differences between the current work and previous research (Spencer *et al*, 2009) in which the exposure of cells to TRAIL in the presence of cycloheximide resulted in the death of all cells even at low doses of TRAIL. We conclude that the rate of C8 activation (*k*) and the duration of this initial activation (τ) play a dominant role in determining whether cells undergo TRAIL-induced apoptosis. A lower rate of activation for a longer time will not suffice, even if it achieves the same total level of cleaved ICRP substrate.

### A cell fate boundary is set by the rate and duration of C8 activation

For the boundary in the landscape of τ and *k* to be biologically meaningful as a threshold, it should minimally be constant across agonist dose and class. We observed that the value of *k* (the geometric mean across cells) increased ∼140-fold as the dose of TRAIL increased from 1 to 500 ng/ml (as shown by the marginal distributions plotted below the landscapes in Fig1E and F), and the fraction of dying cells rose from 4 to 92% (Fig1G). This caused cells to ‘move’ rightward in the landscape of *k* and τ (Fig1H), but the cell fate boundary (computed as θ_*T*25_) remained 77 to 97% accurate depending on dose (Supplementary Fig S1E). Moreover, when we recomputed θ using data from a wide range of TRAIL doses (10–500 ng/ml), we recovered a value θ_*T*_ = 2.63 × 10^−3^ that was indistinguishable from θ_*T*25_. Thus, θ is a dose-independent parameter for a particular cell line (parental HeLa cells in this case). Inspection of marginal distributions showed that *k* and τ co-varied to some extent across a range of TRAIL concentrations. We ascribe the > 100-fold increase in *k* to changes in DISC activity; concomitant changes in the distribution of τ arise simply because τ cannot be longer than the time between ligand addition and death (by definition; as shown by the marginal distributions to the left of the landscapes in Fig1E).

**Figure 2:**
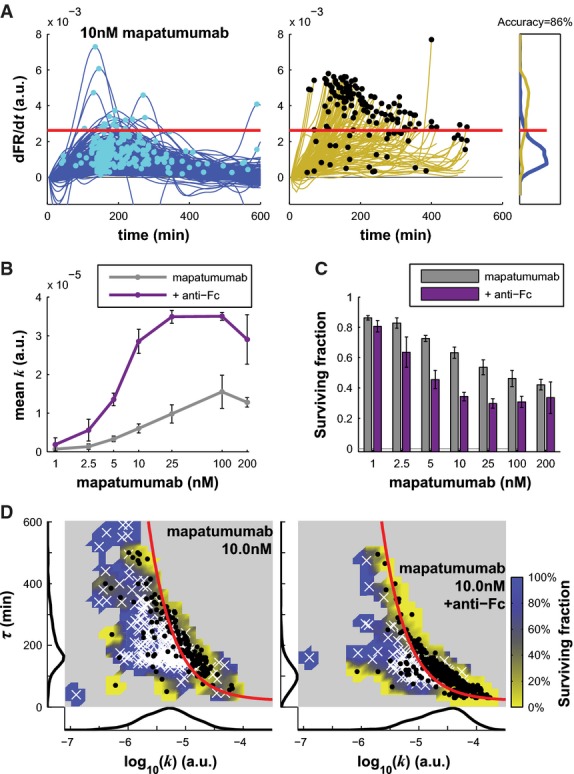
Receptor clustering increases the rate of C8 activation and cell killing

When we imaged ICRP in cells exposed to mapatumumab, a therapeutic antibody that functions as a DR4 agonist (Pukac *et al*, 2005), we observed the same dynamics as in TRAIL-treated cells: dFR/d*t* rose monotonically in dying cells, whereas in surviving cells, it peaked at *t* = 2–4 h and then fell (Fig2A). The θ_*T*_ threshold was 70–90% accurate in predicting apoptosis induced by mapatumumab across a 1–200 nM dose range (denoted by the red line in Fig2A; Supplementary Fig S2A). One striking difference between mapatumumab and TRAIL is that at saturating doses mapatumumab elicited a mean C8 activation rate that was fourfold lower (*k* ∼1.3 versus 5 × 10^−5^; Figs1F and 2B). This cannot be a simple matter of affinity because mapatumumab was tested at saturating concentrations with respect to cell killing (200 nM), as evidenced by the fact that *k* and fractional cell killing decreased at higher dose. This ‘squelching’ effect was observed for multiple agonist antibodies and is not a consequence of measurement error (Supplementary Fig S4B).

A Derivative of FRET ratio for surviving HeLa cells (blue, left panel) and cells committing to apoptosis (yellow, middle panel) following treatment with 10 nM of mapatumumab. Cyan and black dots show the maximal value of the derivative (note that black dots also indicate cell death). The right panel shows the distribution of maximal values of the derivative for both populations. The red line indicates the value θ_*T*_ = 2.63 × 10^−3^ that separates the two populations with 83% accuracy.

B, C Geometric mean of *k* (B) or fraction of surviving cells (C) for different doses of mapatumumab alone (gray) or with clustering agent (purple). Data are represented as mean ± SEM.

D Caspase-8 activity landscapes following treatment of cells with 10 nM of mapatumumab alone (left panel) or with the clustering agent anti-Fc (right panel). Surviving cells are white crosses; dead cells are black dots. The red line is the fate boundary calculated with EQ3. The background color is based on the fate of cells (blue for surviving and yellow for dying). Marginal distributions are plotted along each axis.

Source data are available online for this figure.

It has previously been shown that the potency of anti-DR4/5 antibodies can be increased by cross-linking them with anti-Fc antibodies (Adams *et al*, 2008). This is thought to arise because TRAIL is a trimeric agonist, whereas antibodies are dimeric and addition of anti-Fc creates tetramers and higher order assemblies. Imaging of cells treated with 10 nM mapatumumab plus 10 nM anti-Fc increased *k* ∼fivefold (Fig2B) and cell killing ∼twofold (Fig2C). This corresponds to a movement to the right in the landscape of *k* and τ (compare the left and right panels in Fig2D), causing a greater number of cells to cross the cell fate boundary defined by θ_*T*_ (Supplementary Fig S2B). This makes sense based on current understanding of C8 activation by clustered receptors (Muzio *et al*, 1998; Majkut *et al*, 2014): Multivalent mapatumumab increases pro-C8 recruitment to the DISC (even at saturating concentrations) and thus C8 activity. Nevertheless, clustered mapatumumab remained at least twofold less active than recombinant TRAIL at saturation, as measured by C8 activity.

From these data, we conclude that θ constitutes a threshold in the biological sense: It is constant for cells exposed to different DR4/5 agonists over a range of concentrations and cells overexpressing resistance genes (as shown below). Apoptosis is also known to be regulated at the level of MOMP by a threshold that involves a balance between pro- and anti-apoptotic Bcl-2 proteins (Chipuk *et al*, 2010; Chi *et al*, 2014). MOMP takes place only when the levels of pro-apoptotic proteins exceed the level of negative regulators such as Bcl-2 and Bcl-XL (in receptor-mediated apoptosis, the pro-apoptotic signal corresponds to the amount of cleaved and active tBid plus the amount of Bax and Bak already activated by tBid). It is therefore striking that the life/death threshold at the level of DISC involves the rate of C8 activation, which is proportional to the rate of tBid production, rather than the final level of cleaved ICRP substrate, which is proportional to the total level of Bid processed into tBid (compare Fig1A and B). In principle, initial and peak C8 activity might be predictive of TRAIL-mediated apoptosis simply because TRAIL is a short-lived ligand; however, the initial rate is also predictive of apoptosis induced by mapatumumab, which is stable in solution.

### C8 trajectories and cell killing are controlled by proteasome-mediated caspase degradation

**Figure 3:**
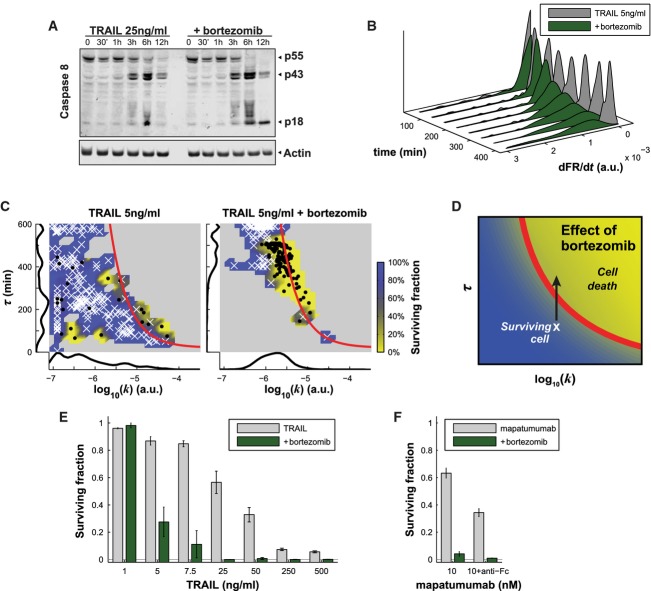
Bortezomib induces TRAIL-dependent apoptosis by increasing the duration of caspase-8 activation

C8 is a short-lived protein subject to ubiquitin-dependent degradation by the proteasome (Thorpe *et al*, 2008; Gonzalvez *et al*, 2012). To investigate the role of protein degradation in C8 activity at *t* > τ, we used the selective proteasome inhibitor bortezomib (the anti-cancer drug Velcade®) at a dose (100 nM) that did not itself result in detectable C8 activation and did not induce measurable apoptosis within 24 h (Supplementary Fig S3A). We observed that the catalytically active form of C8 (C8-p18) accumulated for up to ∼12 h in cells exposed to 100 nM bortezomib in combination with 25 ng/ml TRAIL (Fig3A, Western blot measurements were performed in cells overexpressing Bcl-2 to block effector caspase activation). In contrast, in cells treated with TRAIL alone, C8-p18 levels peaked at 6 h and fell to background levels by 12 h (Fig3A). The great majority of dFR/d*t* trajectories in cells treated with bortezomib and TRAIL were monotonic without evidence of a subsequent fall in *k* (Supplementary Fig S3A, upper panels). As a consequence, the duration of C8 activation increased (compare the gray and green distributions in Fig3B), and this manifested itself in the landscape of *k* and τ as a shift upward and slightly to the right (Fig3C and D). We conclude that the value of τ is sensitive to proteasome inhibition and that the fall in dFR*/*d*t* trajectories is caused in large part by degradation of C8.

Western blot analysis of caspase-8 levels after TRAIL treatment alone (left) or with bortezomib (right) at different times after exposure of HeLa ICRP Bcl-2-overexpressing cells with TRAIL.

Distribution of caspase-8 activity at different time points for cells treated with TRAIL alone (gray, same experiment as in Fig1) or TRAIL with bortezomib (green).

Caspase-8 activity landscape following treatment with 5 ng/ml of TRAIL alone (left panel) and with 100 nM of bortezomib (right panel). Surviving cells are represented by white crosses and dead cells by black dots. The red line is the fate boundary calculated with EQ3. The background color is based on the fate of cells (blue for surviving and yellow for dying). Marginal distributions are plotted along each axis.

Graphical representation of the effect of bortezomib on the caspase-8 activity landscape: Bortezomib increases the duration of caspase-8 activity, pushing cells up in the landscape.

Surviving cell fractions following exposure to different doses of TRAIL without (gray) and with bortezomib (green). Data are represented as mean ± SEM.

Surviving cell fractions following exposure to 10 nM of mapatumumab (left) or 10 nM mapatumumab with anti-Fc (right), either without (gray) or with bortezomib (green). Data are represented as mean ± SEM.

Source data are available online for this figure.

To better determine the phenotypic consequences of exposing cells to TRAIL in the presence of bortezomib, we used a concentration of TRAIL (5 ng/ml) at which cell killing was low. Co-treatment with bortezomib increased killing sixfold (from 12 ± 2% to 72 ± 11%, Fig3E) concomitant with a 1.5-fold change in the mean value of τ (Supplementary Fig S3B, left panel). This relatively small change in τ caused a large increase in cell killing because many cells lay just below the cell fate boundary. Bortezomib had the same effect on cells co-treated with mapatumumab, extending the duration of C8 activation (Supplementary Fig S3B, right panel) and increasing cell killing (Fig3F). However, θ_*T*_ remained > 95% predictive for apoptosis mediated by bortezomib in combination with either TRAIL or mapatumumab (Supplementary Fig S3A). Thus, even though bortezomib is likely to alter the levels or activities of multiple apoptosis regulators and myriad other cellular proteins (e.g., it changes DR5 abundance at *t* = 6 h; Supplementary Fig S3C), changes in C8 activation dynamics (τ primarily) are sufficient to fully account for the observed effects of bortezomib on cell killing by DR4/5 agonists. This finding illustrates a useful property of phenomenological biochemical models: They can establish which mechanisms are sufficient to explain a quantitative phenotype.

### Predicting and confirming three-agent effects

**Figure 4:**
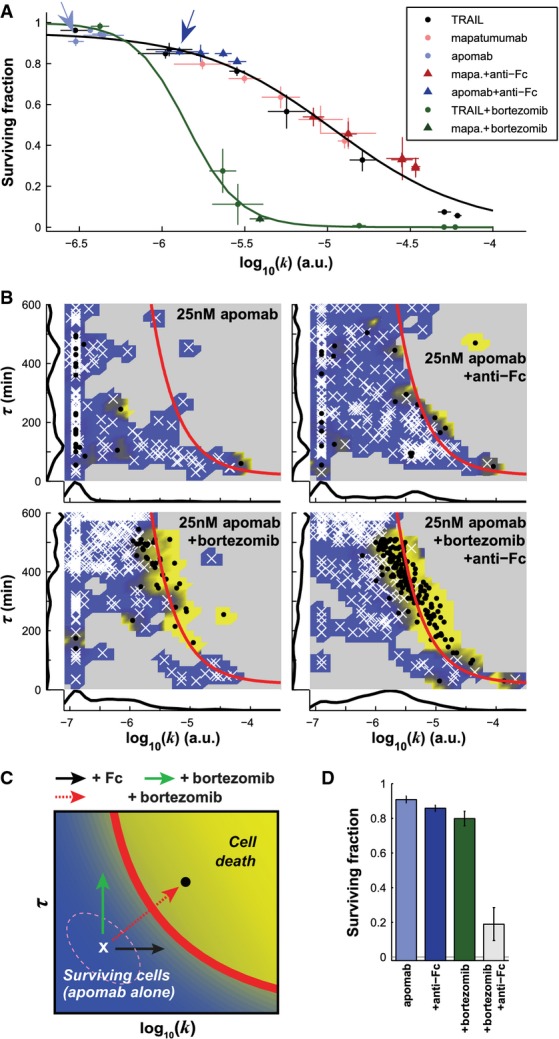
Bortezomib and the clustering antibody anti-Fc recover apomab potency

When we studied the induction of apoptosis by the DR5 agonist apomab, a therapeutic antibody whose development was discontinued following failure in Phase II clinical trials, we observed that even at saturating doses (≥ 100 nM), only 6 ± 2% of cells died (Supplementary Fig S4A). To determine why such a high-affinity antibody (*K*_D_ ∼1  nM (Adams *et al*, 2008)) had such low potency, we plotted the fraction of apoptotic cells against *k* for multiple doses of TRAIL, mapatumumab, and apomab with and without anti-Fc cross-linking. All values fell on a single logarithmic curve (Fig4A, black line, *r*^2^ = 0.94 for all replicates), consistent with our hypothesis that the rate of C8 activation bears a constant relationship to the fraction of cells killed. Data for cells treated with bortezomib fell on a different curve (Fig4A, green line) reflecting the fundamentally different dynamics of C8 activation when the proteasome is inhibited. Remarkably, 100 nM apomab was similar in activity (*k* ∼5 × 10^−7^ and 4–7% cell killing) to 1 ng/ml (0.025 nM) TRAIL, implying a 4,000-fold difference in potency in HeLa cells. Clustering apomab with anti-Fc antibodies increased *k* ∼fivefold (Supplementary Fig S4B), similar to the increase observed when mapatumumab was clustered (Fig2B), but in the landscape of *k* and τ*,* this was insufficient to kill more than a few cells because the duration of C8 activation remained too short (Fig4B, upper panels). From previous data on TRAIL and mapatumumab, we predicted that addition of 100 nM bortezomib would increase τ ∼twofold, marginally increasing cell killing; experiments confirmed this prediction (Fig4B lower left panel; Fig4C). However, we also predicted that, by increasing both *k* and τ, a combination of bortezomib and anti-Fc would be sufficient to push a significant fraction of apomab-treated cells over the life–death boundary and induce apoptosis (Fig4C). This is precisely what was observed, with up to 80% of cells dying in the presence of the three-way combination of 100 nM bortezomib, 25 nM apomab, and 25 nM anti-Fc (Fig4B lower panels; Fig4D). Thus, a simple 2-step model of DISC activation as interpreted in the landscape of *k* and τ can predict the activities of diverse DR4/5 agonists across a range of doses, in the presence and absence of clustering agents and proteasome inhibitors. In the specific case of apomab, we conclude that the antibody alone was insufficiently potent with respect to both rate and duration of C8 activation (Fig4C). HeLa cells are not unique in this regard: In nine of nine TRAIL-sensitive cancer cell lines tested, apomab induced little or no killing on its own (typically below 10% at 10 nM), and when clustered with anti-Fc, it induced a partial response (≥ 40% cell killing) in only five of nine cell lines (Supplementary Fig S4C). The relatively low potency of apomab provides a potential explanation for the failure of this molecule as a drug.

Surviving cell fraction as a function of the mean of the value of log_10_(*k*) for all cells following exposure to different doses of TRAIL (black), apomab (blue), or mapatumumab (red), without (light color dots) or with clustering antibody anti-Fc (darker color triangles), as well as after treatments with TRAIL or mapatumumab combined with bortezomib (green dots and triangles, respectively). The black and green lines are logistic curves fitted to all conditions lacking (black) or including bortezomib (green). Arrows show the data points for 25 nM of apomab without (light blue) and with anti-Fc (dark blue). Data are represented as mean ± SEM.

Caspase-8 activity landscape following exposure of HeLa cells to 25 nM of apomab alone (left panels) or with clustering agent anti-Fc (right panels), without (top panels) or with bortezomib (lower panels). Surviving cells are represented as white crosses; dead cells are black dots. The red line is the fate boundary calculated with EQ3. The background color is based on the fate of cells (blue for surviving and yellow for dying). Marginal distributions are plotted along each axis.

Graphical representation of the effects of apomab and co-drugging on the caspase-8 activity landscape: bortezomib increases the value of τ*,* while the clustering antibody (anti-Fc) increases *k*, but only the combination of bortezomib and anti-Fc can push the cells across the fate boundary.

Fraction of surviving cells for the conditions described in (B). Data are represented as mean ± SEM.

Source data are available online for this figure.

### Differential effects of FLIP isoforms on C8 dynamics and cell killing

**Figure 5:**
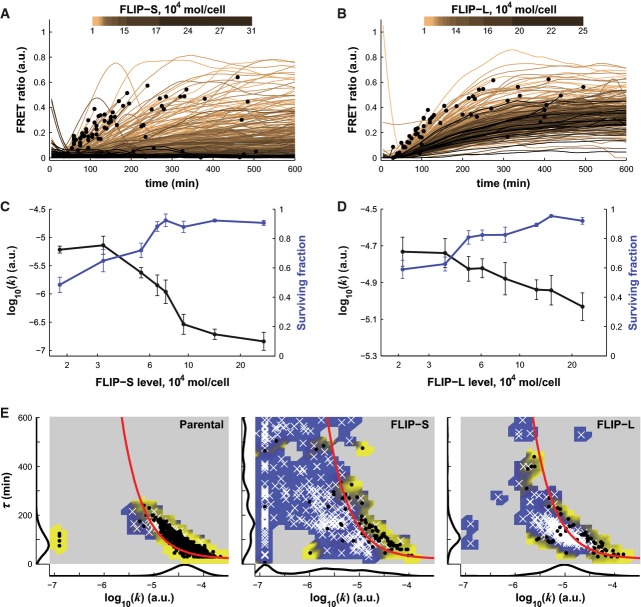
Quantitative measure of FLIP-S- and FLIP-L-dependent inhibitions of caspase-8 dynamics

FLIP is one of the proteins most frequently implicated in resistance to TRAIL (Shirley & Micheau, 2013). It is homologous to C8, has two DISC-binding death effector domains at its N-terminus, and comprises two short 25–27 kD isoforms, FLIP-R and FLIP-S, and one long 55 kD isoform, FLIP-L. FLIP-S and FLIP-R do not have caspase domains, and they compete with C8 for recruitment to the DISC, thereby functioning as classic dominant-negative proteins. FLIP-L has a catalytically inactive caspase domain but can activate pro-C8 as part of a heterocomplex with native caspase; it therefore functions as a C8 inhibitor under most conditions but as an activator under conditions of ‘high’ receptor activation (Fricker *et al*, 2010). To study C8 dynamics in the presence of resistance factors, we generated HeLa cell lines expressing FLIP-L or FLIP-S tagged with mCherry (HeLa cells possess naturally low levels of FLIP-L/S). Stably expressing cells were sorted into four pools based on mCherry fluorescence and absolute FLIP levels estimated by semi-quantitative Western blotting (Supplementary Fig S5A and B; see Materials and Methods for details). Over a 20-fold range of expression, we observed that FLIP-L or FLIP-S levels correlated negatively with *k*, as expected for apoptosis inhibitors (Spearman's ρ = −0.57 ± 0.04, *P* < 10^−11^ and ρ = −0.37 ± 0.01, *P* < 10^−4^, respectively, Fig5A and B; Supplementary Fig S5C). HeLa cells contain ∼1.5 × 10^5^ pro-C8 molecules per cell, and full inhibition of ICRP processing (*k* ≤ 10^−7^) occurred at a FLIP-S:pro-C8 ratio of ∼2:3 which is consistent with the idea that FLIP-S has a similar affinity for the DISC as C8 (Scaffidi *et al*, 1999; Majkut *et al*, 2014). Half-maximal inhibition of C8 (*k* < ∼10^−6^) was observed at ∼5 × 10^4^ FLIP-S molecules per cell (∼1:3 FLIP-S:pro-C8), and this was sufficient to block cell death almost completely (< 5% cell killing; Fig5C). Thus, the potency of FLIP-S as an apoptosis inhibitor is enhanced by the nonlinear relationship between *k* and the induction of apoptosis, and is not simply due to the biochemistry of FLIP–DISC interaction.

A, B FRET ratio trajectories for HeLa cells expressing different levels of FLIP-S-mCherry (A) or FLIP-L-mCherry (B) with protein level encoded by the intensity of the color (as judged by mCherry intensity; see Materials and Methods for details) in cells treated with 250 ng/ml of TRAIL. Black dots show cell death; trajectories are truncated subsequently.

C, D Relation between FLIP-S (C) or FLIP-L (D) level plotted on the *x*-axis and the rate of caspase-8 activation (black line), or the surviving fraction (blue line). Cells are binned by FLIP levels. Data are represented as mean ± SEM.

E Caspase-8 activity landscape for parental HeLa cells, FLIP-S-overexpressing cells (middle, same experiment as in A), and FLIP-L-overexpressing cells (right, same experiment as in B). All cells were treated with 250 ng/ml of TRAIL. Surviving cells are denoted with white crosses and dead cells with black dots. The red line is the fate boundary calculated with EQ3. The background color is based on the fate of cells (blue for surviving and yellow for dying). Marginal distributions are plotted along each axis.

Source data are available online for this figure.

The ability of FLIP-L overexpression to block cell death over a range of protein concentrations was similar to that of FLIP-S (on a molar basis, as illustrated by the blue lines in Fig5C and D), but FLIP-L had a significantly smaller effect than FLIP-S on the value of *k* (black lines). This implies that FLIP-L controls cell death in an additional way. If we select TRAIL doses such that FLIP-L-overexpressing cells and wild-type cells have similar mean values of *k* (Supplementary Fig S5D, left panel), we find that τ is substantially lower in the presence of FLIP-L (Supplementary Fig S5D, right panel). FLIP-L is known to activate NF-κB and a variety of other complex cellular processes (Kataoka & Tschopp, 2004; Golks *et al*, 2006; Neumann *et al*, 2010), but our simple model showed that the reduction in *k* and τ caused by FLIP-L overexpression is sufficient to explain the observed differences in cell killing between parental and FLIP-L-overexpressing cells (θ_*T*_ is 91 ± 1% predictive; Supplementary Fig S5E). Based on the literature, a probable molecular explanation is that FLIP-L heterodimerizes with C8 to form active caspase complexes with half-lives shorter than that of C8:C8 complexes (FLIP-S does not form active heterodimers and acts as a simple competitive inhibitor). In the landscape of *k* and τ, the net effect of FLIP expression is for FLIP-S to shift cells strongly to the upper left and for FLIP-L straight to the left. In both cases, cells no longer lie in the region of the landscape associated with apoptosis (Fig5E).

**Figure 6:**
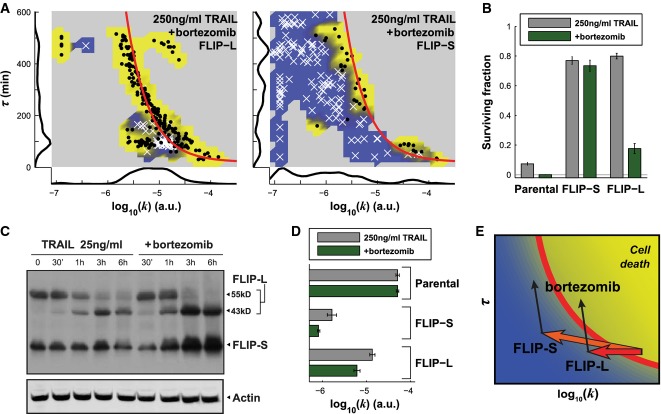
Bortezomib overcomes FLIP-L- but not FLIP-S-induced resistance

Because it blocks proteasome activity, we predicted that bortezomib should significantly increase τ in FLIP-S- and FLIP-L-overexpressing cells. Experiments confirmed this prediction (Fig6A, see the marginal distribution on the left), but the phenotypic consequences were different for the two FLIP isoforms. For FLIP-L-overexpressing cells, adding 100 nM bortezomib (which did not by itself cause apoptosis) increased cell killing from 20 to 80% (at 250 ng/ml TRAIL). However, exposure of FLIP-S-overexpressing cells to 250 ng/ml TRAIL resulted in very low levels of cell killing (< 25%) in the presence and absence of bortezomib (Fig6B). To determine why bortezomib sensitizes FLIP-L but not FLIP-S-overexpressing cells to TRAIL, we measured FLIP protein levels by Western blotting. We found that both FLIP-L and FLIP-S isoforms were more abundant in bortezomib-treated than untreated cells (2.5-fold and 3.5-fold, respectively; Fig6C), reflecting the fact that, like C8, FLIP proteins are degraded by ubiquitin-dependent proteolysis (Poukkula *et al*, 2005; Chang *et al*, 2006; Laussmann *et al*, 2012). The reduction in *k* that results from stabilization of FLIP (an anti-apoptotic change) counteracts the increase in τ (a pro-apoptotic change) caused by stabilization of C8 (Fig6D). By visualizing these changes in the landscape of *k* and τ*,* we can see that, on balance, bortezomib is sufficient to push FLIP-L-overexpressing cells above the fate boundary but that that is not true of FLIP-S-overexpressing cells. Thus, relatively subtle differences in the chemistry of C8, FLIP-L, and FLIP-S have large phenotypic consequences (Fig6E).

Caspase-8 activity landscape for FLIP-L-overexpressing cells (left) and FLIP-S-overexpressing cells (right) treated with 250 ng/ml of TRAIL and bortezomib. Surviving cells are denoted with white crosses and dead cells with black dots. The red line is the fate boundary calculated with EQ3. The background color is based on the fate of cells (blue for surviving and yellow for dying). Marginal distributions are plotted along each axis.

Surviving fraction of cells for parental HeLa cells or for FLIP-S-overexpressing cells or FLIP-L-overexpressing cells treated with 250 ng/ml of TRAIL, without (gray) or with bortezomib (green). Data are represented as mean ± SEM.

Western blot analysis of FLIP levels after treatment of HeLa cells with 25 ng/ml of TRAIL alone (left) or with bortezomib (right) for different times.

Mean of log_10_(*k*) for the parental lines, the FLIP-S- and the FLIP-L-overexpressing cells treated with 250 ng/ml of TRAIL, without (gray) or with bortezomib (green). Data are represented as mean ± SEM.

Graphical representation of the effects of FLIP overexpression and bortezomib on the caspase-8 activity landscape in TRAIL-treated cells: FLIP-L decreases *k*, but the remaining C8 activity is strong enough for bortezomib to push cells upward across the fate boundary. FLIP-S has a greater effect on *k* and bortezomib decreases *k* even more, preventing cells to cross the fate boundary although the activity is sustained over a longer period.

Source data are available online for this figure.

### Modulating the position of the cell fate boundary

**Figure 7:**
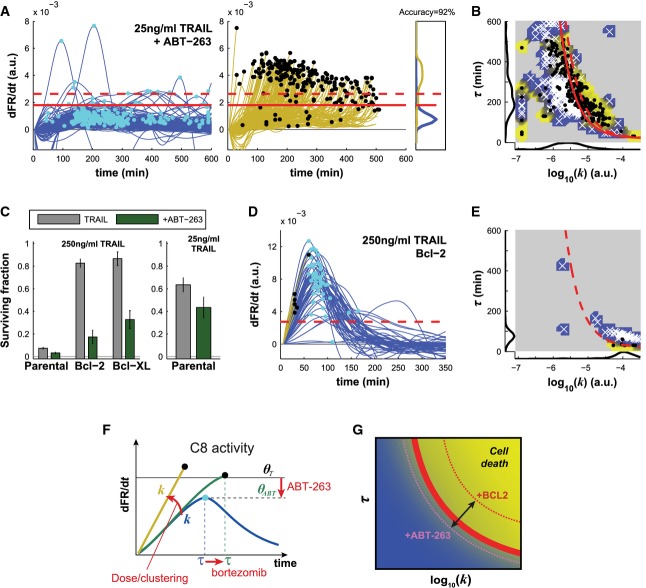
Inhibition and overexpression of Bcl-2 proteins shift the position of the cell fate threshold θ

The best-characterized threshold in the regulation of apoptosis involves the pro- and anti-apoptotic Bcl-2 proteins. Their ratio establishes the level of activating Bcl-2 family proteins (e.g., tBid) required for MOMP and subsequent cell death. To determine the relationship between θ and the threshold for MOMP, we measured FR(*t*) dynamics in TRAIL-treated control cells or in cells overexpressing Bcl-2 or Bcl-XL, in the presence or absence of the Bcl-2 inhibitor ABT-263 (Navitoclax). When parental cells were exposed to 25 ng/ml TRAIL in combination with 10 μM ABT-263 (a dose of drug that did not itself induce detectable cell death), C8 dynamics were not altered (Fig7A and B), and *k* or τ were not significantly different (Supplementary Fig S6A and B), but the fraction of dying cells increased from 45 to 60% (Fig7C, right panel). Based on EQ3, increased cell death can be explained by a decrease of θ, and we found that θ in ABT-263-treated cells (θ_ABT_) was 1.6-fold lower than θ_*T*_ (Fig7A and B). Most cells overexpressing either Bcl-2 or Bcl-XL survived TRAIL concentrations of 250 ng/ml (the fraction of apoptotic cells was reduced from 92% in parental cells to less than 20% in Bcl-2-/Bcl-XL-overexpressing cells) although maximal C8 activity was much higher than what would be needed to cross θ_*T*_ (Fig7D and E; Supplementary Fig S6C). However, the addition of 10 μM ABT-263 increased fractional killing of Bcl-2-/Bcl-XL-overexpressing cells by TRAIL to 70% (Fig7C, left panel). Because the level of Bcl-2 or Bcl-XL was heterogeneous across cells in this experiment, we could also confirm the prediction that apoptosis took place selectively in cells with a lower level of Bcl-2 or Bcl-XL (*P* = 0.01 ± 0.01, Wilcoxon rank sum test). We conclude that, whereas changing the dose or oligomeric state of DR4/5 agonists or co-drugging parental or FLIP-overexpressing cells with bortezomib changes *k* and τ in the context of a constant threshold value for θ (Fig7F), changing the levels or activity of Bcl-2 proteins changes the position of this threshold (Fig7G). In this way, θ and the threshold for MOMP interact to control the fraction of cells that die in response to a given level of DR4/5 agonism.

Derivative of FRET ratio for surviving cells (blue, left panel) and cells committing to apoptosis (yellow, middle panel) in cells co-treated with 25 ng/ml of TRAIL and ABT-263. Cyan and black dots show the maximal value of the derivative (note that black dots also indicate cell death). Right panel shows the distribution of the maximal value of the derivative for both populations. The plain red line indicates the optimal value of θ (θ_*ABT*_) that separates the two populations with 91% accuracy; the value is 19% lower than the value of θ_*T*_ computed for cells treated with TRAIL alone (dashed red line).

Caspase-8 activity landscape for parental HeLa cells treated with 25 ng/ml of TRAIL and ABT-263 (same experiment as in A). Surviving cells are white crosses; dead cells are black dots. The dashed red line is the fate boundary calculated with EQ3 of the parental HeLa cell line. The background color is based on the fate of the cells (blue for surviving and yellow for dying). Marginal distributions are plotted along each axis.

Surviving fractions for the parental lines (HeLa ICRP cells), the Bcl-2- and the Bcl-XL-overexpressing cells treated with 250 ng/ml (left) or 25 ng/ml of TRAIL (right) without or with ABT-263 (gray and green, respectively). Data are represented as mean ± SEM.

Derivative of FRET ratio for the Bcl-2-overexpressing cells treated with 250 ng/ml of TRAIL. The dashed red line indicates θ as defined for the parental population. Surviving cells are in blue and dying cells in yellow. Cyan and black dots show the maximal values of the derivative (note that black dots also indicate cell death).

Caspase-8 activity landscape for the Bcl-2-overexpressing cells treated with 250 ng/ml of TRAIL (same experiment as in D). Surviving cells are white crosses; dead cells are black dots. The dashed red line is the fate boundary calculated with EQ3 of the parental HeLa cell line. The background color is based on the fate of the cells (blue for surviving and yellow for dying). Marginal distributions are plotted along each axis.

Graphical representation of the effects of ligand dose, oligomeric state of DR4/5 agonists, and co-drugging with bortezomib or ABT-263 on dFR*/*d*t* and the threshold θ.

Graphical representation of the effects of Bcl-2/Bcl-XL overexpression and ABT-263 co-drugging on the caspase-8 activity landscape following TRAIL treatment. ABT-263 has the effect of reducing the fate boundary, whereas overexpression of Bcl-2 or Bcl-XL is interpreted as pushing the fate boundary toward the upper right.

Source data are available online for this figure.

### Caspase-8 activation in other cell types

**Figure 8:**
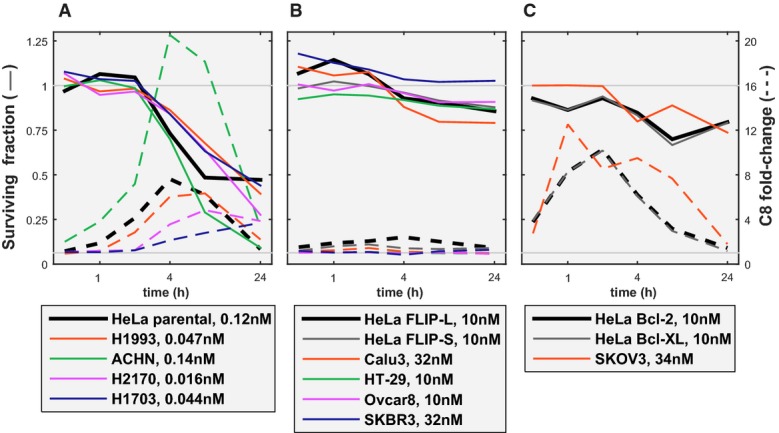
TRAIL-induced cell death and C8 activation measured in other cancer cell lines For each cell line, the dose of TRAIL shown in the legend is, for sensitive cell lines, the dose that resulted in ≥ 50% killing or, for resistant cell lines, the highest dose tested. The surviving cell fraction (plain line, left axis) and the C8 activity fold change (dashed line, right axis) are plotted as a function of time. These data were obtained at fixed points in time using fluorescent dyes as opposed to continuously in time using a genetically encoded reporter protein, as was done in HeLa cells for the previous figures. The measured value for C8 activity becomes progressively less accurate as cells begin to die, because effector caspases also cleave the dyes. The left panel shows cell lines that respond similarly to parental HeLa cells; the middle panel, the cell lines that respond similarly to FLIP-overexpressing HeLa cells; and the right panel, the cell lines that respond similarly to our Bcl-2-/XL-overexpressing HeLa cells.

To investigate how the fates of other cell types are regulated by TRAIL, we analyzed nine additional cell lines (including breast, renal, lung, ovarian, and colorectal cells) having a range of sensitivities to TRAIL. A luminescent assay was used to measure C8 activity in fixed cells at discrete times after the addition of TRAIL or agonist antibodies. This approach does not have the single-cell resolution of live-cell imaging and cannot cleanly distinguish between initiator and effector caspase activity, but it has the benefit that genetic manipulation of multiple cell lines is not necessary. In four cell lines (Fig8A), we observed that sub-nanomolar doses of TRAIL elicited rapid C8-substrate cleavage and > 50% cell death; these cell lines phenocopy parental HeLa cells. In four cell lines, C8 activity was low and few cells died, phenocopying HeLa cells overexpressing FLIP (Fig8B). In a final line, C8 activity was high but cell death was low, phenocopying HeLa cells overexpressing Bcl-2 proteins (Fig8C). Based on these findings, we propose that about half of the cell lines in our sample exhibit similar regulation as the HeLa cells we have analyzed in detail, with rapid C8 activation, resulting in high levels of TRAIL-mediated cell death. These are the cells in which a C8 activation threshold is likely to play a dominant role in determining fractional killing. The factors most important in controlling TRAIL sensitivity are likely to differ in the other cell lines and may include negative regulation of DISC activity and reduced sensitivity to MOMP. Additional single-cell analysis and modeling should make it possible to create fate maps applicable to these and other cancer cell types.

## Discussion

In this paper, we investigate the basis of fractional cell killing by TRAIL and antibody agonists of DR4 and DR5 receptors. We demonstrate the existence of a threshold in initiator caspase activity (referred to as C8, since we do not distinguish caspases-8 and 10) that must be exceeded for cells to die. C8 dynamics can be described with considerable accuracy using a simple three-parameter phenomenological model that considers only the initial rate of caspase activation (*k*), the time subsequent to ligand addition at which C8 activity is maximal (τ), and a threshold θ for death. In cells that go on to die, C8 activity rises rapidly and monotonically until the threshold is reached and MOMP ensues. In cells that survive, C8 activity rises more slowly for 1–4 h, never achieving the level required for death, and then falls back to pre-treatment levels over the next 4–8 h due to proteasome-mediated protein degradation. The observed variation in C8 trajectories from cell to cell most likely arises from a combination of intrinsic and extrinsic noise (Spencer *et al*, 2009; Gaudet *et al*, 2012) that causes variation in *k* and τ: at constant agonist dose, *k* varies ∼tenfold across a cell population, and τ varies twofold to threefold. The probability that a cell will achieve a sufficiently high value of C8 activity to exceed the threshold and die is determined primarily by the C8 activation rate, *k*, which in turn depends on the concentration, identity, and oligomeric state of the DR4/5 agonist.

The best evidence that the threshold we observe in C8-DISC activity is biologically meaningful is that its value (its position in the landscape of *k* and τ) appears to be invariant for TRAIL, apomab (anti-DR5), and mapatumumab (anti-DR4) over a range of doses, in the presence and absence of bortezomib (which slows degradation of C8 and other proteins) and following overexpression of the resistance genes FLIP-S and FLIP-L. The position of the threshold θ does change with the level of Bcl-2 or Bcl-XL, or when Bcl-2 inhibitors such as ABT-263 are present, and is therefore set by the sensitivity of mitochondria for MOMP (i.e., the level of mitochondrial ‘priming’) (Deng *et al*, 2007). In this way, the C8-DISC threshold interacts with the classical MOMP threshold to determine the fraction of cells that live or die at any given level of DR4/5 activity.

The C8-DISC threshold we identify in HeLa cells is likely to be related to thresholds previously postulated to control the choice between MOMP-independent Type I and MOMP-dependent Type II apoptosis in response to Fas ligands (Algeciras-Schimnich *et al*, 2003) and to protein kinase CK2-mediated regulation of tBid cleavage (Hellwig *et al*, 2010), although we have not yet investigated these connections in detail. Cell death is largely cell autonomous in our experiments, but under different conditions, it is likely that cells can integrate information from neighboring cells and ‘remember’ previous exposure to TRAIL (Flusberg *et al*, 2013). Explaining such effects will require a more complex model, ultimately one that describes C8 activation kinetics in terms of the levels and precise biochemical activities of apoptosis regulators (Albeck *et al*, 2008b; Lopez *et al*, 2013).

### Impact of resistance genes and drugs that sensitize cells to extrinsic cell death

Experiments in a variety of tumor types have shown that sensitivity to TRAIL is controlled by numerous factors including the levels of expression of DR4, DR5, and C8, receptor glycosylation and resistance genes for decoy receptors and cFLIP (reviewed by Dimberg *et al* (2013)). In this paper, we examine two isoforms of one of the most common resistance genes and show that both cFLIP-S and cFLIP-L reduce *k*, the rate of C8 activation. Exposure of cFLIP-L-overexpressing cells to bortezomib restores sensitivity to TRAIL, and the magnitude of the effect is such that synergism between TRAIL and bortezomib is effectively infinite. However, bortezomib does not restore TRAIL sensitivity to cFLIP-S-overexpressing cells. Although similar biochemistry appears to be involved in both cases, we find that the impact of changes in *k* and τ on cell killing is highly nonlinear because of threshold effects. Bortezomib stabilizes C8—increasing τ and acting to promote apoptosis—while also stabilizing cFLIP-S and cFLIP-L—which decreases *k* and reduces apoptosis. However, cFLIP-S is sufficiently potent as a C8 competitor relative to cFLIP-L that a significant phenotypic difference is observed upon bortezomib co-administration.

The phenomenological model described by the landscape of *k* and τ helps to clarify our understanding of these phenomena in another way. FLIP regulates NF-κB-mediated survival pathways, and bortezomib is known to alter the levels of multiple factors that regulate NF-κB and apoptosis (e.g., IκBα (Hideshima *et al*, 2002), IAP, and Bcl-2 proteins (de Wilt *et al*, 2013)). However, the sufficiency of a simple C8 model in quantifying cell fate in FLIP-S- or FLIP-L-overexpressing cells in the presence and absence of bortezomib argues that the phenotypically consequential biochemistry for cell death lies at the level of DISC activity. This form of Occam's razor argues that all the other myriad changes induced by bortezomib or FLIP are substantially less significant for cell fate. By applying similar reasoning to other cell types and resistance genes, it should be possible to create an intelligible and actionable molecular model of TRAIL sensitivity and resistance across diverse cancer genotypes and to thereby predict the effects of new agents and drug combinations (Merino *et al*, 2006). A preliminary analysis of 9 additional cell types suggests that we will find several phenotypic classes, which we can approximately map to parental, c-FLIP-overexpressing, or Bcl-2-overexpressing HeLa cells.

### Implications for cancer therapy

The failure of multiple anti-DR4/5 therapeutic antibodies in clinical trials due to lack of efficacy has significantly dampened interest in this once-promising class of therapeutic agents. Particularly in the case of apomab, our data reveal an extraordinary lack of potency when compared to the recombinant TRAIL ligand. Even at concentrations above 100 nM, apomab induces C8 no better than 0.025 nM (1 ng/ml) TRAIL, a 4,000-fold difference. Low potency is also observed in the great majority of other cell lines we have examined, although there is evidence of exceptional responders among a subset of breast cancer and sarcoma cell lines (Zinonos *et al*, 2009). In this context, it should be noted that the TRAIL used in this paper is a recombinant form of the ligand that is artificially trimerized and almost certainly more potent than TRAIL produced naturally in the body or the therapeutic agent, dulanermin (Soria *et al*, 2010). Recombinant TRAIL is nonetheless useful as a comparator because it reveals the level of receptor activity that can in principle be achieved using an artificial agonist. In the case of apomab, low DISC activity is not caused by low binding affinity because we can saturate receptors with apomab (at ∼200 nM), at which point cell killing begins to fall as the concentration rises further. Antibody-mediated clustering has been shown to increase apomab potency (Adams *et al*, 2008), and the requirement for an agonist to bridge multiple receptors presumably explains squelching at high antibody concentrations. We find that clustering does increase *k* significantly but that this is insufficient to drive cells to a point in the landscape of *k* and τ where killing by apomab is efficient. We predict, and show experimentally, that efficient cell killing by apomab in HeLa cells requires a combination of clustering agent to increase *k* and bortezomib to increase τ. Rescuing DR4/5 agonists as a therapeutic class will require designing antibodies that can achieve values of *k* and τ that exceed θ in a large fraction of cancer cell types either individually or in combination with agents such as bortezomib; high-affinity binding to TRAIL receptors is not sufficient.

The variation we observe in θ with changes in the levels or activities of anti-apoptotic Bcl-2 proteins demonstrates a link between the C8-DISC threshold and the level of mitochondrial priming. In this way, cells integrate information from extrinsic and intrinsic pathways to set their overall sensitivity to death receptor agonists. Looking forward, it seems likely that a more complete biochemical model of the C8 and MOMP thresholds will reveal new ways to overcome TRAIL resistance in different cell types, perhaps by co-drugging to increase mitochondrial priming (Certo *et al*, 2006). The goal of future therapies must be to maximize *E*_max_ and not just IC_50_. Phenomenological models that account for cell-to-cell variability and the temporal dynamics of caspase activation are likely to prove useful in achieving this goal and also in identifying cell fate bifurcations controlling other forms of programmed cell death.

## Materials and Methods

### Cell lines and materials

The initiator caspase activity reporter (ICRP) and mitochondrial outer membrane permeabilization (MOMP) reporter (IMS-RP) were constructed as previously described (Albeck *et al*, 2008a). FLIP-L and FLIP-S plasmids were obtained from Dr. Inna Lavrik. Bcl-2/XL plasmids were obtained from Addgene (Cambridge, MA). mCherry-tagged versions of FLIP-L, FLIP-S, Bcl-2, and Bcl-XL were constructed by PCR, C-terminus ligation to mCherry, and cloning into pQCXIP (Clontech). HeLa cells were obtained from the ATCC and cultured in DMEM supplemented with 10% fetal bovine serum, 5 mM L-glutamine, and penicillin/streptomycin (Gibco). HeLa cells stably expressing combinations of ICRP and IMS-RP were derived by infection with retrovirus (293 Transfection Clontech, Mountain View, CA), double-positive selection by FACS, single-cell sorting, and were grown in 96-well plates until 1 colony appeared. Freshly cloned cells (below passage 10) were used in all experiments. FLIP-L-, FLIP-S-, Bcl-2-, and Bcl-XL-mCherry-expressing cells were obtained by infection of retrovirus encoding the respective mCherry-fusion gene into the parental clone of HeLa ICRP cells. After infection, a heterogeneous cell population was maintained so we could probe the consequences of varied levels of each mCherry-tagged protein. Recombinant human (rh) TRAIL was obtained from R&D Systems (Minneapolis, MN). apomab and mapatumumab were gracious gifts from Merrimack Pharmaceuticals, Inc. (Cambridge, MA; see Supplementary Materials and Methods). Donkey F(ab′)_2_ anti-human Fcγ was purchased from Jackson ImmunoResearch Laboratories, Inc. (West Grove, PA).

### Immunoblotting

Cells were lysed in Laemmli sample buffer; proteins were separated by SDS–PAGE (50 μg per lane, unless otherwise noted) and transferred to PVDF membranes. Membranes were blocked with Odyssey Blocking Buffer (LiCor) and probed with antibodies against caspase-8 (Cell Signaling Technologies), FLIP (NF6, obtained from Pr. Inna Lavrik), DR4 and DR5 (ProSci, Poway, CA), GFP (Abcam, Cambridge, MA), or β-Actin (Sigma, St. Louis, MO). Binding was detected via secondary antibodies conjugated with IRDye800 (Rockland Immunochemicals) or Alexa Fluor 680 (Invitrogen) using a LiCor Odyssey scanner. Protein levels were quantified using the LiCor analysis toolbox. Average pixel intensity was calculated for uniform rectangular regions framing individual bands, and the mean pixel intensity around each rectangular region of interest was used for background subtraction.

### Determination of FLIP-mCherry protein number in individual cells in microscopy experiments

FLIP-mCherry-expressing cell populations were sorted by FACS into four pools: low, medium, high, and highest mCherry expression levels. Quantitative Western blotting for FLIP relative to recombinant FLIP was performed to determine the mean number of proteins per cell in each pool. Based on a linear interpolation of these measurements, we inferred the expression level of FLIP in a single cell based on its mCherry fluorescence measured by FACS. Finally, we matched the distribution obtained by FACS to the distribution of mCherry fluorescence measured by microscopy to obtain an estimation of the level of FLIP in each cell used in the microscopy experiments.

### High-throughput live-cell imaging and analysis

Clonal HeLa cells stably expressing the FRET-based initiator caspase reporter (ICRP) were seeded into 96-well plates coated with rat-tail collagen I (BD, Franklin Lakes, NJ). Cells were imaged every 5 min for up to 24 h in the live-cell chamber of an Operetta robotic microscope (Perkin Elmer, Waltham, MA; see Supplementary Materials and Methods for details). For image analyses, cell segmentation, intensity readouts, tracking, evaluation of cell death and MOMP time, and trajectory fitting were performed using MATLAB (Natick, MA) with scripts developed in house (see Matlab Code in the Supplementary Information). See also Supplementary Materials and Methods for details.

### Trajectory fitting

The average FRET ratio trajectory of all untreated cells was subtracted from the FRET ratio trajectory of each treated cell. Noise in the trajectories was then filtered using the MATLAB function *filtfilt* with a windows size corresponding to 55 min (11 frames). For each trajectory, the minimal value of the FRET ratio was subtracted from the trajectory. We computed the derivative of the FRET ratio using finite differences and filtered the trajectories using the MATLAB function *filtfilt* with a window size corresponding to 55 min (11 frames).

The fitted model is based on the equation:

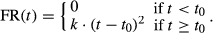


The time of maximal value for the derivative of the FRET ratio (τ) was used as the end point to fit the model to the FRET ratio using the MATLAB function *fit*. The parameter *t*_0_ was constrained to be in the range [−30 min, τ − 30 min] and k to [0, 0.01]. In cases in which the fit to a trajectory was bad (*r*^2^ < 0.5), we tried to improve the fit by ending the fitting on a secondary maximum; the fit with the best *r*^2^ was kept. All fits were tested for significance using an F-test against a flat model (FR(*t*) = *cste*) with *P* = 0.05 as a cutoff. In cases in which the fit was not significantly better than the flat model, *k* was set to the value 10^−7^ (minimum value observed for a fit). Cells that died early (at times less than 70 min) and whose trajectory could not be fitted by the equation above were discarded from subsequent analysis on the assumption that they represented other forms of cell death or loss. All single-cell trajectory data used in this paper and the values of fitted parameters are available at http://lincs.hms.harvard.edu/roux-molsystbiol-2015 to allow others to explore different analytical approaches.

### Determining the value θ

The value θ was found by minimizing the error function: 

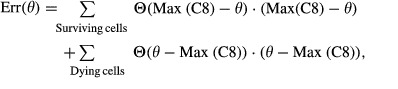
where Θ is the Heaviside function: 
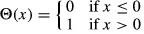


The value θ_*T*_ = 2.63 × 10^−3^ was the value that minimizes the error function across all experiments of the parental HeLa cells treated with doses of TRAIL equal to or above 10 ng/ml and no other drug; θ_*T25*_ was the value computed using data for 25 ng/ml TRAIL only. θ_*T*_ and θ_*T*25_ were not significantly different from each other despite the use of different training data. We used the value θ_*T*_ in the analyses of antibody agonists and cells co-drugged with TRAIL and either bortezomib or ABT-263. For the plot in Fig7A and B, we obtained an optimal value for θ_ABT_ using replicate experiments of cells exposed to 25 ng/ml TRAIL and 10 μM ABT-263.

### Data availability

The data relevant to this study are available at http://lincs.hms.harvard.edu/roux-molsystbiol-2015. Data in tabular format are provided as Supplementary Datasets S1, S2, S3 and S4, and the scripts for the image and data analyses are provided as well (Matlab Code).

## Supporting Information

Supplementary Materials and Methods, Supplementary Figure Legends

Supplementary Matlab_Code

Review Process File
