# Supplementary material for: Fractional killing arises from cell-to-cell variability in overcoming a caspase activity threshold
Source: Mol Syst Biol. 2015 May 7;11(5):803. doi: 10.15252/msb.20145584 (PMC4461398; doi:10.15252/msb.20145584)
Supplement: Supplementary file 1 [file msb0011-0803-sd1.pdf]

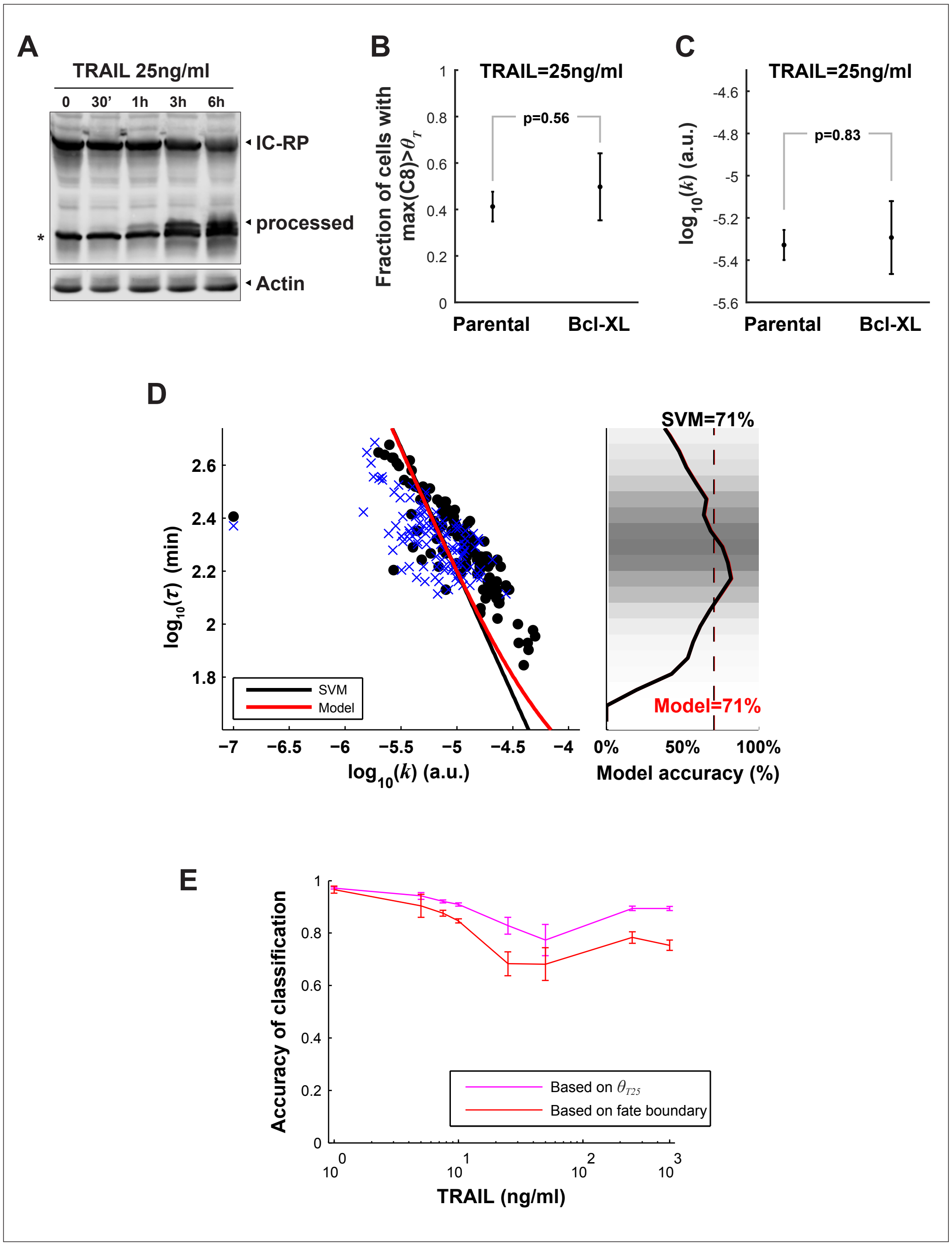

A
TRAIL 25ng/ml
0 30' 1h 3h 6h
IC-RP
processed
*
Actin
B
TRAIL=25ng/ml
Fraction of cells with max(C8)>θ_T
p=0.56
Parental
Bcl-XL
C
TRAIL=25ng/ml
$\log_{10}(k)$ (a.u.)
p=0.83
Parental
Bcl-XL
D
$\log_{10}(\tau)$ (min)
$\log_{10}(k)$ (a.u.)
SVM
Model
SVM=71%
Model=71%
0% 50% 100%
Model accuracy (%)
E
Accuracy of classification
Based on θ_{T25}
Based on fate boundary
TRAIL (ng/ml)

Figure S1

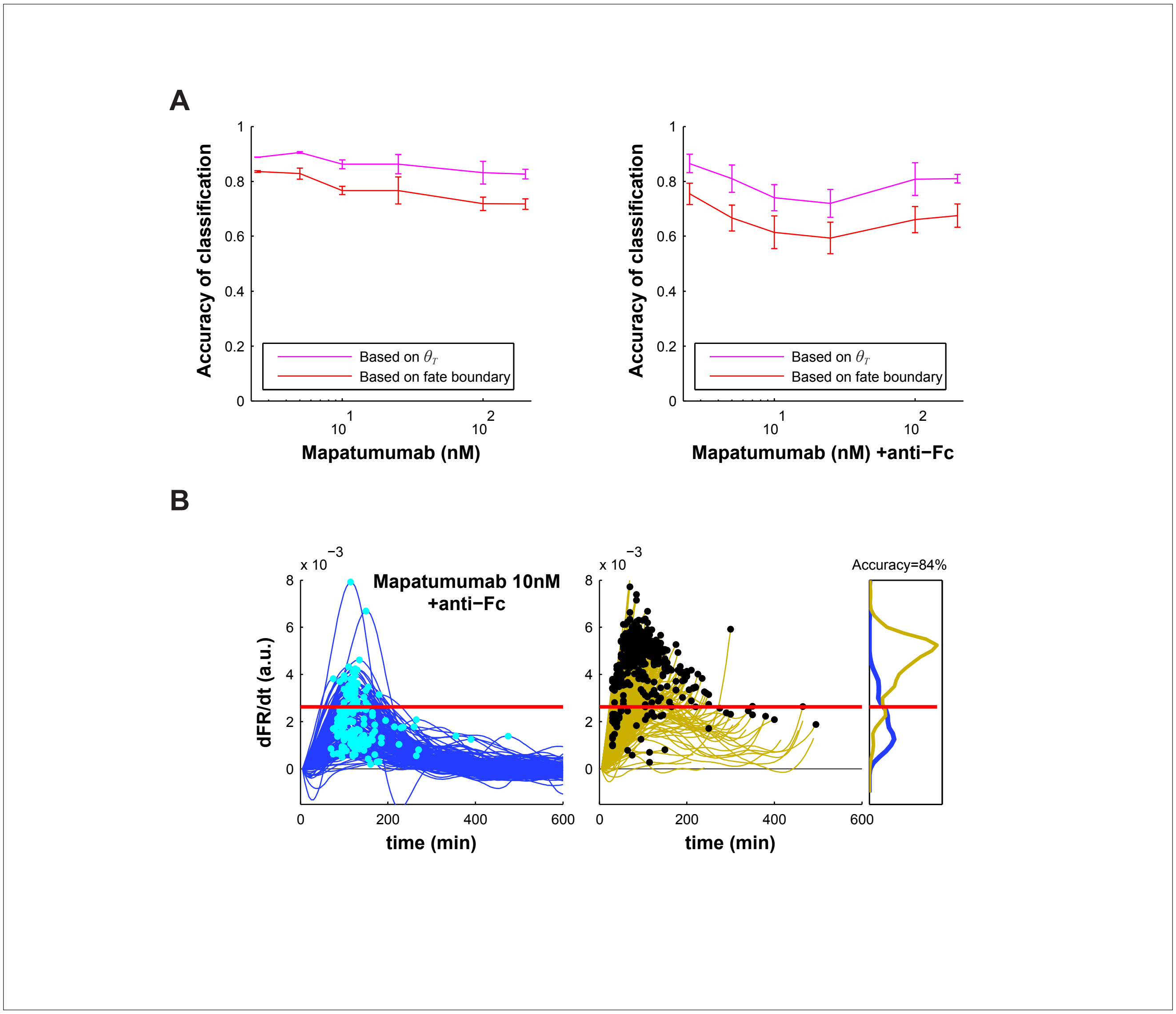
A
Accuracy of classification
Based on θ_T
Based on fate boundary
Mapatumumab (nM)
Mapatumumab (nM) +anti−Fc
B
dFR/dt (a.u.)
x 10^−3
Mapatumumab 10nM +anti−Fc
Accuracy=84%
time (min)

Figure S2

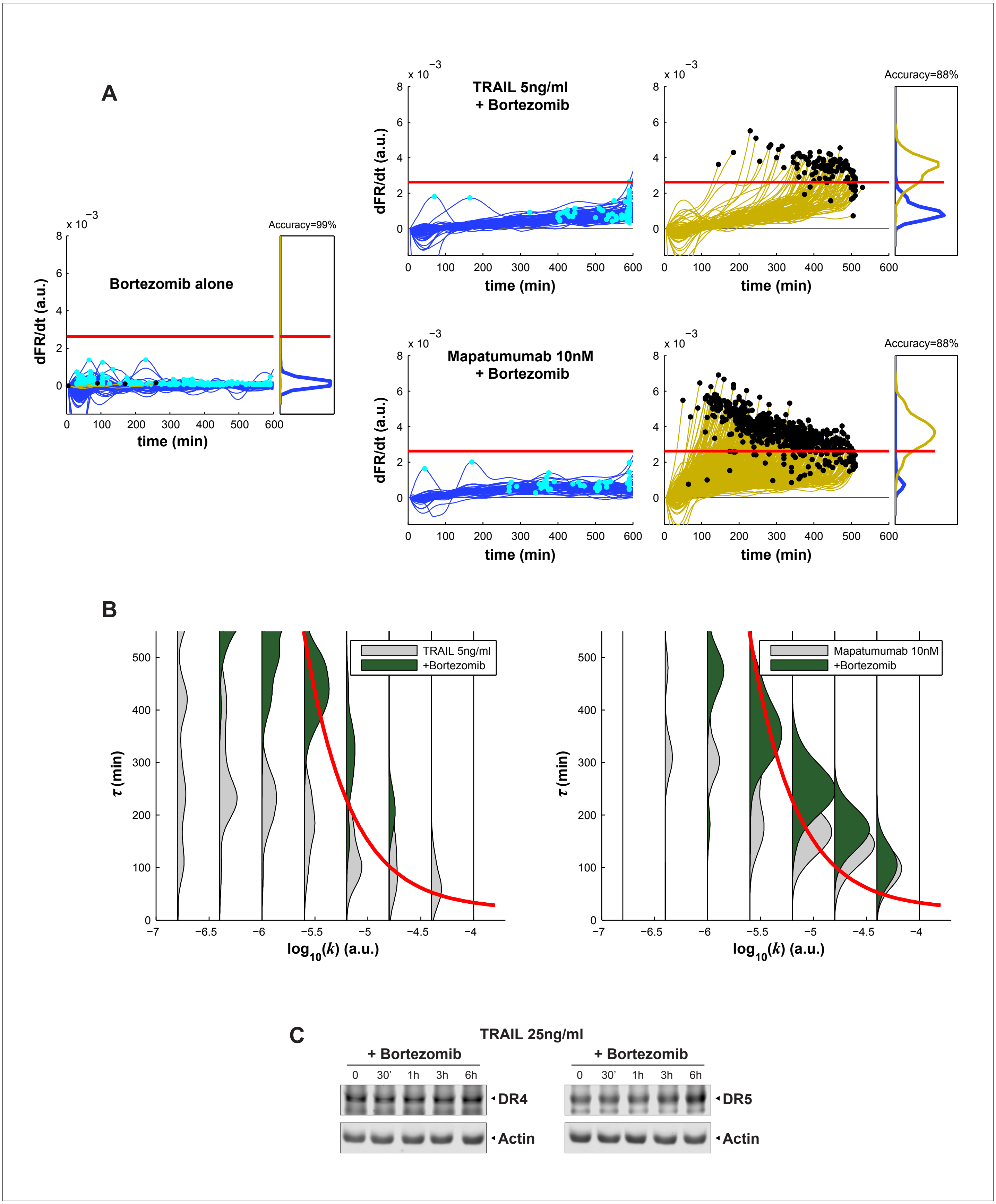
A
Bortezomib alone
Accuracy=99%
dFR/dt (a.u.)
time (min)
TRAIL 5ng/ml
+ Bortezomib
Accuracy=88%
Mapatumumab 10nM
+ Bortezomib
Accuracy=88%
B
TRAIL 5ng/ml
+Bortezomib
Mapatumumab 10nM
+Bortezomib
τ (min)
log10(k) (a.u.)
C
TRAIL 25ng/ml
+ Bortezomib
0 30' 1h 3h 6h
DR4
DR5
Actin

Figure S3

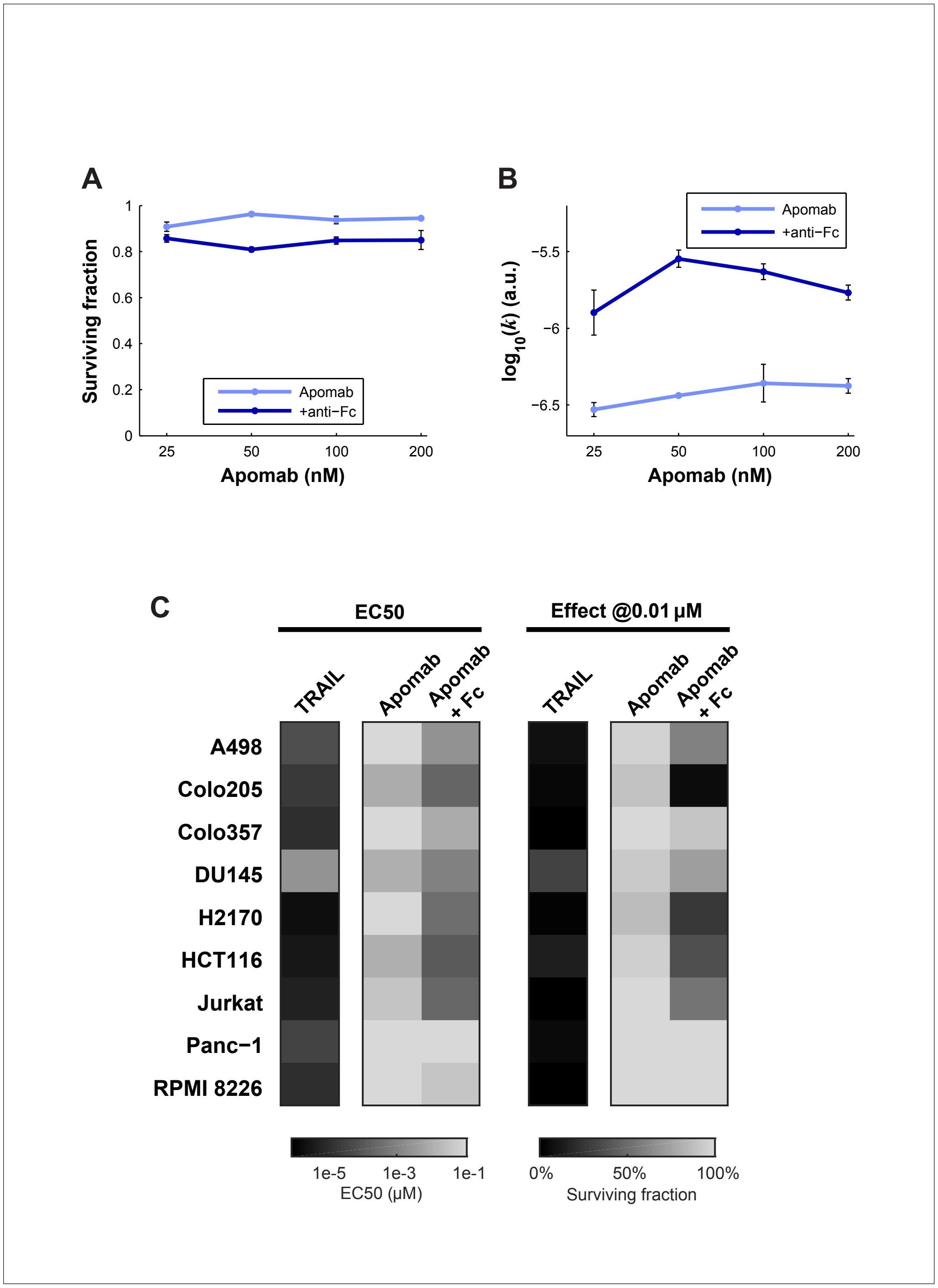
A
Surviving fraction
Apomab
+anti−Fc
Apomab (nM)
B
log10(k) (a.u.)
Apomab
+anti−Fc
Apomab (nM)
C
EC50
Effect @0.01 μM
TRAIL
Apomab
Apomab + Fc
A498
Colo205
Colo357
DU145
H2170
HCT116
Jurkat
Panc−1
RPMI 8226
1e-5
1e-3
1e-1
EC50 (μM)
0%
50%
100%
Surviving fraction

Figure S4

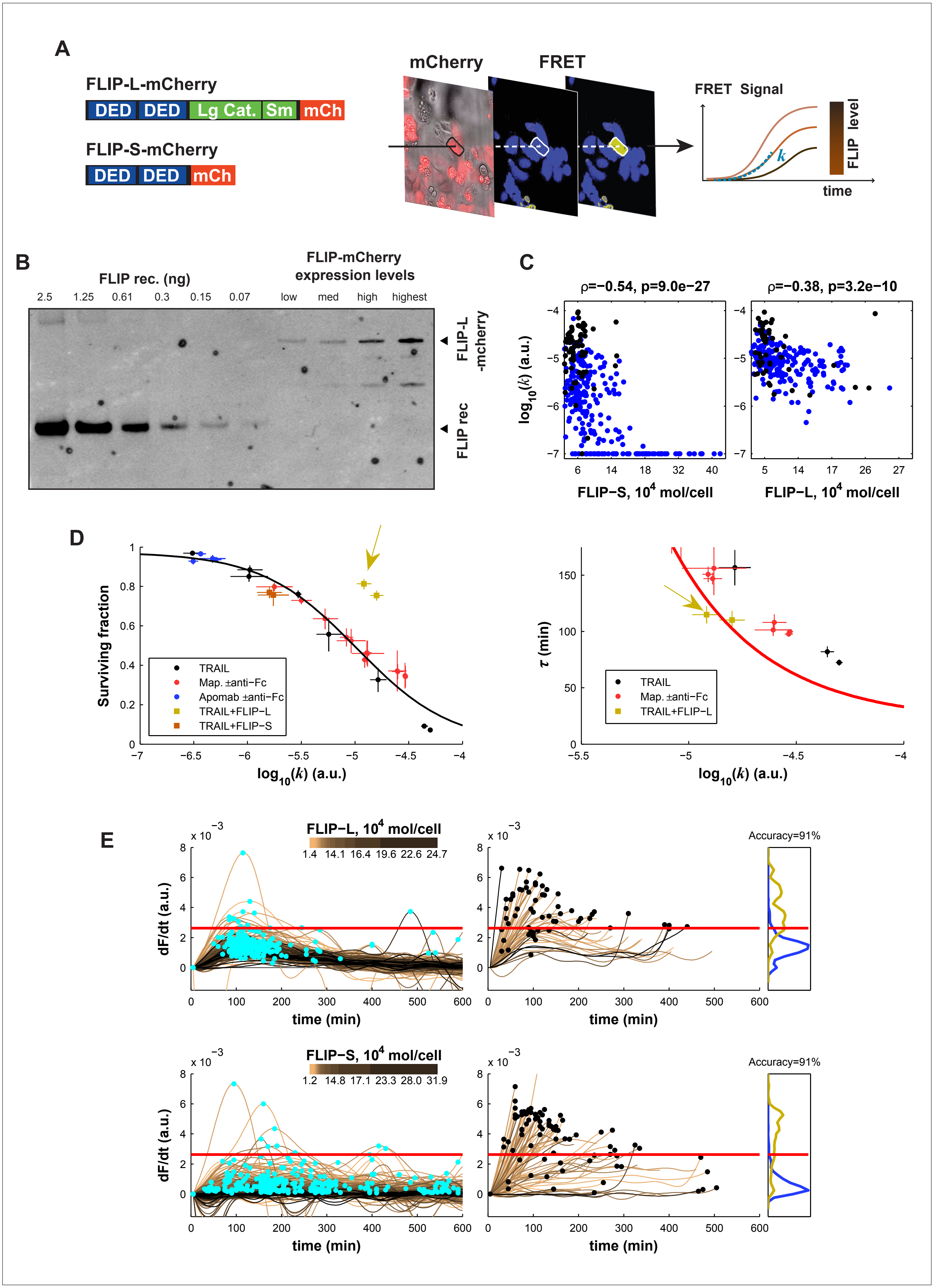

A
FLIP-L-mCherry
DED DED Lg Cat. Sm mCh
FLIP-S-mCherry
DED DED mCh
mCherry
FRET
FRET Signal
k
FLIP level
time
B
FLIP rec. (ng)
2.5 1.25 0.61 0.3 0.15 0.07
FLIP-mCherry expression levels
low med high highest
FLIP-L -mcherry
FLIP rec
C
ρ=−0.54, p=9.0e−27
ρ=−0.38, p=3.2e−10
log10(k) (a.u.)
FLIP−S, 10^4 mol/cell
FLIP−L, 10^4 mol/cell
D
Surviving fraction
TRAIL
Map. ±anti−Fc
Apomab ±anti−Fc
TRAIL+FLIP−L
TRAIL+FLIP−S
log10(k) (a.u.)
τ (min)
E
FLIP−L, 10^4 mol/cell
1.4 14.1 16.4 19.6 22.6 24.7
dF/dt (a.u.)
time (min)
Accuracy=91%
FLIP−S, 10^4 mol/cell
1.2 14.8 17.1 23.3 28.0 31.9
Accuracy=91%

Figure S5

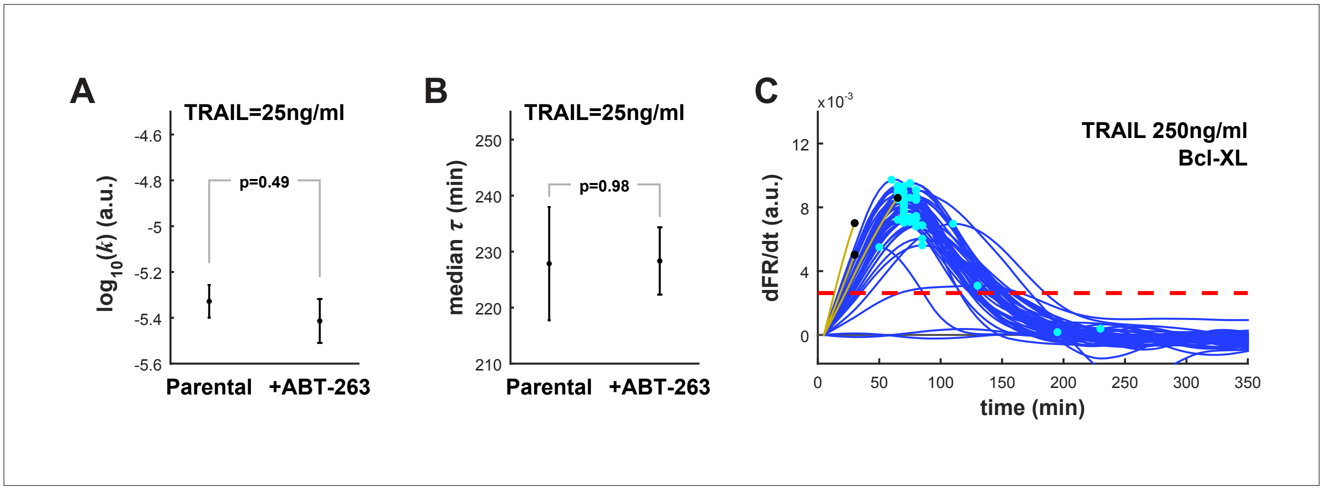
A
TRAIL=25ng/ml
-4.6
-4.8
-5
-5.2
-5.4
-5.6
log10(k) (a.u.)
p=0.49
Parental
+ABT-263
B
TRAIL=25ng/ml
250
240
230
220
210
median τ (min)
p=0.98
Parental
+ABT-263
C
x10-3
12
8
4
0
dFR/dt (a.u.)
TRAIL 250ng/ml
Bcl-XL
0
50
100
150
200
250
300
350
time (min)

Figure S6
