# Supplementary figures and images for: Fractional killing arises from cell-to-cell variability in overcoming a caspase activity threshold
Source: Mol Syst Biol. 2015 May 7;11(5):803. doi: 10.15252/msb.20145584 (PMC4461398; doi:10.15252/msb.20145584)

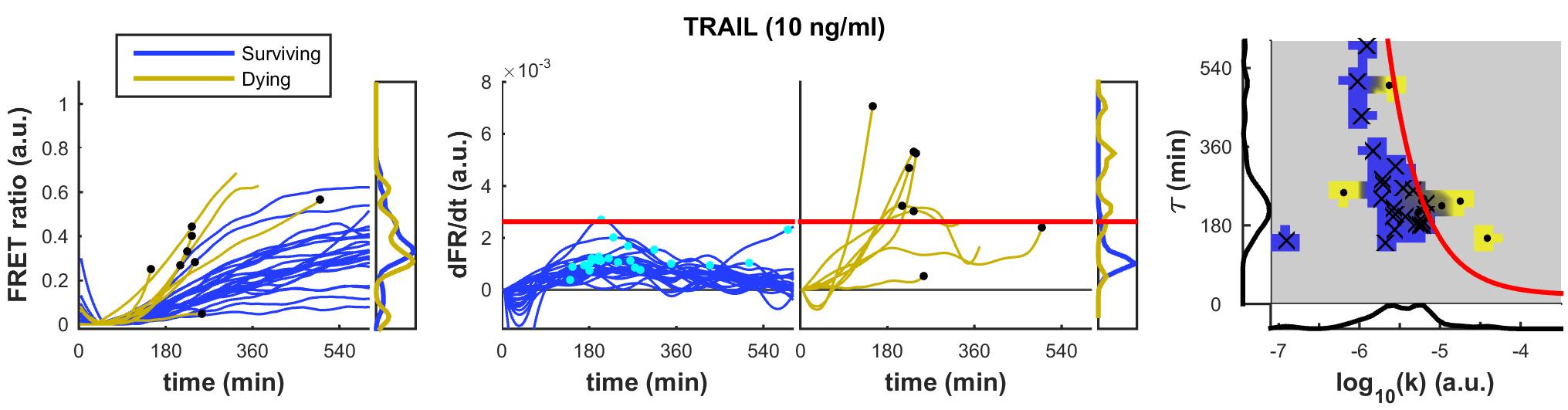

Supplement: Supplementary file 3 [file msb0011-0803-sd3.zip › All_data/010_TRAIL (10 ngml)_2/010_TRAIL (10 ngml)_2.jpg]

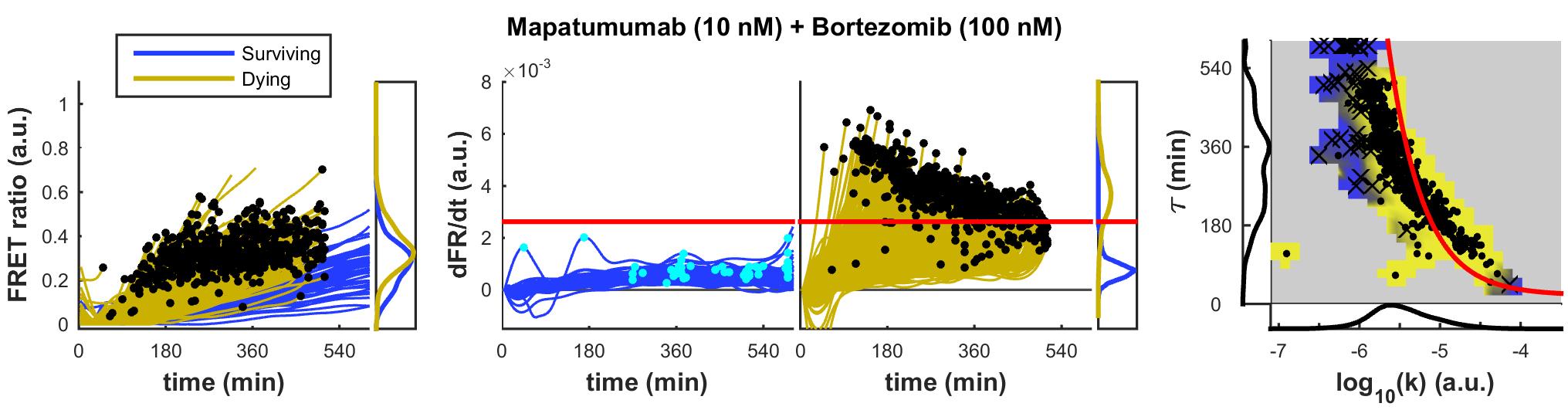

Supplement: Supplementary file 3 [file msb0011-0803-sd3.zip › All_data/086_Mapatumumab (10 nM) + Bortezomib (100 nM)_3/086_Mapatumumab (10 nM) + Bortezomib (100 nM)_3.jpg]

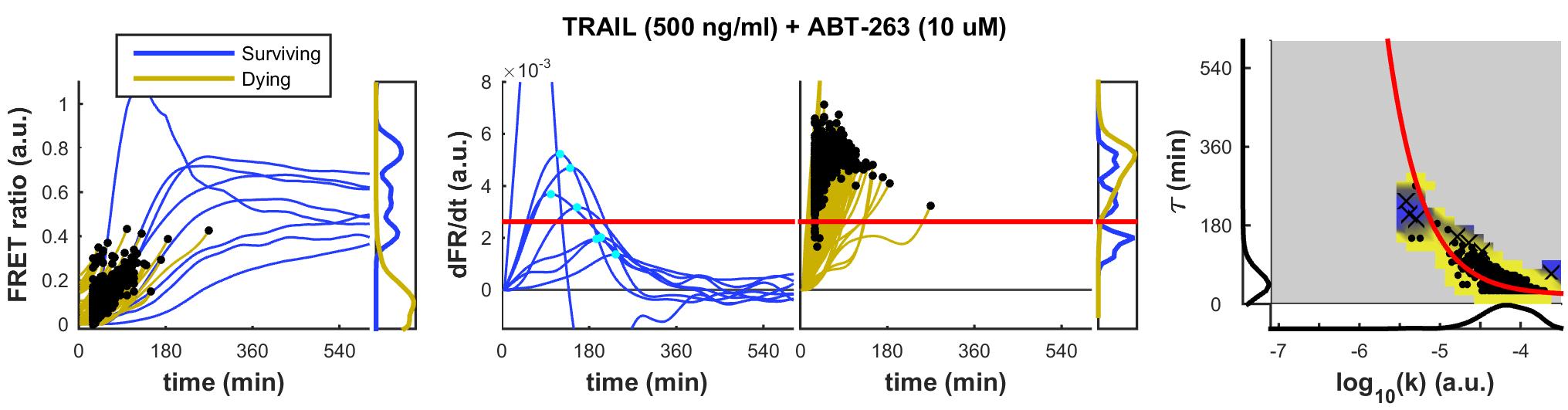

Supplement: Supplementary file 3 [file msb0011-0803-sd3.zip › All_data/051_TRAIL (500 ngml) + ABT-263 (10 uM)_2/051_TRAIL (500 ngml) + ABT-263 (10 uM)_2.jpg]

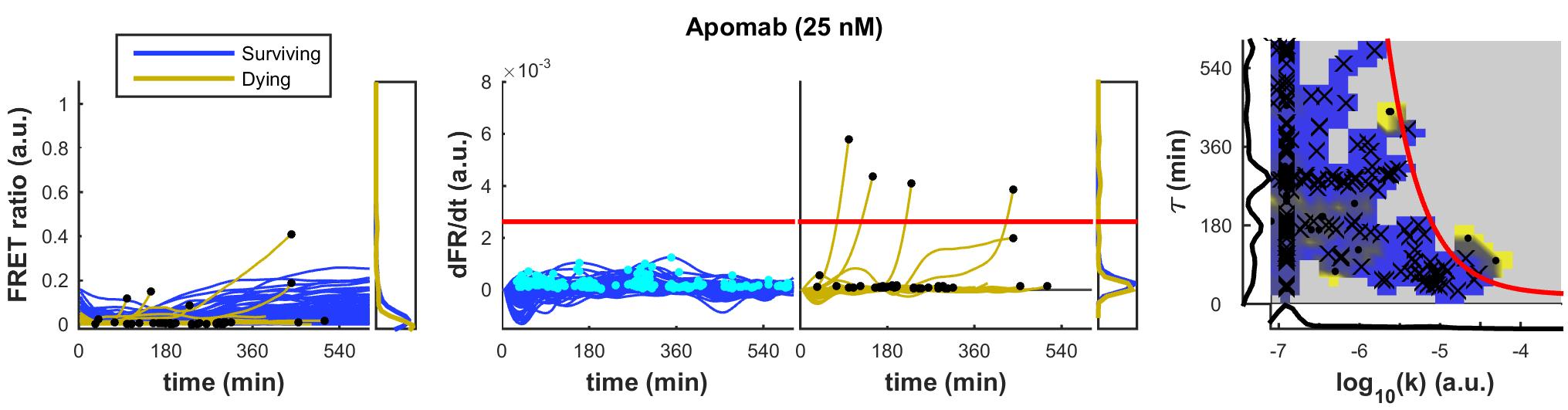

Supplement: Supplementary file 3 [file msb0011-0803-sd3.zip › All_data/092_Apomab (25 nM)_3/092_Apomab (25 nM)_3.jpg]

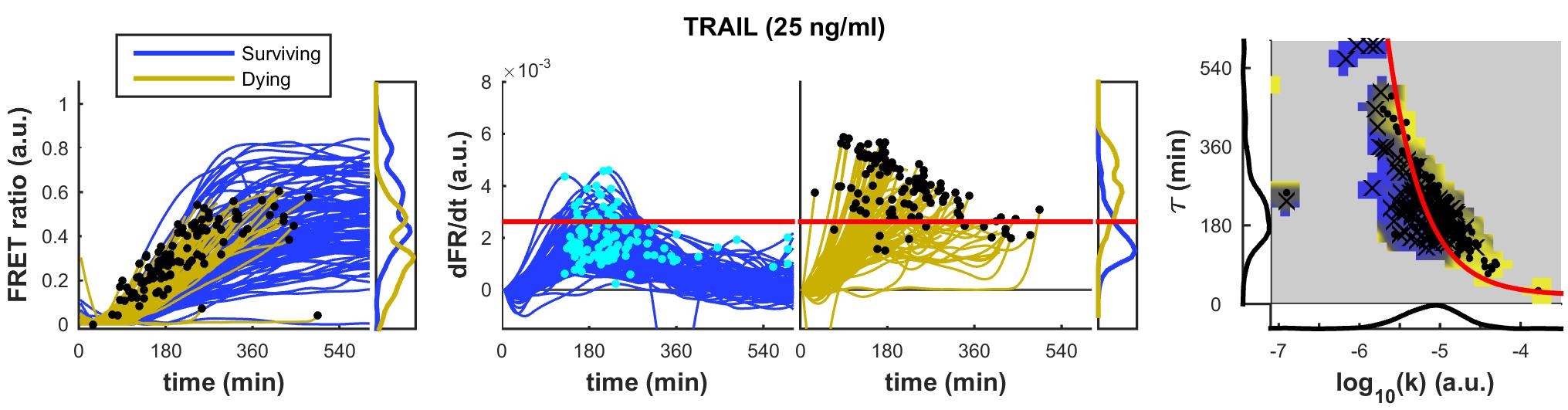

Supplement: Supplementary file 3 [file msb0011-0803-sd3.zip › All_data/012_TRAIL (25 ngml)_2/012_TRAIL (25 ngml)_2.jpg]

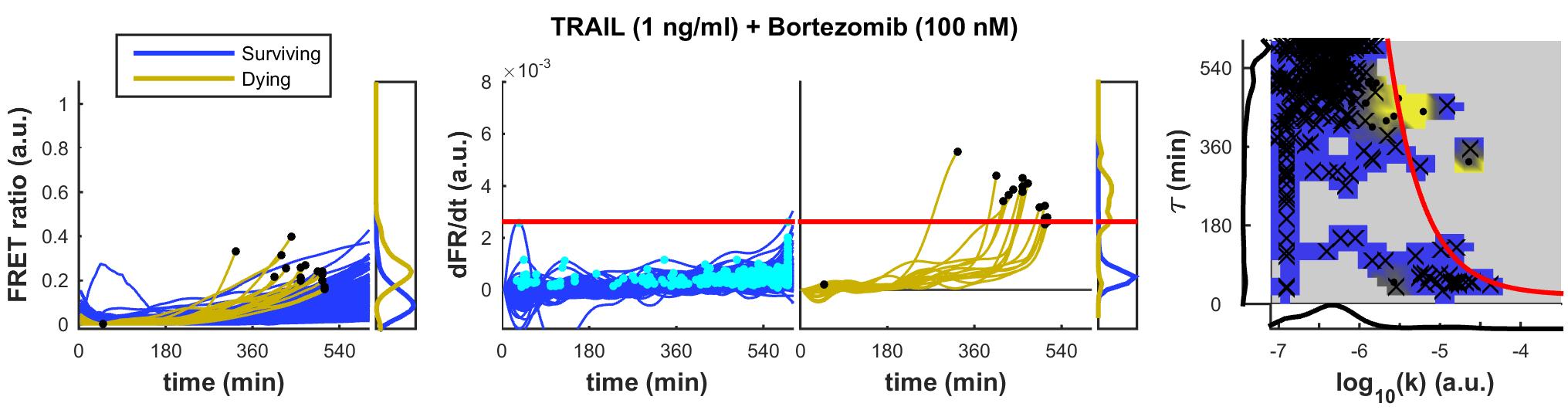

Supplement: Supplementary file 3 [file msb0011-0803-sd3.zip › All_data/028_TRAIL (1 ngml) + Bortezomib (100 nM)_2/028_TRAIL (1 ngml) + Bortezomib (100 nM)_2.jpg]

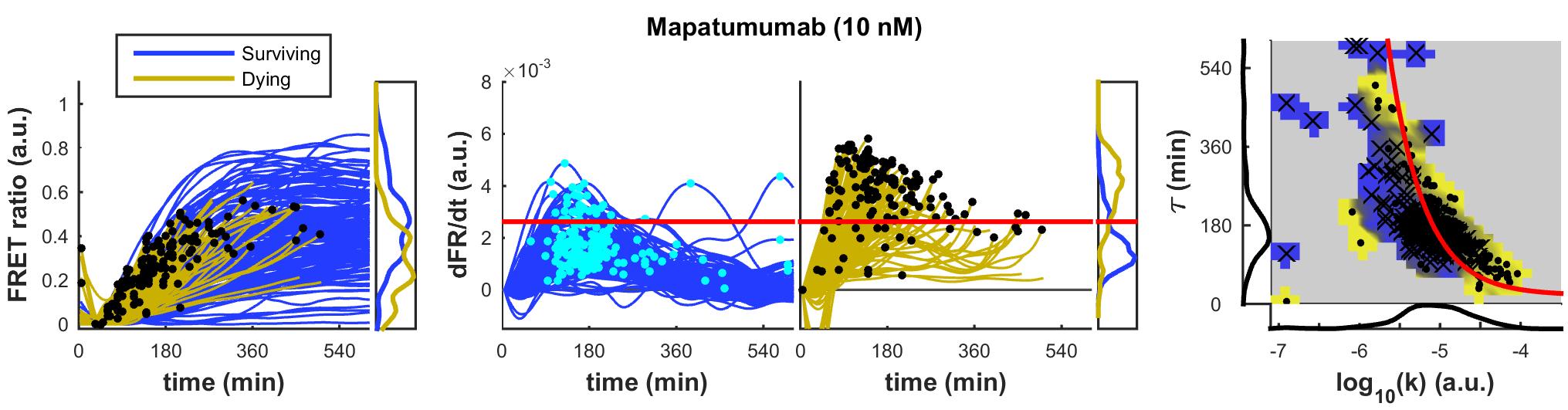

Supplement: Supplementary file 3 [file msb0011-0803-sd3.zip › All_data/058_Mapatumumab (10 nM)_2/058_Mapatumumab (10 nM)_2.jpg]

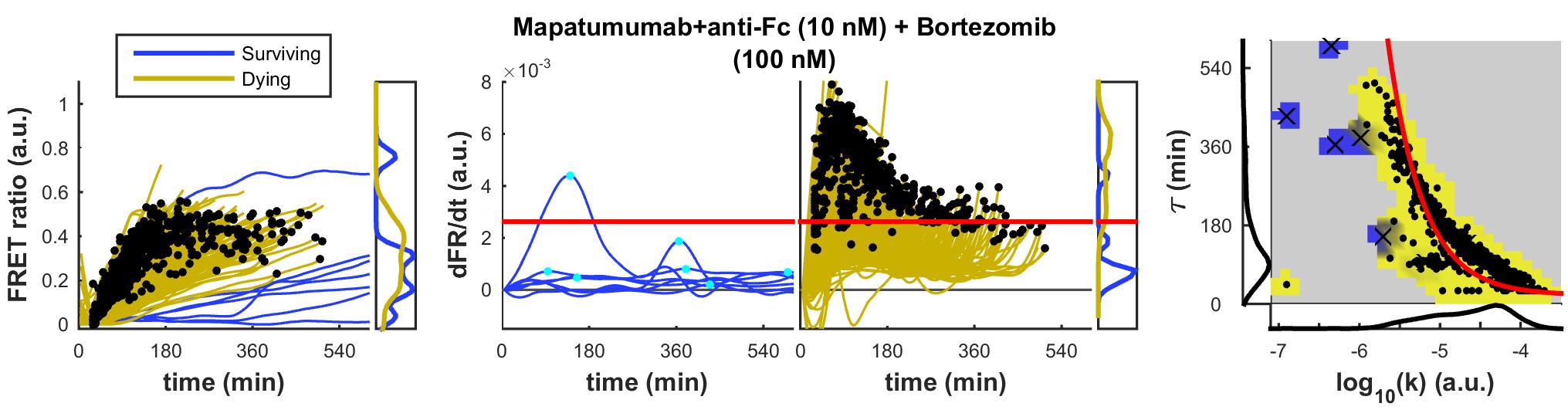

Supplement: Supplementary file 3 [file msb0011-0803-sd3.zip › All_data/087_Mapatumumab+anti-Fc (10 nM) + Bortezomib (100 nM)_1/087_Mapatumumab+anti-Fc (10 nM) + Bortezomib (100 nM)_1.jpg]

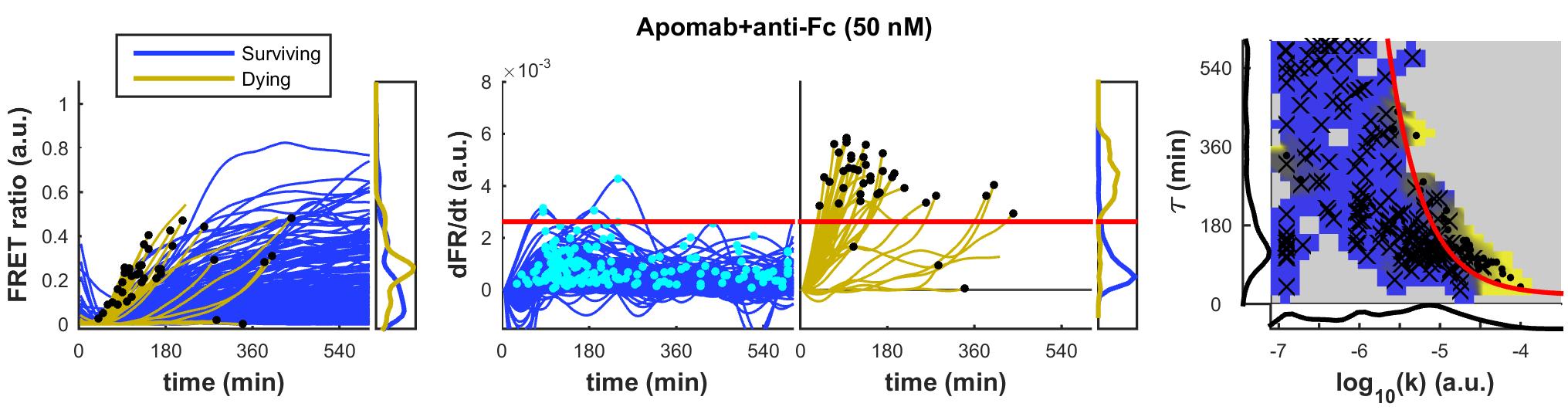

Supplement: Supplementary file 3 [file msb0011-0803-sd3.zip › All_data/110_Apomab+anti-Fc (50 nM)_2/110_Apomab+anti-Fc (50 nM)_2.jpg]

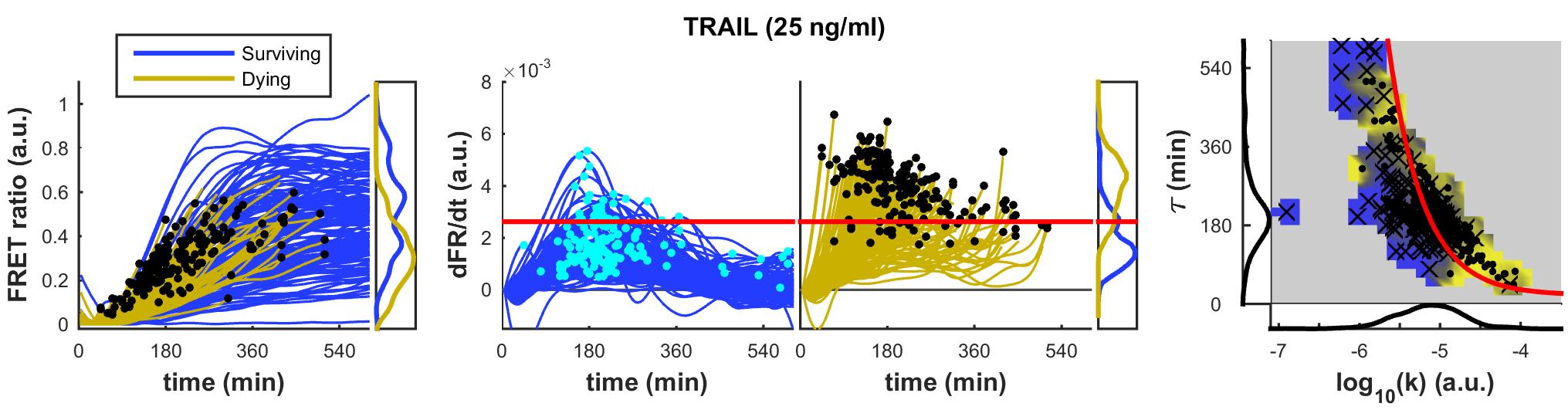

Supplement: Supplementary file 3 [file msb0011-0803-sd3.zip › All_data/013_TRAIL (25 ngml)_3/013_TRAIL (25 ngml)_3.jpg]

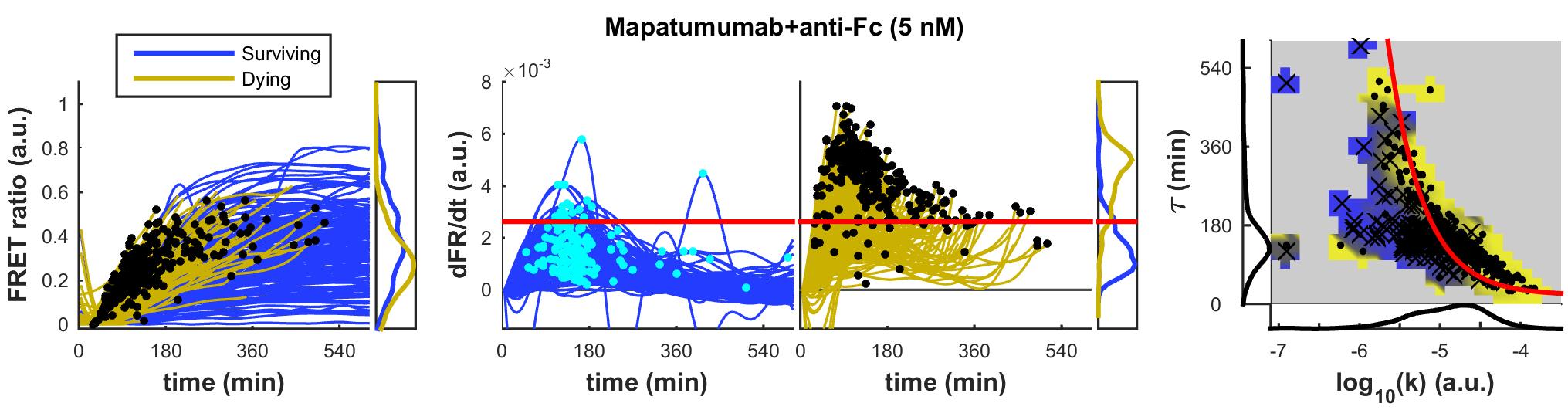

Supplement: Supplementary file 3 [file msb0011-0803-sd3.zip › All_data/072_Mapatumumab+anti-Fc (5 nM)_3/072_Mapatumumab+anti-Fc (5 nM)_3.jpg]

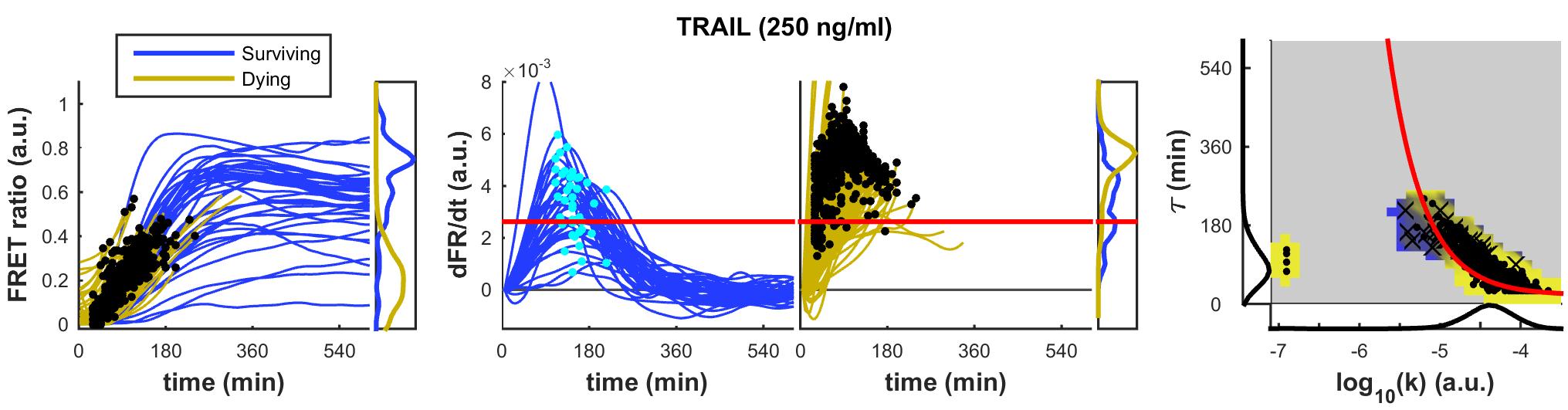

Supplement: Supplementary file 3 [file msb0011-0803-sd3.zip › All_data/020_TRAIL (250 ngml)_4/020_TRAIL (250 ngml)_4.jpg]

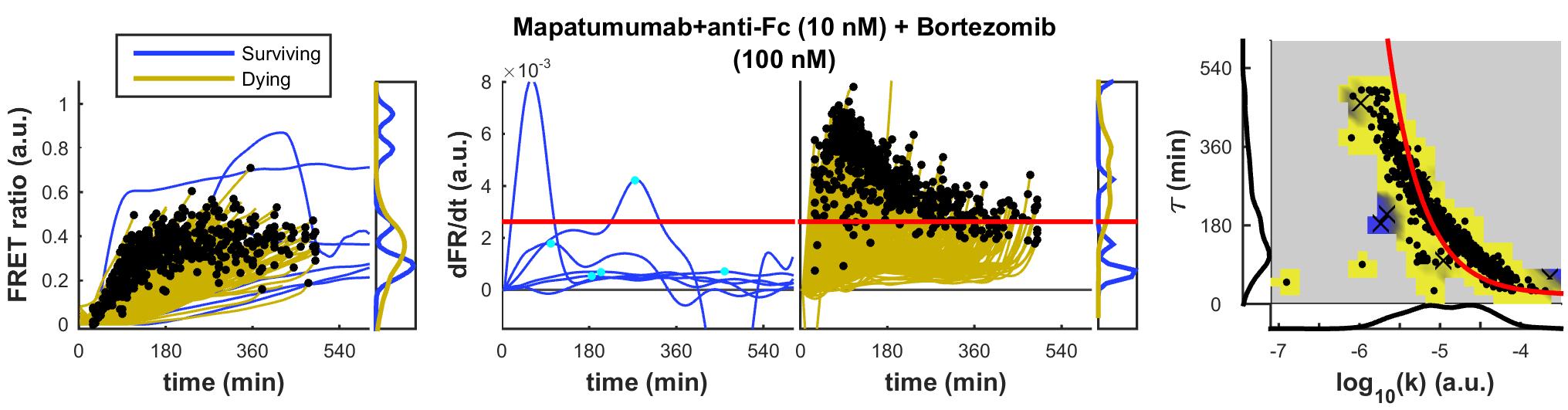

Supplement: Supplementary file 3 [file msb0011-0803-sd3.zip › All_data/089_Mapatumumab+anti-Fc (10 nM) + Bortezomib (100 nM)_3/089_Mapatumumab+anti-Fc (10 nM) + Bortezomib (100 nM)_3.jpg]

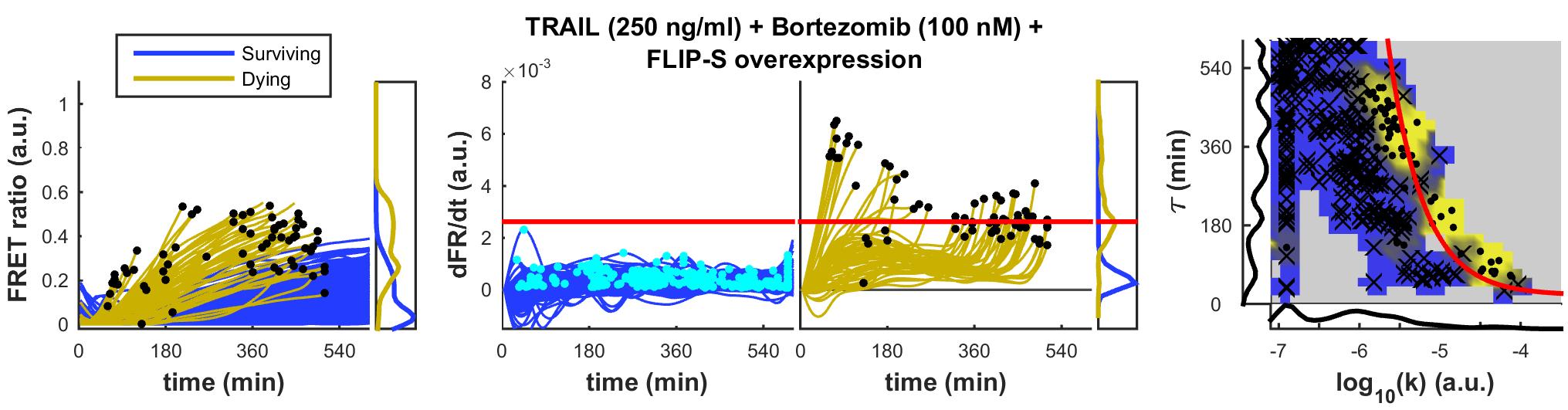

Supplement: Supplementary file 3 [file msb0011-0803-sd3.zip › All_data/143_TRAIL (250 ngml) + Bortezomib (100 nM) + FLIP-S overexpression_3/143_TRAIL (250 ngml) + Bortezomib (100 nM) + FLIP-S overexpression_3.jpg]

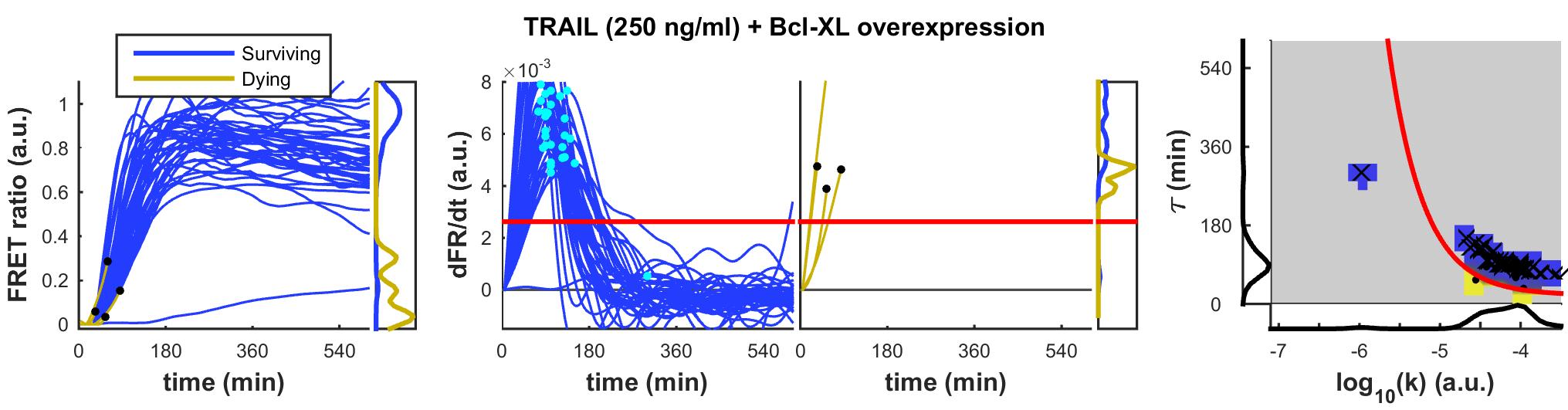

Supplement: Supplementary file 3 [file msb0011-0803-sd3.zip › All_data/154_TRAIL (250 ngml) + Bcl-XL overexpression_2/154_TRAIL (250 ngml) + Bcl-XL overexpression_2.jpg]

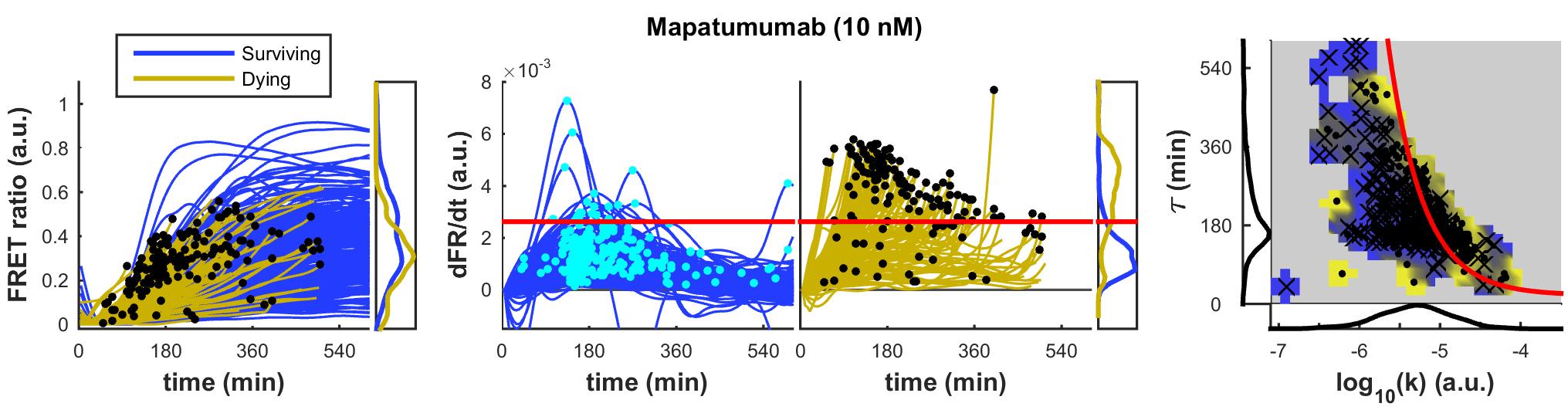

Supplement: Supplementary file 3 [file msb0011-0803-sd3.zip › All_data/059_Mapatumumab (10 nM)_3/059_Mapatumumab (10 nM)_3.jpg]

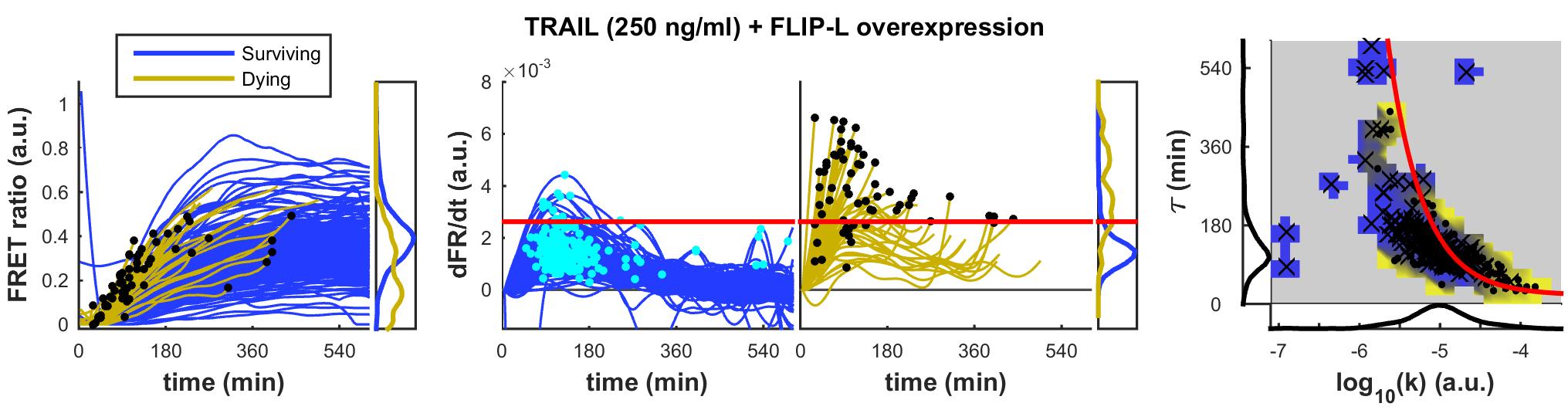

Supplement: Supplementary file 3 [file msb0011-0803-sd3.zip › All_data/124_TRAIL (250 ngml) + FLIP-L overexpression_1/124_TRAIL (250 ngml) + FLIP-L overexpression_1.jpg]

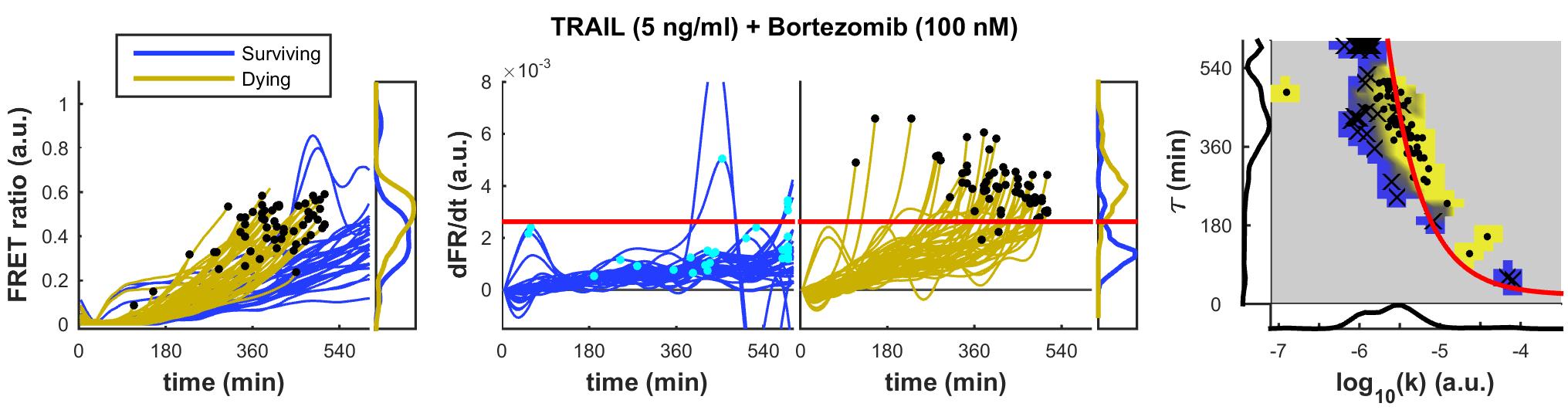

Supplement: Supplementary file 3 [file msb0011-0803-sd3.zip › All_data/031_TRAIL (5 ngml) + Bortezomib (100 nM)_3/031_TRAIL (5 ngml) + Bortezomib (100 nM)_3.jpg]

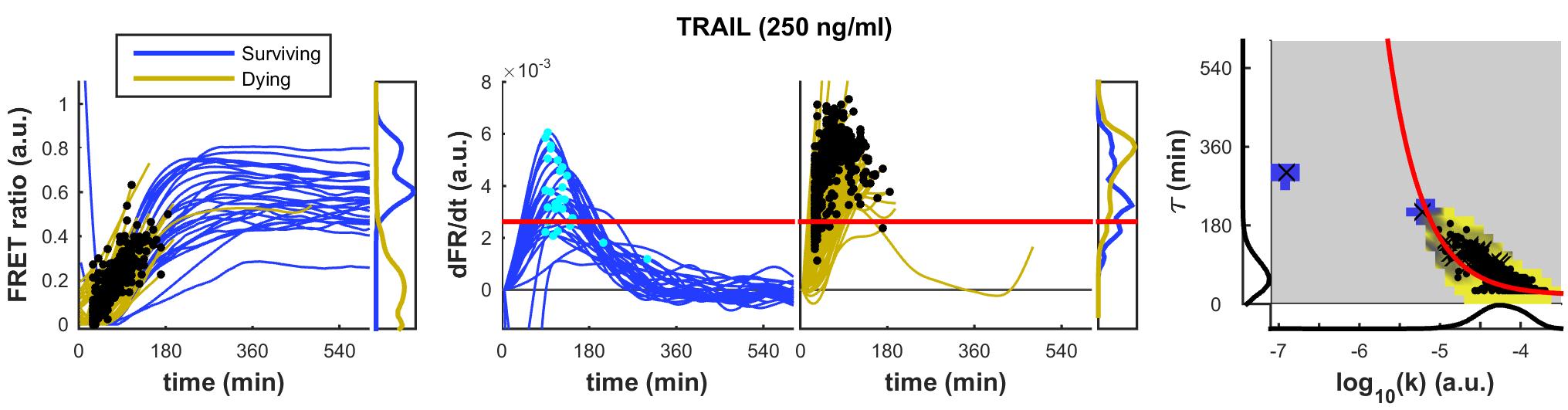

Supplement: Supplementary file 3 [file msb0011-0803-sd3.zip › All_data/017_TRAIL (250 ngml)_1/017_TRAIL (250 ngml)_1.jpg]

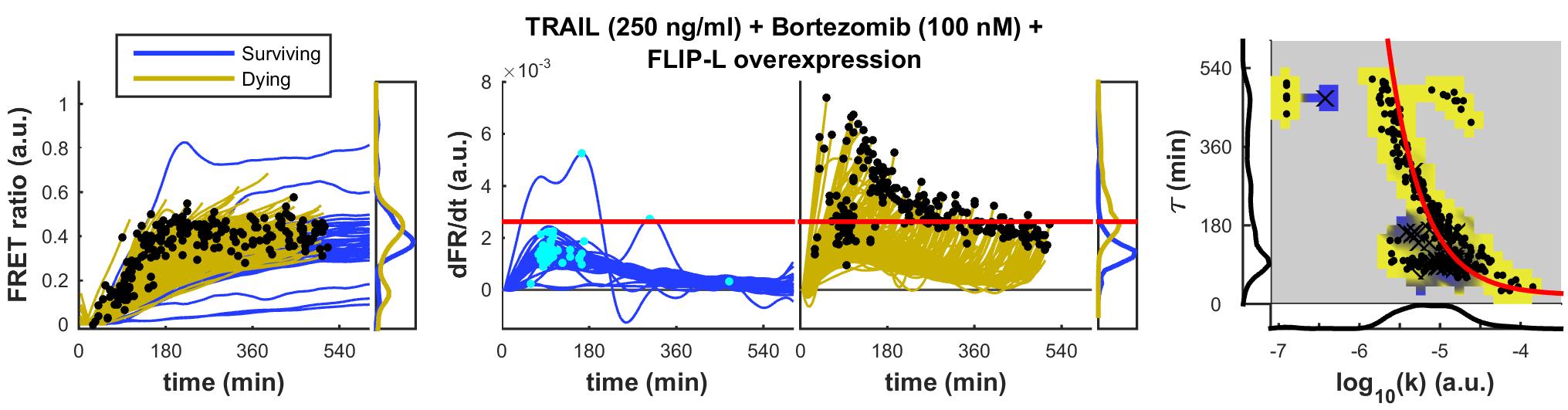

Supplement: Supplementary file 3 [file msb0011-0803-sd3.zip › All_data/139_TRAIL (250 ngml) + Bortezomib (100 nM) + FLIP-L overexpression_2/139_TRAIL (250 ngml) + Bortezomib (100 nM) + FLIP-L overexpression_2.jpg]

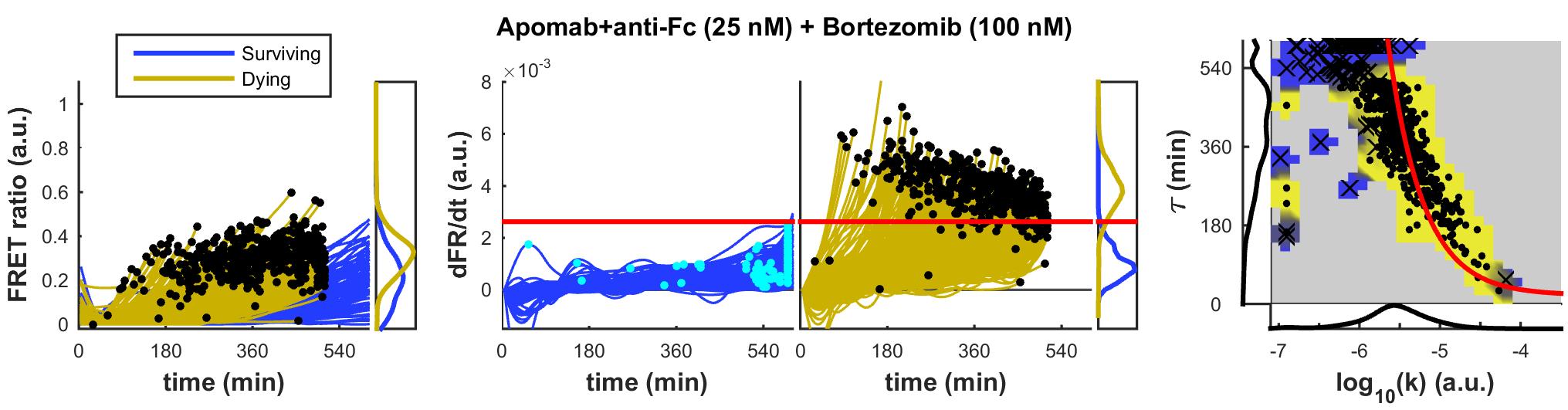

Supplement: Supplementary file 3 [file msb0011-0803-sd3.zip › All_data/123_Apomab+anti-Fc (25 nM) + Bortezomib (100 nM)_4/123_Apomab+anti-Fc (25 nM) + Bortezomib (100 nM)_4.jpg]

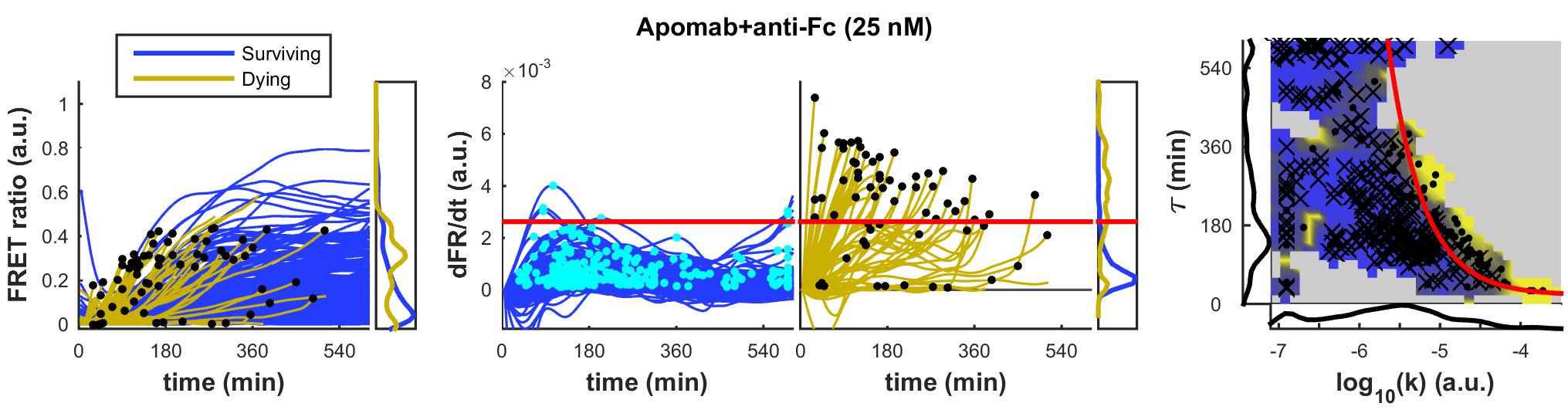

Supplement: Supplementary file 3 [file msb0011-0803-sd3.zip › All_data/105_Apomab+anti-Fc (25 nM)_3/105_Apomab+anti-Fc (25 nM)_3.jpg]
