# Supplementary material for: Fractional killing arises from cell-to-cell variability in overcoming a caspase activity threshold
Source: Mol Syst Biol. 2015 May 7;11(5):803. doi: 10.15252/msb.20145584 (PMC4461398; doi:10.15252/msb.20145584)
Supplement: Supplementary file 6 [file msb0011-0803-sd6.docx]

## Supplementary Figure legends

## Figure S1: DISC dynamics predict cell fate after TRAIL treatments.

(**A**) Western blot analysis at different time points for the ICRP reporter after treatments with 25ng/ml of TRAIL in HeLa ICRP cells overexpressing Bcl-2-mCherry.

(**B**) Fraction of cells with *Max(C8)* greater than *θ_T_* for parental cells compared to Bcl-XL-overexpressing cells after treatment with 25 ng/ml of TRAIL. Data are represented as mean ± SEM.

(**C**) Mean of the *log_10_(k)* for parental cells compared to Bcl-XL-overexpressing cells after treatment with 25 ng/ml of TRAIL. Data are represented as mean ± SEM.

(**D**) Comparison of the fate boundary and a support vector machine (SVM) classifier. Left panel: caspase-8 activity landscape defined by *log_10_(k)* and *log_10_(τ)* for the same experiment as in Fig. 1A and 1D of the main text. Surviving cells are cyan crosses; dead cells are black dots. The red line is the fate boundary calculated with EQ3. The black line is a linear SVM classifier. Right panel: comparison of the prediction accuracy between the fate boundary and the SVM classifier as a function of *τ*. The background shades represent the cell density at different values of *τ*.

(**E**) Classification accuracy based on the value of *Max(C8)* and *θ_T25_* = 2.61×10^-3^ (in purple) or on the fate boundary (EQ3) in the landscape of *k* and *τ* (in red) for different doses of TRAIL. Data are shown as mean ± SEM.

## Figure S2: Classification accuracy for Mapatumumab treatments.

(**A**) Classification accuracy based on the value of *Max(C8)* and *θ_T_* = 2.6×10^-3^ (in purple) or on the fate boundary (EQ3) in the landscape of *k* and *τ* (in red) for different doses of Mapatumumab without (left) and with (right) anti-Fc in HeLa ICRP cells. Data are shown as mean ± SEM.

(**B**) Derivative of FRET ratio for surviving cells (blue, left panel) and cells committing to apoptosis (yellow, middle panel) following treatment of HeLa ICRP cells with 10 nM of Mapatumumab with anti-Fc. Cyan and black dots show the maximal value of the derivative (note that black dots also indicate cell death). The right panel shows the distribution of maximal values of the derivative for both populations. The red line indicates the value *θ_T_* = 2.6× 10^-3^ that separates the two populations with 84% accuracy respectively.

## Figure S3: Bortezomib induces TRAIL-dependent apoptosis by increasing the duration of caspase-8 activation.

**(A)** Derivative of FRET ratio for surviving cells (blue, left panel) and cells committing to apoptosis (yellow, middle panel) following treatment of HeLa ICRP cells with 5ng/ml of TRAIL (upper panels, same experiment as in Fig. 3C) or 10nM of Mapatumumab (lower panels, same experiment as in Fig. 3F), in presence (right two panels) or absence (left panel) of bortezomib. Cyan and black dots show the maximal values of the derivative (note that black dots also indicate cell death). The right panel shows the distribution of maximal values of the derivative for both populations. The red line indicates the value *θ_T_* = 2.6×10^-3^ that separates the two populations with 95% and 98% accuracy, respectively.

(**B**) Distribution of *τ* for different values of *k* without (gray) or with bortezomib (green) following treatment with 5ng/ml of TRAIL (left) or 10nM of Mapatumumab (right) in HeLa ICRP cells. The red line is the fate boundary calculated with EQ3.

(**C**) Western blot analysis of DR4 (left) and DR5 (right) expression levels after treatment with TRAIL (25 ng/ml) and bortezomib (100nM) at different times in HeLa ICRP Bcl-2-overexpressing cells.

## Figure S4: Bortezomib and the clustering antibody anti-Fc recover Apomab potency

(**A**) Survival fractions for different concentrations of Apomab without (light blue) or with the clustering antibody anti-Fc (dark blue), in HeLa ICRP cells. Data are shown as mean ± SEM.

(**B**) Mean of *log_10_(k)* for different concentrations of Apomab without (light blue) or with the clustering antibody anti-Fc (dark blue) in HeLa ICRP cells. Data are shown as mean ± SEM.

(**C**) TRAIL and Apomab-induced cell death measured across 9 cancer cell lines (dose response at 24 hr). Left panels: Half maximal cell death EC50 values for TRAIL, Apomab and clustered Apomab (+Fc). The shade intensity ranges from the most sensitive in black to resistant (EC_50_>0.01μM) in white. Right panels: Surviving fraction at 0.01μM for the same agonists. Note that we chose to show the EC50 values (instead of IC50), which represent the half-maximal effect for each cell line, because no cell line (except Colo205) could reach 50% cell death with the maximal dose of clustered Apomab.

## Figure S5: Quantitative measure of FLIP-S/FLIP-L -dependent inhibition of caspase-8 dynamics.

(**A**) Left panel: constructs for FLIP-L and FLIP-S tagged with mCherry at their C-terminus. Right panel: time-lapse microscopy setup showing acquisition of an mCherry image followed by a FRET time series to relate FLIP expression levels to caspase-8 activity over time in individual cells.

(**B**) Quantitative Western blotting for FLIP relative to recombinant FLIP (6 lanes on the left) and FLIP-mCherry-overexpressing ICRP HeLa cells sorted by FACS into 4 pools (4 lanes on the right: determined number of cells of low, medium, high and highest expression levels were loaded, corresponding to 7μg of protein). The absolute numbers of FLIP molecules per cell were then used to extrapolate the number of FLIP molecules in each cell of the microscopy experiments (see Methods). Only the analysis of the FLIP-L-mCherry-overexpressing cell line is shown.

(**C**) Correlation between FLIP-S (left panel) and FLIP-L levels (right panel) measured using their mCherry tags (see Methods) and the rate of caspase-8 activation, *k*. Spearman’s correlation coefficient and the corresponding p-value are displayed. Black dots denote dying cells, whereas surviving cells are in blue.

(**D**) Mean values for the surviving fraction (left panel) and for *τ* (right panel, zoomed in on early time points) plotted against the mean of *log_10_(k)* for the parental line (HeLa ICRP cells) after treatments with different doses of TRAIL (black), Apomab (blue) and Mapatumumab (red) with and without anti-Fc. The values for the FLIP-overexpressing cells treated with 250 and 500 ng/ml of TRAIL are also displayed in orange. The arrow indicates the FLIP-L-overexpressing cells treated with 250 ng/ml of TRAIL. The black line in the left panel is the logistic curve fitted to all treatments of the parental line (see Fig. 4A). The red line in the right panel is the fate boundary calculated with EQ3. Data are represented as mean ± SEM.

(**E**) Derivative of FRET ratio for surviving cells (left panels) and cells committing to apoptosis (middle panels) following treatment with 250 ng/ml of TRAIL as a function of FLIP expression (color coded) for the FLIP-L-overexpressing cells (upper panels) and the FLIP-S-overexpressing cells (lower panels). Cyan and black dots show the maximal value of the derivative (note that black dots also indicate cell death). The right panels show the distribution of maximal values of the derivative for both populations (dying cells in yellow, surviving cells in blue). The red line indicates the value *θ_T_* = 2.6 × 10^-3^ that separates the two populations with 91% accuracy for both FLIP-overexpressing lines.

## Figure S6: Bcl overexpression increases the threshold *θ*

(**A**) Mean of *log_10_(k)* for parental cells after treatment with 25 ng/ml of TRAIL with or without ABT-263. Data are represented as mean ± SEM.

(**B**) Median *τ* values for parental cells after treatment with 25 ng/ml of TRAIL with or without ABT-263. Data are represented as mean ± SEM.

(**C**) Derivative of FRET ratio for Bcl-XL-overexpressing cells treated with 250ng/ml of TRAIL. The dashed red line indicates *θ_T_ =* 2.6 × 10^-3^ as defined for the parental population (HeLa ICRP cells). Surviving cells are in blue; dying cells in yellow. Cyan and black dots show the maximal value of the derivative (note that black dots also indicate cell death).

## Supplementary Materials and Methods

## Synthesizing Apomab and Mapatumumab human IgG1.

Apomab and Mapatumumab human IgG1 were cloned using sequences from patents US2006/0088523A1 (Apomab) and US7348003B2 (Mapatumumab) into an in-house vector (pCEP4) and expressed transiently in Freestyle 293F cells (Invitrogen). The antibodies were purified by Protein A chromatography (MabSelect, GE Healthcare), dialyzed into PBS pH 7.4 and filter sterilized.

## High-throughput live-cell imaging and analysis

### Imaging Setup

Two thousand five hundred clonal HeLa cells stably expressing the FRET-based initiator caspase reporter (ICRP) were seeded into 96-well plates coated with rat-tail collagen I (BD, Franklin Lakes, NJ). Cells were imaged every 5 min for up to 24 hr in the live-cell chamber of an Operetta robotic microscope (Perkin Elmer, Waltham, MA) with a 10x objective (NA = 0.4) using filter configurations for CFP (Ex. 425-450 nm / Em. 460-500 nm) and CFP-YFP FRET (Ex. 425-450 nm / Em. 520-560 nm). In addition, for cells stably expressing the MOMP reporter (IMS-RP) or proteins of interest tagged with mCherry, mCherry was imaged (Ex. 550-600 nm / Em. 610-660 nm) throughout all experiments that used the MOMP reporter or at the beginning of the experiment when FLIP-L/FLIP-S or Bcl-2/Bcl-XL tagged with mCherry was present.

### Segmentation

For background subtraction, images *I*_raw_ were tiled into 64 blocks sized 170 by 128 pixels. We used the mode of the Gaussian-filtered intensity distribution in each tile as a measure of local background and reconstructed a full-resolution background image *I*_bg_ by bilinear interpolation over the tile modes. Given zero-mean background intensity in *I*_CFP_ = *I*_CFP_*,*_raw_ – *I*_CFP_*,*_bg_, the lower half of background intensity was wrapped over the upper half in the distribution of absolute intensities |*I*_CFP_|, such that the 20^th^ percentile *p*_20_ of |*I*| could be used as a robust estimate of background variability. At a threshold *t* = 3 *p*_20_, false-positive mask elements in *M*_t_ = (*I*_CFP_ > *t*) were sparse enough to be reliably suppressed by morphological erosion. Before erosion, however, we separated contacting cells by watershed segmentation of *I*_CFP_ after applying a wide Gaussian filter. The Gaussian filter was parameterized such that oversegmentation was rare. The combined final mask M for single-cell readouts was obtained from the threshold mask *M*_t_ and the watersheds *W* by eroding $M_{t}\wedge\neg W$.

### Intensity readouts

The FRET ratio I_CFP_ / I_FRET_ was extracted from each cell in each segmented frame. To reduce the influence of chromatic aberration and between-channel jitter, the background-subtracted FRET image *I*_FRET_ was aligned to the background-subtracted CFP image *I*_CFP_ with subpixel resolution by bilinear interpolation. The required shift was determined for every frame by quadratic peak interpolation of the cross-correlation coefficients between *I*_FRET_ and *I*_CFP_. The mask *M* determined from *I*_CFP_ was applied per cell to both *I*_CFP_ and the aligned *I*_FRET_, such that the FRET ratio could be calculated as the median of the pixel-wise ratio of intensities in the mask area. Due to erosion of $M_{t}\wedge\neg W$, thin protrusions of cells with larger relative error in *I*_FRET_ and *I*_CFP_ were excluded from the median of pixel-wise ratios. As a measure of expression level of mCherry-tagged proteins of interest (FLIP-S/FLIP-L, Bcl-2/Bcl-XL), the 80^th^ percentile in the mask area of the background-subtracted, aligned mCherry image was used.

### Tracking

In addition to intensity readouts, the mask centroid (*x*, *y*)*_i_*_,_*_f_* was determined for each cell *i* in every frame *f*. To overcome between-frame jitter, between-frame shifts *Δx* and *Δy* were determined by cross-correlation analysis of consecutive CFP images that were downsampled 4-fold. Those shifts, *Δx* and *Δy*, were applied to centroids (x, y)*_i_*_,_ *_f_* for establishing tracks between frame *f* and *f*+1. Cells *i* and *j* in two consecutive frames *f* and *f*+1 were connected if (x+*Δx*, y+*Δy*)*_i_*_,_*_f_* is the nearest position to (x, y)*_j_*_,_*_f_*_+1_ among all (x+*Δx*, y+*Δy*)*_f_* and (x, y)*_j_*_,_*_f + 1_* is the nearest position to (x+*Δx*, y+*Δy*)*_i_*_,_*_f_* among all (x, y)*_f_*_+1_.

### Evaluation of cell death and MOMP time

We based our evaluation of cell death times on changes in the cell morphology or, when the mitochondrial inter-membrane space reporter (IMS-RP) was co-expressed, on its intracellular distribution. Morphological variables indicative of cell rounding that we extracted for each cell in each frame were the “area” of the single-cell mask and an “edge metric” quantifying the contrast at the cell boundary: 4 line-scans of *I*_CFP_ rotated by 45° around the mask centroid were Gaussian-filtered, and the median of the corresponding 8 maximal intensity slopes in the 3-pixel proximity of the mask boundary were determined. Spread-out cells yielded shallow maximal slopes for this readout, whereas cell rounding resulted in a sharp increase in intensity slope at the mask boundary. In some experiments, the IMS-RP was co-expressed with the initiator caspase FRET probe ICRP. To detect MOMP, the position of the nucleus was estimated by finding within a single-cell mask the position of peak intensity in CFP fluorescence after filtering *I*_CFP_ with a wide Gaussian. This approach made use of the observation that ICRP is not excluded from the nucleus and that epifluorescence is integrated over the widest z-range in that place. With intact mitochondrial outer membranes, IMS-RP is excluded from the nuclear region. After MOMP, IMS-RP becomes ubiquitously distributed inside the cell, including the nuclear area. We found that sharp variations in all of these three variables were indicative of either cell division or apoptosis. If cells regained their original values in edge and area metrics within less than 4 hr, the event was classified as a division; otherwise it was classified as apoptosis. Loss of tracking within these 4 hr or incomplete recovery was treated as an undermined fate.

All cells in the fields of view were analyzed, and only some were discarded when tracking was lost before the end of the experiment or before cell death or when an event was not properly classified as either a cell division or a cell death (see above). With this stringent approach, adjusting the cutoffs for fate classification did not affect the results qualitatively. We validated our approach by two tests. First, in the conditions that had IMS-RP co-expressed, the agreement between the morphological assessment and calls based solely on the IMS-RP was 80 ± 2%, and the times of MOMP had a Pearson’s correlation of r = 98.8 ± 0.4 across 4 conditions with different doses of TRAIL. Second, among the cells that were classified as dying, we located the inflection point in the FRET ratio that indicates the sudden activation of effector caspases. To pinpoint this event, we monitored the discrepancy between the finite derivative of the FRET ratio and the derivative of the FRET ratio after smoothing over 7 frames. We defined the time of MOMP as 4 frames before this discrepancy exceeded twice its standard deviation.

## Cell panel screen

### Cell culture and treatments

H1993, H2170, H1703, Calu3, HT29, HCT116, Colo205, SKBR3, SKOV3, Ovcar8, Panc-1, ACHN, A498, DU145, Jurkat and RPMI 8226 were obtained from ATCC (Manassas, VA) and cultured according to ATCC instructions. For dose response to rhTRAIL, cells were treated with a 10-point dilution starting at 10nM, with 3-fold dilutions. For dose responses to Apomab and Mapatumumab, with and without anti-Fc clustering, cells were treated with a 10-point, 4-fold dilution series starting at either 100nM or 20nM. When anti-Fc clustering was used, an anti-human IgG, Fc-fragment specific antibody (Jackson ImmunoResearch Laboratories, Inc.) was added to either Apomab or Mapatumumab at a 1:1 ratio. For time-response, 0.5, 1, 2, 4, 8 and 24 hr-time points were used.

***Death Receptor profiling by quantitative FACS***

DR4 and DR5 amounts were quantified with Quantum Simply Cellular anti-mouse IgG bead standards (Bangs Laboratories, 815) and anti–human IgG bead standards (Bangs Laboratories, 816), respectively. Cells and beads were stained with either an antibody against DR4 (Abcam, ab55863) or an Alexa 647–labeled antibody against DR5, Apomab. Both beads and cells stained with labeled IgG antibodies were analyzed by flow cytometry. Four populations of beads with different antibody-binding capacities were used as standards to quantify the number of surface receptors per cell according to manufacturer protocols.

***Cell viability assay***

Cells were plated in 96-well plates at 1 x 10^4^ cells/well overnight and then treated for 0.5, 1, 2, 4, 8, 24 hr. At the end of treatment, cell viability was assayed using CellTiter-Glo (Promega) according to the manufacturer’s protocol.

### C8 activity assay

Cells were plated in 96-well plates at 1 x 10^4^ cells/well overnight and then treated for 0.5, 1, 2, 4, 8, 24 hr. At the end of treatment, Caspase-8 activity was assayed using Caspase-Glo 8 (Promega) according to the manufacturer’s protocol.

***Analysis***

For the cell death and C8 activity assays, the readout of each well with cells was background subtracted based on a well with only media. Values for cell death and C8 activity assays were normalized by the untreated well to obtain the fraction of surviving cells and the C8 fold change, respectively.
