# Supplementary material for: Fractional killing arises from cell-to-cell variability in overcoming a caspase activity threshold
Source: Mol Syst Biol. 2015 May 7;11(5):803. doi: 10.15252/msb.20145584 (PMC4461398; doi:10.15252/msb.20145584)
Supplement: Supplementary file 8 [file msb0011-0803-sd8.pdf]

molecular systems biology

Fractional killing arises from cell-to-cell variability in overcoming a caspase activity threshold

Jérémie Roux, Marc Hafner, Samuel Bandara, Joshua J. Sims, Diana Chai, Peter K. Sorger

Corresponding author: Jérémie Roux, Harvard Medical School

Review timeline:

Submission date:	15 July 2014
Editorial Decision:	15 August 2014
Revision received:	26 January 2015
Editorial Decision:	15 February 2015
Revision received:	17 March 2015
Accepted:	27 March 2015

Editor: Thomas Lemberger

Transaction Report:

(Note: With the exception of the correction of typographical or spelling errors that could be a source of ambiguity, letters and reports are not edited. The original formatting of letters and referee reports may not be reflected in this compilation.)

1st Editorial Decision 15 August 2014

Thank you again for submitting your work to Molecular Systems Biology. We have now heard back from the three referees who agreed to evaluate your manuscript. As you will see from the reports below, the referees find the general topic of the study potential interest but are rather circumspect with regard to some of the major conclusions. They raise indeed substantial concerns on your work, which should be convincingly addressed in a major revision of the present study.

Without repeating all the points listed in the reports below, the two major issues that should be solved are the following:

- it is essential to prove that the cleavage of the ICRP reporter is not influenced by apoptosis execution events at the time points used to collect the data. This is essential to exclude that the reported measurements are reflecting the outcome of the decision process rather than predicting it (see comments of reviewer #3).

- the generality of the approach should be substantiated with the analysis of additional relevant cell lines. This is particularly important to better understand the lack of efficiency of Apomab, which should be analyzed beyond the sole HeLa cell line (see comments and suggestions of reviewer #1 and #3).

On a more editorial level, we would also kindly ask you to inlcude in the submission the datasets resulting from the analysis of the high-throughput live cell imaging used in this study so that others can re-analyze or re-use them. These datasets should report single-cell level intensity readouts, FRET ratio, cell death scores, MOMP time as well as the computed k, tau and fit significance.

Reviewer #1:

The manuscript reports fractional killing by TRAIL receptor agonists, proteasome inhibitors, therapeutic antibodies, and overexpression of resistance genes. A simple phenomenological model is established for TRAIL-induced activation of C8. The parameters of the model are determined based on high throughput live cell imaging utilizing a previously established FRET sensor. These studies facilitate the definition of a threshold as minimum instantaneous caspase-8 activity for individual cells to die upon exposure to TRAIL. With increasing doses of TRAIL, the rate of caspase activation k increases, thus more cells overcome the threshold and die. To a lower extent, the cell death fraction is also rising with higher doses of multivalent Mapatumumab. These findings are validated with Apomab, a therapeutic antibody with high affinity but low potency. A combination of the drug with a clustering agent and a proteasome inhibitor is sufficient to significantly reduce the surviving fraction. The study showcases how a phenomenological model can yield insights into single-cell based life-death decisions in the context of anti-DR4/5 therapeutic antibodies. However, since all studies have been exclusively performed with Hela cells, it remains to be shown how relevant these observations are in general.

Major comments:

1) It is known that cell lines are notoriously genetically instable. What is the basis (experimental observations / criteria) for stating in the abstract that the cell population is "homogeneous"?

2) The graphical representation of the mathematical model is not very intuitive - t0, t and k need to be better indicated. Further, it is stated on p7 that "surviving cells falling to the lower left of the fate boundary and dead cells to the upper right of the boundary". Yet in the landscape plot shown in Fig. 1D the majority of dead cells reside in the right lower part and this population increases upon increasing TNF concentrations.

3) As proteasome inhibition fundamentally alters the dynamics of C8 activation, the landscape plot for bortezomib alone should be displayed in addition. In the text "treatment with bortezomib" is mentioned but apparently these statements always refer to co-treatment conditions.

4) Fig. 4A needs to be clarified: How was the SEM of k determined in Fig. 4A? Why should +Fc also reduce as indicated by the second black arrow in Fig. 4C?
5) A major conclusion is that Apomab "failed as a drug because it fell below the threshold for both the rate and the duration of C8 activation" as indicated in the results part (p12) and in the abstract it is stated that "the threshold helps explain why therapeutic antibodies as Apomab failed". The results obtained in Hela cells certainly provide a possible explanation, but others are also conceivable. Pro-apoptotic effects of Apomab have been observed in the context of preclinical models. It was shown that e.g. breast cancer cell lines differ in their sensitivity towards Apomab (Zinonos et al, Mol Can Ther 2009). It would be of major interest to observe whether in these cells the threshold is shifted or whether the rate of C8 activation is less decisive for death decisions.
Before broader conclusions can be drawn, general applicability has to be demonstrated.

6) In a second part of the story the authors suggest that "proteasome inhibition decreased k in FLIP-S and FLIP-L over-expressers (Fig. 6D), an anti-apoptotic change counteracting pro-apoptotic increase in ." As correctly stated, FLIP-S and FLIP-L are competing with C8 for recruitment to the DISC, thereby acting as inhibitors. Decrease in k for FLIP-S and FLIP-L over-expression can be directly explained by this negative effect. Further reduction of k in the presence of bortezomib is more likely to be attributed to longer half-life of FLIP-S and FLIP-L as compared to C8. An enhanced negative correlation between and k could be an additional indication for this hypothesis.

7) The experimental data depicted in Fig. 7 should also be shown in the /k landscape to compare to the boundary changes indicated in the graphical representation in Fig. 7E. Instead of displaying the results for Bcl-XL in Fig. 7B it would be more congruent to show the results for Bcl2. Is it possible

that the threshold also shifts under physiological conditions with respect to intrinsic noise of FLIP-L, FLIP-S, Bcl-2, Bcl-XL?

Minor comments:
1) Optimization procedure for "Trajectories fitting" and "Determination of the value " in the "Expanded View: Materials and Methods" as well as the Heavyside function need more explanation.

2) The abbreviation SVN should be explained in the Figure legend. The color code used for SVN is inconsistently applied in the Expanded View.

3) Please unify the terms "cell death fraction" and "surviving fraction" in the text and figure legends.

4) Bands for the processed IC-RP in Fig. E1A should also be shown.

Reviewer #2:

Comments:
Caspase-8 is a key molecule in the death receptor (DR)-mediated extrinsic apoptotic pathway, and is activated in the death-inducing signaling complex (DISC) with DRs. Therefore, to investigate the caspase-8 activation profile leads to understanding whether cells subjected to extrinsic apoptotic stimuli are destined to die or still stay alive. The authors challenged to clarify this cell fate determination by setting up a threshold in the rate and timing of caspase-8 activation based on the mathematical model. The authors construct a simple model for the caspase activation. Caspase-8 activation is described as a function of time. Time derivative of FRET ratio representing the cumulative activated caspase-8 designated by dFR/dt reaches maximum value at time . Threshold of dFR/dt designated by appropriately divides apoptotic into survival cell. Variation of is mainly caused by the degradation of caspase-8. In this study, the authors examined the killing effects of a natural TRAIL ligand and its agonistic antibodies use as an apoptotic stimulant. Furthermore, the authors analyzed the threshold in apoptosis-resistant cells, which were overexpressed a regulator, c-FLIP or anti-apoptotic Bcl-2 members, Bcl-2 and Bcl-XL, and in stimulation-sensitive cells by treatment with a proteasome inhibitor. These results are reliable and noteworthy based on vast data and due to steady progression, and clearly demonstrated the existence of a threshold on caspase-8 activation in extrinsic apoptotic signaling. This study will provide valuable information and novel insights into the molecular mechanism to understand the cell-to-cell variability. However, the present manuscript does not fully correspond to several issues as described below. Therefore, more work will be required for revision.

Major points:
1. The authors have not shown any data with regard to the expression levels of DR4 and DR5 in HeLa cells in this study. However, the experimental confirmation of these receptor proteins is an essential issue to figure out the action of TRAIL for DR4/5, agonistic Mapatumumab for DR4, and Apomab for DR5. In the previous report (Laussmann, M.A. et al., JBC, 2012), it has been shown that DR5, but not DR4, is accumulated by Bortezomib treatment. The authors should also examine the amounts of both DR4 and DR5 proteins before and after proteasome inhibition. The result from this performance may lead to a distinct conclusion in the third chapter "C8 trajectories and ... degradation" of the Result section.

2. As shown in more recent report (Graves, J.D. et al., Cancer Cell, 2014), the additional experiments using combination of TRAIL and agonistic antibody Mapatumumab or Apomab are meaningful to find the maximal cytocidal effect.

3. In the fifth chapter "Differentiation effects ... and cell killing", the authors investigated the function of c-FLIP isoforms, c-FLIP_L and c-FLIP_S as a negative regulator. The authors confirmed the expression levels of fusion proteins, c-FLIP_L/mCherry and c-FLIP_S/mCherry in transfected cells by immunoblot analysis, and showed the data in Fig. E5B. In the figure, a peptide band corresponding to an ectopically expressed fusion protein was observed but an endogenous c-FLIP_L was not. In contrast, we can see the significant bands of endogenous c-FLIP_L and c-FLIP_S proteins in parental cells by immunoblot analysis (Fig. 6C). As these data were inconsistent, a fresh investigation is required to clarify the differential expression levels of ectopic and

endogenous c-FLIP molecules. In addition, it is difficult to understand the description and data shown in Fig. 6B, indicating that c-FLIP_L/mCherry-expressing transfected cells become re-sensitive to TRAIL-induced apoptotic stimuli in the presence of Bortezomib. Therefore, it is recommendable to address the fluctuation of a c-FLIP_L/mCherry fusion protein by immunoblot analysis using an anti-mCherry antibody.

Reviewer #3:

The authors submitted a manuscript in which they describe an equation that is supposed to define a threshold for apoptotic cell death in HeLa cells treated with death receptor ligand TRAIL or receptor activating antibodies, alone or in co-treatments with a proteasome inhibitor or with Fc-gamma-directed antibodies. The equation has appeal due to its simplicity, and it can be arranged to calculate the threshold "theta" in dependence of the caspase-8 activation rate (k) and the time of maximum activity (tau). Since "theta" performs well as a separator between dying and surviving subpopulations, individual cells which cross the theta threshold can be considered to have a higher likelihood to commit apoptosis. The separator threshold remains valid for different perturbations for HeLa cells. Due to its nature, the equation therefore does not predict % fractional killing at the population level for a given condition but rather highlights that caspase-8 activation rates and times of maximum activity for individual cells can indicate whether these cells will die or not. If correct, this is an interesting observation that extends beyond previous analysis of activation rates in HeLa cells by the authors (Nature 459, 428-432, 21 May 2009).

However, I have major reservations regarding this study, as described below.

Major comments:

1. I would like to raise a major concern regarding the fundamental statement of the paper (the claim that rate of casp-8 activation and time of maximal activity, Tau as determined from max dFR/dt, are predicting death). In my understanding, the authors used a previously described FRET probe for their experiments, and this probe can be cleaved by caspase-8 and even more effectively by executioner caspases. They previously showed that the rate of probe cleavage dramatically increases during apoptosis execution. This could be misinterpreted as high rates of casp-8 activation (k) and consequently shorter times needed to reach max activity (tau). I understand that the authors either used morphological changes or the release of a mitochondrial protein as readouts of cell death or apoptosis execution, but there is no direct proof that cleavage of the probe during the execution phase does not contribute to the analysis (i.e. analysis must be performed on data collected clearly before the event of mitochondrial permeabilization starts). Such a proof is of fundamental importance, since otherwise theta merely separates dying and surviving cells based on activation of apoptosis execution and therefore would not be predictive at all (i.e. signalling during the upstream phase would not predict whether cells will die or not, using the theta threshold). This concern is further substantiated by data presented for conditions of low TRAIL concentrations. Here, signalling takes longer and the biphasic nature of the probe cleavage, as described by the group in a previous study, seems very apparent. For example, in Figure E3A (upper right) the dying cells show low dFR/dt values for prolonged times, and these dramatically increase before cells die, probably indicating the effect of executioner caspases. A number of additional comments related to this major comment are listed below.

2. The authors should better clarify how their equation/model and its value differs from their previous, similar analysis (Nature 459, 428-432, 21 May 2009) where the group investigated rates of casp-8 activation and a threshold "theta" in the context of cell death timing (see e.g. Fig.3).

3. The practical value of the equation to me appears to be overstated. Statements such as in the abstract ("the threshold helps explain why therapeutic antibodies failed in phase II..."). If the antibody failure is due to lack of efficient caspase-8 activation, this could be shown much easier by classical immunoblotting (Apomab, by the way, has been shown to efficiently kill various cell lines. Lack of potency here may be HeLa specific)? Likewise, it is unclear to me how the evaluation of "new TRAIL-like drugs" would benefit from use of the equation together with imaging experiments over classical experimental approaches and how this could "rescue a once-promising therapeutic class". Unless I am missing the point, I suggest that the authors use a considerably more modest

narrative, not only in the abstract.

In the following I have provided a list of additional important comments, some of which are related to the major comments above.

1. Throughout, the authors should be more specific in their description of the experimental data. For example, results and figure legend related to Fig.1 do not mention which cell line has been used. Information on the number of repeat experiments should be provided. Information on the times of cell death/survival readings should be provided etc.

2. On Page 4, it should probably read "processing of ICAD" rather than "ICAD nucleases". Also, XIAP does not bind the catalytic pocket of caspase-9 (see Srinivasula et al, Nature 2001).

3. Can the authors clarify whether all cells in the fields of view were analysed? In Fig.1A it seems that all cells respond with FRET ratio changes. Does this mean that non-responders do not exist at the level of caspase-8 activation?

4. This comment is related to major comment 1 and the potential problem of having the execution phase contributing to the signals. When using HeLa cells overexpressing Bcl-2 or Bcl-xL, would cells with dFR/dt values above the red line be observed with the same frequency as in the wt cell population for a treatment condition as in Fig.1B (25 ng/ml TRAIL)? This would be a very important control (Fig.7B in this context cannot serve as a control due to the 10-fold higher TRAIL concentration).

5. Fig.E1A would benefit from also showing the signal for the cleaved probe as well as a loading control. Currently it looks like no probe is cleaved at all when overexpressing Bcl-2. Loading controls for other immunoblots should also be included.

6. End of page 9, start of page 10: It is well described that the amounts of tBid do not need to exceed molar amounts of Bcl-2 and Bcl-xL. The associated reference from 1993 is outdated. Bcl-2 family signalling itself is non-linear, and small nanomolar amounts of tBid are sufficient to induce MOMP. Also, tBid is a very short lived protein and its stability may very well differ from that of the cleaved probe. The amount of tBid present at any one time may therefore differ considerably from the amount of cleaved probe. It therefore seems logical to me that the rate of probe cleavage (i.e. the flux of tBid generation) rather than the final amount of cleaved probe is associated with the life/death decision.

7. I consider a concentration of 100 nM bortezomib as very high. While this may not cause death within 24 h (not shown), it will surely do so at later times. The authors therefore need to provide information on when survival in their experiments was determined. Also, they will need to clarify that their readouts and modelling only relates to early death through the extrinsic pathway. Furthermore, if bortezomib itself may kill at high rates at later times, the co-treatment scenarios with TRAIL would only reflect a shift of death to earlier times. Would that not beat the purpose of evaluating or optimising ("rescuing") TRAIL receptor-based treatments, since the other drug alone is already sufficiently potent and TRAIL would not be needed?

8. DR5 is known to rapidly accumulate following proteasome inhibition, and it would be expected to enhance DISC formation and casp-8 activation. Fig.3C indicates that Tau increases whereas k may not be affected. Would that not be counter-intuitive (unless cFLIP levels, which may accumulate as well, cause the delay)? Would such a DR5 upregulation not also (partially) explain the lack of protection through cFLIP-L overexpression in Fig.6A (cFLIP as an enhancer of death)? A reductionist view explaining the effect observed in Fig.3 only by casp-8 stability does not seem to be warranted without comprehensive biochemical analyses.

9. For the TRAIL 5 ng/ml + Bortezomib treatment data shown in Fig.3C and Fig.E3A there seems to be a discrepancy. In the main paper, most dying cells being placed to the left of the separator line (what is the accuracy here?), while the separation in Fig.E3A appears to be much better (accuracy here 95%). I would expect both accuracies to be equivalent, since the same data are presented?!

10. The authors use the terms "prediction", "classification" and also refer to "accuracy" of the

separations. Are these terms interchangeable? Could the authors clarify this, please? In my understanding of the main paper, data for TRAIL 25 ng/ml was used to define the equation, and theta was then used as a separator (predictor?) for all other treatment conditions? In contrast to this, in the supplemental methods instead it says that "theta" was determined from ALL experiments with TRAIL > 10 ng/ml (supplement, page 8). If this is true, the main manuscript is misleading since the equation technically would be a fit across all data rather than a threshold that was subsequently validated by independent data.

11. In the discussion (page 17), the authors refer to changes in factors regulating NFkB. A major point would be the widely acknowledged prevention of IkBalpha degradation.

12. Trajectory fitting and Theta determination are very central to the paper, and it would be better to include these methods in the main document.

Independent of the above comments I would like to bring the following to the authors' attention. Recombinant human Trail, as bought from vendors, contains small fractions of protein aggregates (typically in the low % range of the total protein content). When removing these aggregates by gel filtration, rhTRAIL preparations typically do not kill. This is often overlooked when discussing the potency of receptor agonists and ligands.

1st Revision - authors' response 26 January 2015

Detailed Response to Review of MSB-14-5584R:

Points raised by the editor are addressed in the cover letter including:

1) it is essential to prove that the cleavage of the ICRP reporter is not influenced by apoptosis execution events at the time points used to collect the data.

2) the generality of the approach should be substantiated with the analysis of additional relevant cell lines.

3) include in the submission the datasets resulting from the analysis of the high-throughput live cell imaging used in this study

Reviewer #1 Major Comments:

1) It is known that cell lines are notoriously genetically instable. What is the basis (experimental observations / criteria) for stating in the abstract that the cell population is "homogeneous"?

RESPONSE: By homogenous we mean that cells are exposed to similar factors in the environment and are freshly derived from single-cells (by subcloning). We mean this as a less precise term than "isogenic" because cell lines are indeed unstable, as the reviewer correctly points out. The fact that the probability and timing of cell death observed in parental cell populations and in cells that survive TRAIL exposure (and are then regrown) are indistinguishable demonstrates that genetic variation has a minimal impact on the cell-to-cell differences (e.g. (*Spencer et al. Nature 2009 PMID 19363473, Flusberg et al. Mol Biol Cell 2013 PMID: 23699397*). Nonetheless, we have rewritten the abstract without the term "homogeneous" (page 2 paragraph 1) and have also edited our Methods section on page 22, paragraph 1.

2) The graphical representation of the mathematical model is not very intuitive – t0, t and k need to be better indicated. Further, it is stated on p7 that "surviving cells falling to the lower left of the fate

© European Molecular Biology Organization

boundary and dead cells to the upper right of the boundary". Yet in the landscape plot shown in Fig. 1D the majority of dead cells reside in the right lower part and this population increases upon increasing TNF concentrations.

RESPONSE: As suggested by the reviewer, we made changes to Fig. 1C designed to clarify the representation of the model (t_0, t and k are now shown on the graphical representation of a typical ICRP cleavage trajectory).

The boundary between living and dying cells typically cuts the landscape of τ and k diagonally and 'upper right' therefore is meant to represent a position either above or to the right of the boundary. As the reviewer pointed out, since most of the dead cells die early (that is, they have a low value for tau) they actually fall to the right of the fate boundary but in the lower right quadrant. We apologize for this confusion (not having considered this interpretation previously) and have therefore changed the description of the figure to read "surviving cells falling to the left of the fate boundary (low k and/or short τ, in blue) and dead cells to the right of the boundary (higher k and/or longer τ, in yellow; Fig. 1D)" as suggested.

We edited Figure 1C and the results section in page 8, paragraph 3.

3) As proteasome inhibition fundamentally alters the dynamics of C8 activation, the landscape plot for bortezomib alone should be displayed in addition. In the text "treatment with bortezomib" is mentioned but apparently these statements always refer to co-treatment conditions.

RESPONSE: We found that exposure of HeLa cells to bortezomib at the concentrations and times used in this study does not trigger measurable C8 activation (or cell death) and it is therefore impossible to extract the parameters k and τ needed for plotting a landscape. To illustrate this point, we added as a supplemental figure (Fig. E3A) from an experiment with bortezomib alone. As correctly pointed out by the reviewer, we changed the main text to "in combination with TRAIL" or "co-treatment" for better clarity.

We edited Figure E3A and the results section in the 2nd paragraphs of pages 11 and 12.

4a) Fig. 4A needs to be clarified: How was the SEM of k determined in Fig. 4A?

RESPONSE: The SEM was determined using the formula *SEM=s/sqrt(n)* where s is the corrected sample standard deviation and n the number of experiments for a given agonist/dose. The value of k is the mean of the *log10* of individual k across all cells in an experiment (the geometric mean). Most conditions have at least three independent biological replicates although some conditions with intermediate doses were only performed in duplicates (in that case, the SEM is the difference between the two values). To clarify how many replicates were used for each calculation, we have added a supplementary table that summarizes all experiments and reports the mean values for k, tau, surviving cell fraction and classification accuracy.

We edited the legend for Figure 4A (page 32) and added a supplementary table.

4b) Why should +Fc also reduce τ as indicated by the second black arrow in Fig. 4C?

RESPONSE: Since Fc increases caspase-8 activity in cells treated with agonist antibodies, maximum C8 activity also occurs earlier; a similar effect was observed for TRAIL and reflects the fact that τ and k are not entirely independent of each other (see description of Figure 1E: "*The distribution of tau also became tighter, simply because tau cannot be longer than the time between ligand addition and death*". However since the increase in cell death due to +Fc is very limited, we removed the second black arrow for clarity.

We also edited Figure 4C to clarify this point.

5) A major conclusion is that Apomab "failed as a drug because it fell below the threshold for both the rate and the duration of C8 activation" as indicated in the results part (p12) and in the abstract it is stated that "the threshold helps explain why therapeutic antibodies as Apomab failed". The results obtained in Hela cells certainly provide a possible explanation, but others are also

conceivable. Pro-apoptotic effects of Apomab have been observed in the context of preclinical models. It was shown that e.g. breast cancer cell lines differ in their sensitivity towards Apomab (Zinonos et al, Mol Can Ther 2009). It would be of major interest to observe whether in these cells the threshold is shifted or whether the rate of C8 activation is less decisive for death decisions. Before broader conclusions can be drawn, general applicability has to be demonstrated.

RESPONSE: Our response to this critique has three parts. First, we apologize for overstating our conclusion – in search of clarity of exposition we did not adequately express the necessarily speculative nature of our conclusions about future therapeutic agents. We have addressed this by re-writing p12 and related parts of the manuscript (see below).

Second, we have added new experiments in which Apomab and TRAIL were compared across a panel of cell lines sensitive to TRAIL. Consistent with the data in Zinonos et al, we find that Apomab is significantly less potent as an inducer of cell death than TRAIL in most if not all cell lines, and that the majority of lines are relatively Apomab resistance (as shown in Figure E4C of the revised manuscript). The limited caspase-8 activation induced by Apomab is sufficient to explain this lack of potency and we therefore believe that our (more modest) conclusions are supported by the data.

Third, we have performed a preliminary characterization of C8 dynamics in a panel of 9 additional cancer cell lines and concluded that the changes we engineered into HeLa cells, namely Bcl-2 and FLIP over-expression recapitulate the differences observed in these newly analyzed cells. TRAIL was chosen for these experiments over Apomab, because of the wider range of cellular response and its greater potency. This provides a preliminary assessment of the reviewers concern "*whether the rate of C8 activation is less decisive for death decisions"* in other cell lines: data obtained in 8/9 lines support this observation made in parental and engineered HeLa cells and thus our conclusion that analyzing features of receptor-mediated apoptosis is of general significance. We admit, however, that these new experiments are not entirely definitive since they do not have the precision afforded by live-cell reporters in genetically engineered cells. Unfortunately, performing live-cell experiments on multiple engineered cell lines will take quite a while and goes beyond the scope of the current study. We believe that the available new data, in combination with our extensive re-writing, address the reviewer's primary concerns. The results of experiments performed in cells other than HeLa are now described in a new figure (Figure 8).

The manuscript has been edited on page 2, on page 12 paragraph 3 and on page 13 (abstract and results on the Apomab's failure as a drug). We added the results and conclusions of our new set of experiments: on page 13 with a new figure panel (Figure E4C) and on page 17 paragraph 2 with a new figure (Figure 8). We added the corresponding methods to the Supplemental Materials (Expanded views). These edits also address comment 3 from reviewer 3.

6) In a second part of the story the authors suggest that "proteasome inhibition decreased k in FLIP-S and FLIP-L over-expressers (Fig. 6D), an anti-apoptotic change counteracting pro-apoptotic increase in τ." As correctly stated, FLIP-S and FLIP-L are competing with C8 for recruitment to the DISC, thereby acting as inhibitors. Decrease in k for FLIP-S and FLIP-L over-expression can be directly explained by this negative effect. Further reduction of k in the presence of bortezomib is more likely to be attributed to longer half-life of FLIP-S and FLIP-L as compared to C8. An enhanced negative correlation between τ and k could be an additional indication for this hypothesis

RESPONSE: We thank the reviewer for clarifying this point. Although we did not find an enhanced negative correlation between τ and k, we agree with the reviewer that "the further reduction of k in the presence of bortezomib is likely due to the longer half-lives of FLIP-S and FLIP-L as compared to caspase-8." We have therefore edited the results section in pages 15-16 to reflect this point.

7a) The experimental data depicted in Fig. 7 should also be shown in the τ/k landscape to compare to the boundary changes indicated in the graphical representation in Fig. 7E. Instead of displaying the results for Bcl-XL in Fig. 7B it would be more congruent to show the results for Bcl2.

RESPONSE: Figure 7 was edited to reflect these suggestions. The figure now contains two new panels: a τ/k landscape for the parental cells treated with ABT to compare the boundary changes

(Fig. 7B), and a *τ/k* landscape for Bcl-2 overexpressers (Fig. 7E). The *dFR/dt* traces for Bcl-XL over-expressing cells were moved to supplemental Figure E6C and replaced by traces for Bcl-2 over-expressers (Fig. 7D). The supplemental figure E6 also contains two new panels: showing parameters of caspase-8 trajectories in cells co-treated with ABT (E6A,B).

We also edited the results section (page 16 paragraph 2), the figure legends (pages 34-35 and Expanded view page 4) to reflect these changes.

7b) Is it possible that the threshold also shifts under physiological conditions with respect to intrinsic noise of FLIP-L, FLIP-S, Bcl-2, Bcl-XL?

RESPONSE: Yes: we expect that in addition to changes in C8 trajectories and fractional cell killing caused by changes in the levels of proteins that we measure or manipulate directly, extrinsic noise in other factors (e.g. Bcl-2, Bcl-XL, Mcl-1) will have an effect on the threshold. Such variation is very probably the reason why we cannot obtain a classifier based on measurement of C8 that has a precision above 80%. We mentioned this fact in the results page 8 paragraph 1: "*... random fluctuations in the activities and levels of proteins functioning downstream of the DISC (e.g. regulators of MOMP or effector caspase, etc.) have previously been shown to contribute ~20% to variability in the timing of cell death (Gaudet et al., 2012).*" We also refer to this idea in the Discussion on page 18 paragraph 1.

Reviewer #1 Minor comments:

1) Optimization procedure for "Trajectories fitting" and "Determination of the value θ" in the "Expanded View: Materials and Methods" as well as the Heavyside function need more explanation.

RESPONSE: A more detailed methodology has been added for all 3 points and moved to the main manuscript as suggested by reviewer 3 (additional comment #12).

2) The abbreviation SVN should be explained in the Figure legend. The color code used for SVN is inconsistently applied in the Expanded View.

RESPONSE: The abbreviation SVM (support vector machine) is now explained in all figure legends (in main manuscript on page 30 and in Expanded views on page 1), and the color for the SVM line (black) has been corrected in Fig. E1B.

3) Please unify the terms "cell death fraction" and "surviving fraction" in the text and figure legends.

RESPONSE: We have chosen "surviving fraction" to replace "cell death fraction" in all figures, thereby making them consistent.

4) Bands for the processed IC-RP in Fig. E1A should also be shown.

RESPONSE: The bands of the processed IC-RP are now shown in Fig. E1A.

Reviewer #2 Major Comments:

1) The authors have not shown any data with regard to the expression levels of DR4 and DR5 in HeLa cells in this study. However, the experimental confirmation of these receptor proteins is an essential issue to figure out the action of TRAIL for DR4/5, agonistic Mapatumumab for DR4, and Apomab for DR5. In the previous report (Laussmann, M.A. et al., JBC, 2012), it has been shown that DR5, but not DR4, is accumulated by Bortezomib treatment. The authors should also examine the amounts of both DR4 and DR5 proteins before and after proteasome inhibition. The result from

this performance may lead to a distinct conclusion in the third chapter "C8 trajectories and ... degradation" of the Result section.

RESPONSE: We would like to thank the reviewer for reminding us about the role of protein degradation in determining receptor abundance. In response, we have now measured DR4 and DR5 protein expression levels by western blotting in the clone of HeLa cells that carries the caspase-8 reporter (ICRP) used throughout the paper. As previously observed by Laussmann et al. (Laussmann, M.A. et al., JBC, 2012), we did not observe an increase in DR4 upon bortezomib treatment and DR5 levels rose modestly only 6 hours after bortezomib addition. Since we apply bortezomib and death agonists simultaneously, the current analysis is concerned with the period of time up to maximal caspase-8 activity which is typically ~ 3hr. Thus, the effects of long-term bortezomib exposure on DR5 levels are unlikely to have a major impact on the early caspase-8 dynamics analyzed here. We have added this data to the result section to complement our findings on the effect of bortezomib on TRAIL-dependent caspase activity (page 12 paragraph 2), and a new figure panel (Figure E3C).

2) As shown in more recent report (Graves, J.D. et al., Cancer Cell, 2014), the additional experiments using combination of TRAIL and agonistic antibody Mapatumumab or Apomab are meaningful to find the maximal cytocidal effect.

RESPONSE: As the reviewer correctly points out, two studies have shown that Conatumumab (AMG655) synergies with TRAIL (Tuthill MH et al., Oncogene 2014 and Graves, J.D. et al., Cancer Cell, 2014). However this interaction is likely to be antibody-specific and we have not yet managed to source AMG655 to perform additional experiments with this agonist. The Graves' study showed that the synergy was due to the formation of higher receptor clusters. There is no published data reporting synergy between Mapatumumab or Apomab and TRAIL, and our data did not reveal such an effect either.

3) In the fifth chapter "Differentiation effects ... and cell killing", the authors investigated the function of c-FLIP isoforms, c-FLIP_L and c-FLIP_S as a negative regulator. The authors confirmed the expression levels of fusion proteins, c-FLIP_L/mCherry and c-FLIP_S/mCherry in transfected cells by immunoblot analysis, and showed the data in Fig. E5B. In the figure, a peptide band corresponding to an ectopically expressed fusion protein was observed but an endogenous c-FLIP_L was not. In contrast, we can see the significant bands of endogenous c-FLIP_L and c-FLIP_S proteins in parental cells by immunoblot analysis (Fig. 6C). As these data were inconsistent, a fresh investigation is required to clarify the differential expression levels of ectopic and endogenous c-FLIP molecules. In addition, it is difficult to understand the description and data shown in Fig. 6B, indicating that c-FLIP_L/mCherry-expressing transfected cells become re-sensitive to TRAIL-induced apoptotic stimuli in the presence of Bortezomib. Therefore, it is recommendable to address the fluctuation of a c-FLIP_L/mCherry fusion protein by immunoblot analysis using an anti-mCherry antibody.

RESPONSE: We would like to thank the reviewer for raising these concerns – they reflect inadequate description of the methodology on our part. The immunoblot shown in Fig. E5B is different from all other immunoblots of the study since it represents an attempt to quantify protein levels (c-FLIP_L/mCherry) against a FLIP recombinant standard. Samples were prepared by lysing a known number of cells recovered from a FACS sorter (7µg of total protein). This is ~7-fold less than the amount of total protein (and thus, number of cells) than what was loaded in all the other immunoblots (50µg of protein as stated in the Methods). Thus, endogenous FLIP is not detectable in E5B, in contrast to the western in Fig. 6C. We now describe the difference in protein loading in the legend of Fig. E5 and make clear this has ca 1/7 the amount of total cellular protein per lanes as other Western blots.

To further clarify this point we also changed the description of Fig. 6B, as suggested by the reviewer, to better explain that cells over-expressing FLIP-L became re-sensitized to TRAIL-induced apoptotic stimuli in the presence of bortezomib (Fig. 6B). This occurs because the observed increase in τ was sufficient to push these cells, in which k also decreased, over the cell fate boundary (Fig. 6A).

Overall, the changes affect the legends for Figure E5 on pages 3-4 of the Expanded view and the result section pages 14-16.

Reviewer #3 Major comments:

1) I would like to raise a major concern regarding the fundamental statement of the paper (the claim that rate of casp-8 activation and time of maximal activity, Tau as determined from max dFR/dt, are predicting death). In my understanding, the authors used a previously described FRET probe for their experiments, and this probe can be cleaved by caspase-8 and even more effectively by executioner caspases. They previously showed that the rate of probe cleavage dramatically increases during apoptosis execution. This could be misinterpreted as high rates of casp-8 activation (k) and consequently shorter times needed to reach max activity (tau). I understand that the authors either used morphological changes or the release of a mitochondrial protein as readouts of cell death or apoptosis execution, but there is no direct proof that cleavage of the probe during the execution phase does not contribute to the analysis (i.e. analysis must be performed on data collected clearly before the event of mitochondrial permeabilization starts). Such a proof is of fundamental importance, since otherwise theta merely separates dying and surviving cells based on activation of apoptosis execution and therefore would not be predictive at all (i.e. 11ignaling during the upstream phase would not predict whether cells will die or not, using the theta threshold). This concern is further substantiated by data presented for conditions of low TRAIL concentrations. Here, signaling takes longer and the biphasic nature of the probe cleavage, as described by the group in a previous study, seems very apparent. For example, in Figure E3A (upper right) the dying cells show low dFR/dt values for prolonged times, and these dramatically increase before cells die, probably indicating the effect of executioner caspases. A number of additional comments related to this major comment are listed below.

RESPONSE: See also the response to editors remarks in the cover letter.

We would like to thank the reviewer for pointing out this concern since it is critical for our conclusions. One potential source of confusion is the different shape of raw FRET trajectories which we have previously published and their derivatives *dFR/dt* that we are using in our current study. As described below, we are confident that we are indeed monitoring Caspase-8 in the pre-MOMP period and that our conclusions are therefore valid.

First, as suggested in reviewer's additional comment #4, we measured caspase-8 trajectories in cells overexpressing Bcl-XL and exposed to 25ng/ml TRAIL. Under these conditions cells do not undergo MOMP and they do not die; all cleavage of ICRP is therefore due to the action of initiator caspases. We observed that the distribution of *k* values was similar between wt and Bcl-XL over-expressers (Fig. E1C) and that cells with *dFR/dt* values above θ_T were observed at the same frequency in the two populations of cells (Fig. E1B). This confirms that the measure of *Max(C8)* and the rate *k* is not influences by caspase-8 activation during the execution phase of cell death.

More generally, the reviewer is correct that we used translocation of the MOMP probe (IMS-RP) in the cytosol and cell morphology to determine cell death and truncate FRET trajectories prior to analysis. For technical reasons (the interval between two images, phototoxicity, and insufficient fluorescent channels for imaging engineered cell lines), we were not able to systematically monitor IMS-RP, ICRP_FRET and cherry-tagged protein levels and therefore relied on cell morphology as a measure of cell death. In a subset of experiments where both were measured, the agreement between cell death and the time of MOMP was 80 ± 2% (as stated in the first part of the results and described in more details in the Methods). We included this detail in the method section as suggested in the reviewer's minor comment #12.

To reflect these additions and changes we edited the results section on page 6 paragraphs 2 and 3 (to include the precision with which death is called) and in page 7 paragraph 4 and page 8 paragraphs 1 and 2 to present the results of the new experiment confirming that the cleavage of the ICRP reporter is not influenced by apoptosis execution events at the time points used to collect the data. New figure panels E1B,C were added.

© European Molecular Biology Organization

2) The authors should better clarify how their equation/model and its value differs from their previous, similar analysis (Nature 459, 428-432, 21 May 2009) where the group investigated rates of casp-8 activation and a threshold "theta" in the context of cell death timing (see e.g. Fig.3).

RESPONSE: In our previous analysis (Nature 459, 428-432, 21 May 2009 and other papers cited therein), HeLa cells were treated with TRAIL plus cycloheximide, causing all cells to die. The detailed biochemical model we assembled using this data was able to explain variability in the timing of cell death, but did not consider bifurcations in cell fate. Moreover, neither this model, nor one we subsequently developed in 2013 (*Lopez et al. Mol Syst Biol 2013 PMID 23423320*) accurately accounted for the observed kinetics of ICRP cleavage in cells that had not been treated with cycloheximide. The current study was initiated in an attempt to explicate the underlying biology and this necessarily involves a less detailed type of modeling. The phenomenological model we describe is conceptually analogous to the "*Idealized single-cell time course*" model presented in Fig 2c (Equation 1) of Albeck et al 2008, but with parameters related to DISC dynamics.

In the absence of cycloheximide, fractional killing is observed and we now report that ICRP trajectories have two phases in survivor cells (schematized in Fig. 1C). The goal of our phenomenological modeling is to determine how surviving and dying cells differ with respect to their kinetics and to evaluate the explanatory power of C8 trajectories in cell killing. Parameters in the phenomenological model can be interpreted: the parameter k is related to the activity of the DISC (we showed that the type of agonist, its concentration and the composition of the DISC (FLIP levels) directly affect the value of k); the parameter τ is related to the decay of caspase-8 activity (we showed that bortezomib can change its value). As discussed in the manuscript, the next step in this line of research is to develop a detailed mechanistic model of fate bifurcations that describes underlying reactions in molecular details. Such a model will build on our earlier work, but will require an as-yet unknown set of additional processes and factors.

We have edited the result section to clarify how the phenomenological model and its parameters differ from our previous work (page 9 paragraph 1).

3) The practical value of the equation to me appears to be overstated. Statements such as in the abstract ("the threshold helps explain why therapeutic antibodies failed in phase II..."). If the antibody failure is due to lack of efficient caspase-8 activation, this could be shown much easier by classical immunoblotting (Apomab, by the way, has been shown to efficiently kill various cell lines. Lack of potency here may be HeLa specific)? Likewise, it is unclear to me how the evaluation of "new TRAIL-like drugs" would benefit from use of the equation together with imaging experiments over classical experimental approaches and how this could "rescue a once-promising therapeutic class". Unless I am missing the point, I suggest that the authors use a considerably more modest narrative, not only in the abstract.

RESPONSE: Please see our response to Reviewer 1, point 5, in which we describe how we have toned down our claims and added additional data showing that Apomab has relatively low potency on a wide variety of cell lines.

With respect to using population average as opposed to single-cell measurements we have previously described the difficulty of scoring initiator caspase activity using bulk measurements because a small number of cells with very high levels effector caspase activity overwhelm data from a much greater number of cells with initiator caspase activity (Albeck et al. Mol Cell 2008 PMID 18406323, Albeck et al. PLoS Biol 2008 PMID 19053173). We therefore believe that our approach allows a much more precise assessment of DISC function that should be valuable in the development of new DR4/5 agonists.

However we agree with the reviewer that we have overstepped in asserting the "practical value of the equation" and we have therefore edited the manuscript on page 2 (abstract), on page 12 paragraph 3 and page 13 (results) to address this comment. These edits also address comment 5 from reviewer 1.

Reviewer #3 additional important comments:

1) Throughout, the authors should be more specific in their description of the experimental data. For example, results and figure legend related to Fig.1 do not mention which cell line has been used. Information on the number of repeat experiments should be provided. Information on the times of cell death/survival readings should be provided etc.

RESPONSE: We have now edited the text of all figure legends, including figure legends in Expanded view, to address these points. To clarify how many replicates were made for each condition, we added a supplementary table summarizing all experiments and reporting the mean values for k, τ, surviving fraction and classification accuracy. All the raw trajectory data and all derived parameter values are now available on-line and in supplementary materials.

2) On Page 4, it should probably read "processing of ICAD" rather than "ICAD nucleases". Also, XIAP does not bind the catalytic pocket of caspase-9 (see Srinivasula et al, Nature 2001).

RESPONSE: We have edited both points in the text to make these changes.

3) Can the authors clarify whether all cells in the fields of view were analysed? In Fig.1A it seems that all cells respond with FRET ratio changes. Does this mean that non-responders do not exist at the level of caspase-8 activation? `

RESPONSE: All cells in the fields of view were analyzed, but some cells were discarded when the tracking software could not follow them or when an event was not properly classified as either a cell division or a cell death (see Methods). Note that we used a stringent approach in our image analysis. Adjusting the cutoffs for fate classification did not affect the results qualitatively. We clarify this point in the supplemental methods, on page 7 paragraph 2 of the Expanded view.

At 25ng/ml TRAIL (Fig. 1A) all cells exhibit caspase-8 activation to some degree. We now mention this in the legend of Fig. 1A on page 30. Note however, that some non-responders at the level of C8 activation were observed in the case of Apomab treatment or FLIP-overexpression.

4) This comment is related to major comment 1 and the potential problem of having the execution phase contributing to the signals. When using HeLa cells overexpressing Bcl-2 or Bcl-xL, would cells with dFR/dt values above the red line be observed with the same frequency as in the wt cell population for a treatment condition as in Fig.1B (25 ng/ml TRAIL)? This would be a very important control (Fig.7B in this context cannot serve as a control due to the 10-fold higher TRAIL concentration).

RESPONSE: As described above, we have now performed additional studies in Bcl-XL overexpressing cells and observed that dFR/dt values above θ_T are observed with the same frequency as in the wt cell population (Fig. E1B). The new and revised data is show in Figure panels E1B,C and page 8 paragraphs 1 and 2.

5) Fig.E1A would benefit from also showing the signal for the cleaved probe as well as a loading control. Currently it looks like no probe is cleaved at all when overexpressing Bcl-2. Loading controls for other immunoblots should also be included.

RESPONSE: The corresponding immunoblot now shows the lower molecular weight species so that cleaved probe is visible. Loading control immunoblots, 5 in total, have now been added to relevant figure panels. Please note, however, that this does not apply to the immunoblot in Figure E5B which is loaded with FLIP recombinant protein of known concentration (the 6 lanes on the left), and samples obtained from a FACS sort with known number of cells (the 4 lanes on the right).

6) End of page 9, start of page 10: It is well described that the amounts of tBid do not need to exceed molar amounts of Bcl-2 and Bcl-xL. The associated reference from 1993 is outdated. Bcl-2 family signalling itself is non-linear, and small nanomolar amounts of tBid are sufficient to induce MOMP. Also, tBid is a very short lived protein and its stability may very well differ from that of the cleaved probe. The amount of tBid present at any one time may therefore differ considerably from

© European Molecular Biology Organization

the amount of cleaved probe. It therefore seems logical to me that the rate of probe cleavage (i.e. the flux of tBid generation) rather than the final amount of cleaved probe is associated with the life/death decision.

RESPONSE: We agree with the reviewer and also replaced the reference from 1993. In our interpretation, the level of cleaved ICRP is reflective of the total amount of Bid cleaved over time and not the concentration of tBid at a specific point in time. This is reflected throughout the manuscript and we have also edited the results and the reference on page 11 paragraph 1 to reflect this fact.

7) I consider a concentration of 100 nM bortezomib as very high. While this may not cause death within 24 h (not shown), it will surely do so at later times. The authors therefore need to provide information on when survival in their experiments was determined. Also, they will need to clarify that their readouts and modelling only relates to early death through the extrinsic pathway. Furthermore, if bortezomib itself may kill at high rates at later times, the co-treatment scenarios with TRAIL would only reflect a shift of death to earlier times. Would that not beat the purpose of evaluating or optimising ("rescuing") TRAIL receptor-based treatments, since the other drug alone is already sufficiently potent and TRAIL would not be needed?

RESPONSE: Cells were imaged for 22 hr, and we observed almost no more cell death or caspase-8 activity later than 6 hr after TRAIL addition. In order to avoid phenomena not directly related to TRAIL signaling and tracking artifacts due to cell divisions, we focused on data collected during the first 10 hr. This is now clarified in the page 6, paragraph 2 of the results.

We do not find bortezomib at 100 nM to be cytotoxic on HeLa cells at least in the period covered by our experiments (22 hr). Moreover bortezomib has previously been used at this concentration to score the effects on TRAIL-mediated cell death (Laussmann, M.A. et al., JBC, 2012). The measured value of Cmax for bortezomib in patients is 200-300 nM; at such concentrations we would expect sensitive cancers (multiple myelomas primarily) to induce significant cell death via the intrinsic pathway, but we find there is no evidence of this in HeLa cells.

Overall, we think it is most reasonable to interpret our data in terms of the effects of TRAIL and bortezomib on extrinsic apoptosis only, although we do agree that understanding the clinical significance of our data will require more complete single and dual-agent dose-response studies in a variety of cancer cell types. We have edited the text of the results to clarify this point on page 12 paragraph 2 and we also added a figure panel to Fig. E3A, which shows that caspase-8 is not activated by bortezomib alone (also requested by reviewer 1).

8) DR5 is known to rapidly accumulate following proteasome inhibition, and it would be expected to enhance DISC formation and casp-8 activation. Fig.3C indicates that Tau increases whereas k may not be affected. Would that not be counter-intuitive (unless cFLIP levels, which may accumulate as well, cause the delay)? Would such a DR5 upregulation not also (partially) explain the lack of protection through cFLIP-L overexpression in Fig.6A (cFLIP as an enhancer of death)? A reductionist view explaining the effect observed in Fig.3 only by casp-8 stability does not seem to be warranted without comprehensive biochemical analyses.

RESPONSE: Please see our response to Reviewer 2, point 1. As described above, we find that bortezomib treatment does not appreciably change the concentration of DR5 in the period modeled by our equations and landscapes (3 hr following TRAIL administration to maximal C8 activity). Thus, increases in receptor level do not appear to explain the effects of co-administration of bortezomib and DR4/5 agonists. However, in the case of longer-term exposure to these drugs individually and in combination we agree with the reviewer that it will be necessary to consider additional effects of bortezomib. To clarify this point we edited the text on page 12 paragraph 2, added a new figure panel (Figure E3C) and revised the discussion.

9) For the TRAIL 5 ng/ml + Bortezomib treatment data shown in Fig.3C and Fig.E3A there seems to be a discrepancy. In the main paper, most dying cells being placed to the left of the separator line (what is the accuracy here?), while the separation in Fig.E3A appears to be much better (accuracy here 95%). I would expect both accuracies to be equivalent, since the same data are presented?!

© European Molecular Biology Organization

RESPONSE: The same raw data is indeed used for these two figures, however different processed data are graphed and the position of the separating lines are different. In Fig. 3C (a landscape of k vs. τ) the separating line is calculated from EQ3 and the value θ_T is obtained from experiments with TRAIL only, whereas in Fig. E3A *dFR/dt* trajectories are plotted and the separating line is the actual value of θ_T. The precision of the prediction in the *dFR/dt* figure (Fig. E3A) is higher because the prediction is based on direct measurements, whereas in the landscape figure (Fig. 3C) it relies on fitted parameters of the model. Note that although the landscape is less predictive, its parameters yield biological insight – which is why we use it.

We edited the text of the figure legend (page 2, Expanded view) to clarify this point.

10) The authors use the terms "prediction", "classification" and also refer to "accuracy" of the separations. Are these terms interchangeable? Could the authors clarify this, please? In my understanding of the main paper, data for TRAIL 25 ng/ml was used to define the equation, and theta was then used as a separator (predictor?) for all other treatment conditions? In contrast to this, in the supplemental methods instead it says that "theta" was determined from ALL experiments with TRAIL > 10 ng/ml (supplement, page 8). If this is true, the main manuscript is misleading since the equation technically would be a fit across all data rather than a threshold that was subsequently validated by independent data.

RESPONSE: The reviewer is correct – ICRP trajectories obtained from cells exposed to TRAIL were used to define a value of the separator (θ_T) used for subsequent analysis. We actually performed two different sets of calculations. First, we asked whether data obtained from cells exposed to 25 ng/ml TRAIL, "θ_{T25}" were effective in predicting the effects of TRAIL at other doses (Fig E1E). Having demonstrated this to be true, we used all of the TRAIL data (deriving from doses spanning from 10 to 500 ng/ml) to re-compute the value of the separator, referred to as "θ_T". These values differ slightly; a point now made explicitly in the Results and Methods sections.

When describing the ability of θ_{T25} and θ_T to separate cells fate using data obtained with TRAIL as an agonist we used the terms "classification" and "accuracy of the classification". For experiments involving agonists such as Mapatumumab or Apomab, over-expressed FLIP and Bcl-2/XL and co-drugging, we use the value θ_T computed from TRAIL alone. In these cases it is therefore appropriate to use the term "prediction".

We thank the reviewer for helping to clarify this point which has resulted in definition of θ_{T25} and θ_T on page 7 paragraph 2 (Results section).

11) In the discussion (page 17), the authors refer to changes in factors regulating NFkB. A major point would be the widely acknowledged prevention of IkBalpha degradation.

RESPONSE: We added the reference to the effect of bortezomib on preventing IkBalpha degradation in the discussion (on now page 20).

12) Trajectory fitting and Theta determination are very central to the paper, and it would be better to include these methods in the main document.

RESPONSE: We now include both methods in the main document (Methods); this suggestion was also made by reviewer 1 (minor comment #1).

2nd Editorial Decision 15 February 2015

Thank you again for submitting your work to Molecular Systems Biology. We have now heard back from the three referees who accepted to evaluate the study. The reviewers are now all positive on your work and I am pleased to inform you that we will accept your manuscript for publication pending the following minor points:

- Reviewer #1 is asking to somewhat tone down the generality of some claims.

- The link http://lincs.hms.harvard.edu/roux_msb_2015 does not seem to work.
- For long-term archival and if size permits, we would also prefer to host the data and analysis scripts on the journal website.
- If it is reasonable and possible, it would be ideal if key figure panels showing quantitative data could be accompanied by a downloadable source data file (either as csv file, as Excel table or as an zip archive) so that readers can download the data (and potentially the respective analysis scripts) to examine the data, re-analyze it or generate alternative visualization.

Reviewer #1:

The authors have adequately addressed the majority of the issues raised. However, as they only present preliminary data for other cell lines obtained with a different technique, the general applicability remains to be confirmed. Therefore, statements such as "this may help to rescue a once-promising class of cancer therapeutics" in the abstract need to be toned down. Likewise, in the discussion it should be specified that the results were obtained in HeLa cells, not cells in general.

Reviewer #3:

The authors have sufficiently adressed all points raised by the reviewers.

2nd Revision - authors' response 17 March 2015

Thank you for the reviews our manuscript currently entitled "Cell-to-cell variability in overcoming a caspase activity threshold and fractional killing by TRAIL" (MSB-14-5584R). I believe that we have successfully addressed all of the points raised by the reviewers and the points highlighted in your decision letter. Specifically:
- Regarding the suggestion from Reviewer #1 that we "tone down the generality of some claims", we have edited the Abstract (page 2, paragraph 1) and the Discussion (page 19 paragraph 2 and page 20 paragraph 2) to more clearly describe the biological contexts in which our conclusions are likely to be relevant.
- The dead link http://lincs.hms.harvard.edu/roux-molsystbiol-2015 has been corrected (pages 7 and 24), the Web page has been modified to fit the current manuscript and it is now live. We are finalizing a second linked Web page that provides access to individual figure panels and relevant data (see attached screen shots). This page is still on our development server (http://dev.lincs.hms.harvard.edu/explore/trail-threshold-variability/) since we are finalizing some of the links and descriptions of but it will be live by the time proofs are received.
I hope that these Web resources are sufficient. We will continue to extend them, for example, by adding movie files, browseable on-line and downloadable. However, resubmission of our paper has already been delayed several weeks by programming and formatting tasks and we decided it was not wise to wait any longer.
We are of course happy to provide tabular data and software for hosting on the MSB website. Datasets and scripts along with checklists, synopsis, highlights, and visual abstract are now part of the resubmission. Please let us know if you have any problem with file formats (raw and processed data are in a zip file with one subfolder per experiment; figure-related data are in .tsv files). If there is anything else currently on our web site that you would also like to host at MSB, please let us know.
It seems to me that titles are increasingly important in promoting interest in papers that are discovered largely through search. I therefore propose that we change the title of the paper to: "Fractional killing arises from cell-to-cell variability in overcoming a caspase activity threshold"; the running title is now "Cell killing by TRAIL and therapeutic antibodies." This pair better captures the essence of our work and is likely to receive more attention than the old title. Please let me know if you have any objection to this change, or if you have a different suggestion.
